# Digital supportive care in prostate cancer: a systematic review

**DOI:** 10.1007/s00520-026-11192-x

**Published:** 2026-10-17

**Authors:** C. Paterson, D. Coro, J. N. Morris, M. R. Turner, N. Perrotta, M. Godfrey, A. Robertson, J. Christina

**Affiliations:** 1 Flinders University, Caring Futures Institute, Sports Road, Bedford Park, Adelaide, Australia; 2 Central Adelaide Local Health Network, Port Road, Adelaide, Australia; 3 Cancer Council South Australia, 202 Greenhill Road, Adelaide, Australia; 4 University of Canberra, Australian Capital Territory, Canberra, Bruce, Australia; 5 College of Medicine and Public Health, Flinders University, Adelaide, Australia

**Keywords:** Digital, Supportive care, Prostate cancer, Systematic review

## Abstract

**Purpose:**

To map and synthesise the existing evidence on people’s perspectives and requirements for patient-facing digital health technologies for use in prostate cancer care delivery. Understanding the preferences of people with prostate cancer and understanding the barriers and facilitators to maximising engagement with digital supportive care healthcare landscape.

**Methods:**

Four electronic databases were searched to identify suitable studies. All identified citations were imported into Covidence systematic review software for the removal of duplicate records and the study selection process by two reviewers. Methodological quality was appraised using the MMAT, and findings were synthesised narratively using the Human, Organisation, and Technology-fit (HOT-fit) evaluation framework.

**Results:**

Eighty-eight studies were included and represented a total of 29,256 participants affected by prostate cancer. Preferences for digital supportive care emerged across four interconnected domains: (1) mode of delivery, (2) type of content desired, (3) factors enabling engagement, and (4) barriers limiting use. Overall, there was willingness to engage with digital models due to their convenience, flexibility, and immediacy of access. Self-management was enhanced via decision aids, symptom management tools, informational resources, and evidence-based tools to support aspects of psychosocial functioning and emotional wellbeing, physical activity, and sexual health. Several barriers to the implementation and uptake of digital supportive care interventions were identified primarily relating to technology access, device compatibility, digital literacy, and emotional readiness.

**Conclusions:**

Digital models in prostate cancer care were acceptable and valued for their convenience, flexibility, immediacy of access, tailored content, clinician support, and interactive features, and facilitated self-management through decision-making, symptom management, and informational and psychosocial support. Interpreted through the HOT-fit framework, their value depended on the fit between the technology, its users, and the care setting rather than on technological sophistication alone. Limited attention has been given to digital sexual wellbeing support, and further research is needed on engagement among people with low digital literacy, non-English speakers, and lower-income populations, and on the long-term sustainability of digital models.

**Supplementary Information:**

The online version contains supplementary material available at https://doi.org/10.1007/s00520-026-11192-x.

## Introduction

People affected by prostate cancer can experience high symptom burden [1] and report a range of unmet supportive care needs despite regular reviews with healthcare professionals [2–5]. Often people have limited access to anticipatory models of care [6] that provide a tailored needs-based approach to personalised care and provide supported self-management, while improving health service resource utilisation [7]. The expansion of digital health technologies in healthcare services has contributed towards alleviating the increased cancer burden while driving improvements in quality care, accessibility, and cost-effectiveness [8]. Digital health approaches are essential in cancer care, with growing numbers of cancer survivors [7], and recognition of their complex, long-term healthcare needs requiring a high level of self-management [9]. Digital health is a term used to describe information technology, digital systems, information systems, electronic health and medical records, mobile health and applications, telehealth, telemedicine, wearables, robotics, and artificial intelligence (AI) [10].

Evidence has underscored numerous benefits of digital health technologies and healthcare delivery, including remote monitoring of patients’ symptoms and conditions [11]; the provision of electronic decision support, resources, and self-management interventions; longitudinal population monitoring; health behaviour modification in primary and secondary prevention; and the facilitation of distant patient–clinician communication [12]. The incorporation of digital health technologies into national cancer systems worldwide has been identified as a strategical lever and key priority [13]. Further, digital technology applications in cancer care can increase equity in access for patients in rural and remote geographical locations, and those living with socioeconomic disadvantages by mitigating geographical and socioeconomic disparities in cancer outcomes [14].

Incorporating patients’ perspectives throughout the development of digital health tools is a critical determinant for the successful uptake of these technologies in cancer services [15]. The saturation of telephone consultations and interruptions to psychosocial support provision during COVID-19 highlighted the need for coordinated and user-friendly digital cancer care, with embedded strategies to reduce feelings of anxiety, isolation, and uncertainty for patients [16]. Understanding end-users’ preferences and needs for digital innovations is fundamental to increase their feasibility, acceptance, and long-term adoption in cancer populations. Moreover, a recent commission for the Lancet [17] points towards the importance of digital formats to support the rapidly changing landscape of modern communication to connect people with prostate cancer more rapidly to their care teams.

Despite the value of encompassing end-users’ perspectives into digital designs, there is limited information available in relation to the needs and preferences for people affected by prostate cancer, and what implementation factors promote adoption and maintenance [18].

However, the benefits of digital health are not realised equally across populations. The digital divide, which includes systematic disparities in access to devices, reliable internet connectivity, and the skills to use them, including variable digital health literacy, can limit engagement with digital supportive care and risks widening existing inequities in cancer outcomes [19]. These barriers can disproportionately affect older adults, people in rural and remote areas, and those from socioeconomically disadvantaged or culturally and linguistically diverse backgrounds [20]. Acknowledging these limitations is essential to ensure digital innovations enhance, rather than entrench, disparities in supportive cancer care.

The purpose of this systematic review was to provide a critical overview of the available evidence regarding the needs and preferences of people with prostate cancer for person-facing digital health technologies and identify factors which impacted implementation of digital technologies in their care. To our knowledge, this is the first systematic review to map and synthesise the existing evidence on people’s perspectives and requirements for patient-facing digital health technologies for use in prostate cancer care delivery. Understanding the preferences of people with prostate cancer and addressing key barriers are essential to maximising engagement with digital supportive care and improve health outcomes.

## Methods

A systematic review was conducted according to PRISMA (Preferred Reporting Items for Systematic Reviews and Meta-Analyses) guidelines [21]. The review protocol was registered with PROSPERO (CRD42024570061).

### Search strategy

Electronic databases included CINAHL, MEDLINE (EBSCO host), SCOPUS, and Web of Science Core Collection. Databases were searched using an advanced search strategy (Supplementary File 1). The reference lists of papers deemed highly relevant to the current review were screened for additional literature; however, no additional studies beyond those identified by the search strategy were found through this method. Only studies published in English were examined. There were no restrictions relating to published dates and databases. The search occurred between database inception to July 2024.

All titles and abstracts and full-text articles were double screened independently by two authors for eligibility against eligibility criteria. Conflicts were resolved with discussions held by reviewers to reach consensus. The screening process was managed using Covidence Systematic Review Software (www.covidence.org). Reasons for exclusion at full-text were recorded and are reported in the PRISMA flow diagram.

### Eligibility criteria

#### Population

Studies reporting mixed populations (e.g. individuals with prostate and other cancer types or prostate cancer patients and partner dyads) were only included when data specific to prostate cancer patients were reported separately, with only the prostate cancer patient data extracted for analysis.

#### Intervention

All types of studies were included if they were primary research studies with full-text articles that evaluated a supportive care intervention delivered via digital delivery methods (e.g. internet, telephone, apps, wearable technology) for people with prostate cancer.

#### Comparison

Any comparator or none (no restriction; not applicable for qualitative studies).

#### Outcomes

Patients’ reported preference measures regarding patient-facing digital technologies and related implementation factors. Preferences were defined as any data pertaining to inclination to receive digital care that included (but were not limited to) adherence, engagement, opinion, attitude, experience, or uptake.

#### Study design

Articles were excluded if they were review articles (systematic, scoping and narrative), commentary articles, letters, study protocols, theses, case-reports, editorials, or book chapters. Studies were also excluded if they excluded prostate cancer populations or reported on clinicians’ perceptions of the use of patient-facing digital technologies rather than the person with prostate cancer.

### Data extraction

Two reviewers independently extracted relevant data on each included study using a purpose-built data extraction form, including the following variables: author(s), date of publication, study design, study population, sample size, technology used, and purpose of technology use. Data on users’ perceptions of technologies were also extracted using the same form and were categorised based on a modified version of the technology evaluation categories of the Human, Organisation, and Technology-fit (HOT-fit) evaluation framework for Health Information Systems [22]. These categories included perceptions relating to the quality of the technological system, its content/available information, its service quality, and other relevant characteristics and features reported by people with prostate cancer. We also extracted data in relation to the Reach, Effectiveness, Adoption, Implementation, and Maintenance (RE-AIM) framework to understand barriers and facilitators to implementation across the digital landscape [23]. The results of data extraction were compared, and any disagreements between reviewers were resolved by consensus.

### Data synthesis

A narrative synthesis approach was used to summarise the evidence [24]. This process involved a tabulation of primary research studies, identifying similarities and differences within and between studies, and seeking explanations for these differences. The analysis implicated the following steps: data reduction and subgroup classification based on the needs and preferences of digital technologies, with a focus on barriers and enablers to implementation. Then, a narrative data comparison was performed which was an iterative process of making comparisons and identifying relationships, and, finally, drawing conclusions. Data synthesis was conducted by two review authors.

### Assessment of risk of bias in included studies

The methodological quality of studies was assessed using the Mixed Methods Appraisal Tool (MMAT) [25], enabling inclusion of a range of study designs. Each study was independently quality appraised by at least two reviewers, with any disagreements resolved through discussion.

## Findings

**Fig. 1:**
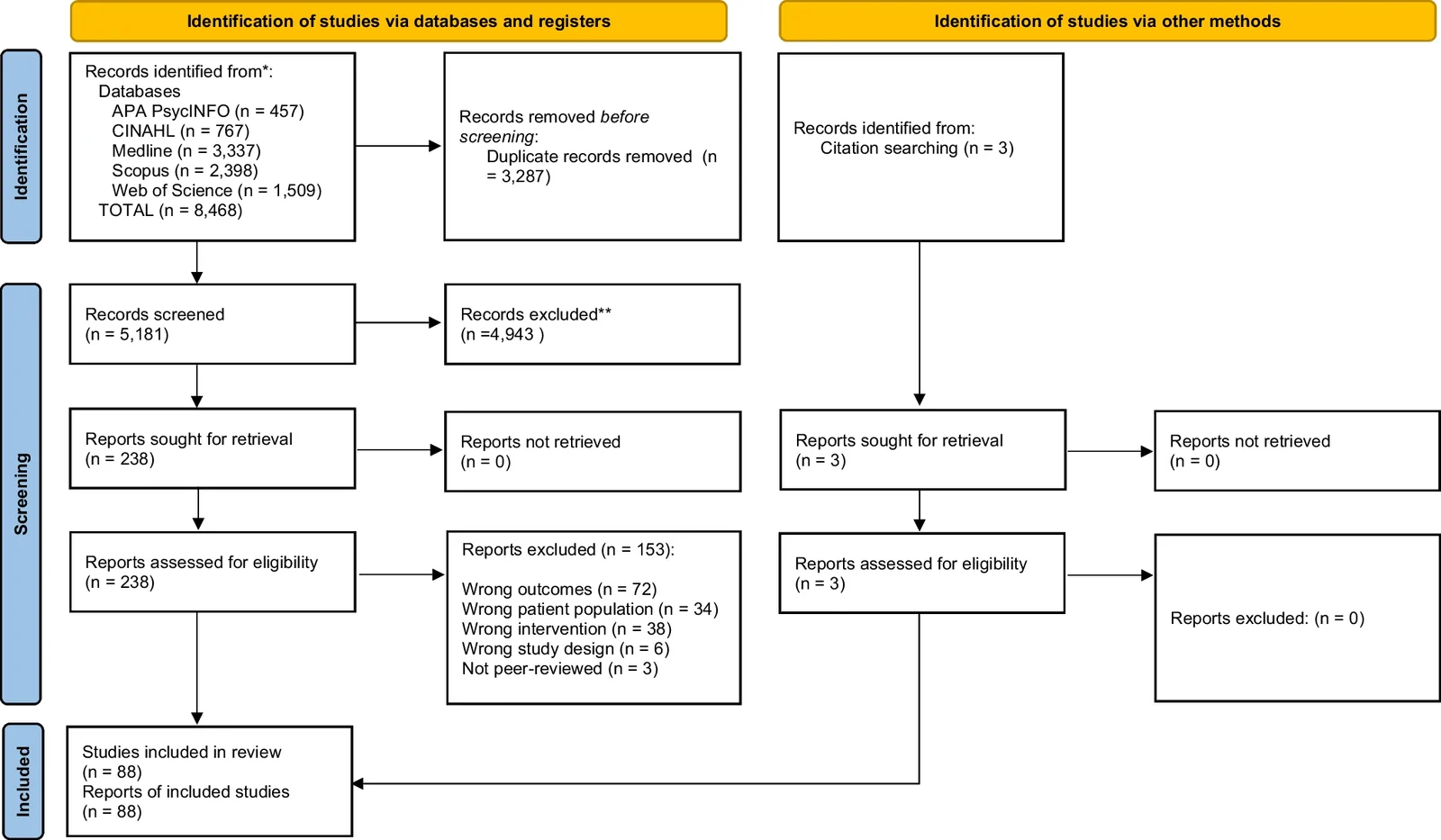
PRISMA

Of the 5184 articles identified for screening, a total of 4943 were excluded, and 88 studies included in this review (Fig. 1). Studies were published between 1996 and July 2024, with most conducted in the USA (*n* = 31), Canada (*n* = 16), and Australia (*n* = 11). Sample sizes ranged from 9 to 11,290, representing a total of 29,256 participants affected by prostate cancer; a comprehensive overview of the included studies is available in Supplementary Table 2. Overall, the methodological quality of included studies was moderate (Table 1). Methodological quality was moderate, with qualitative and mixed-methods studies rated highest and randomised controlled trials lowest, the latter driven chiefly by unavoidable limitations in assessor blinding. The concentration of “No” ratings on assessor blinding (RCTs) and confounder control (non-randomised studies) should be considered when interpreting the certainty of the synthesised findings.

**Table 1.**
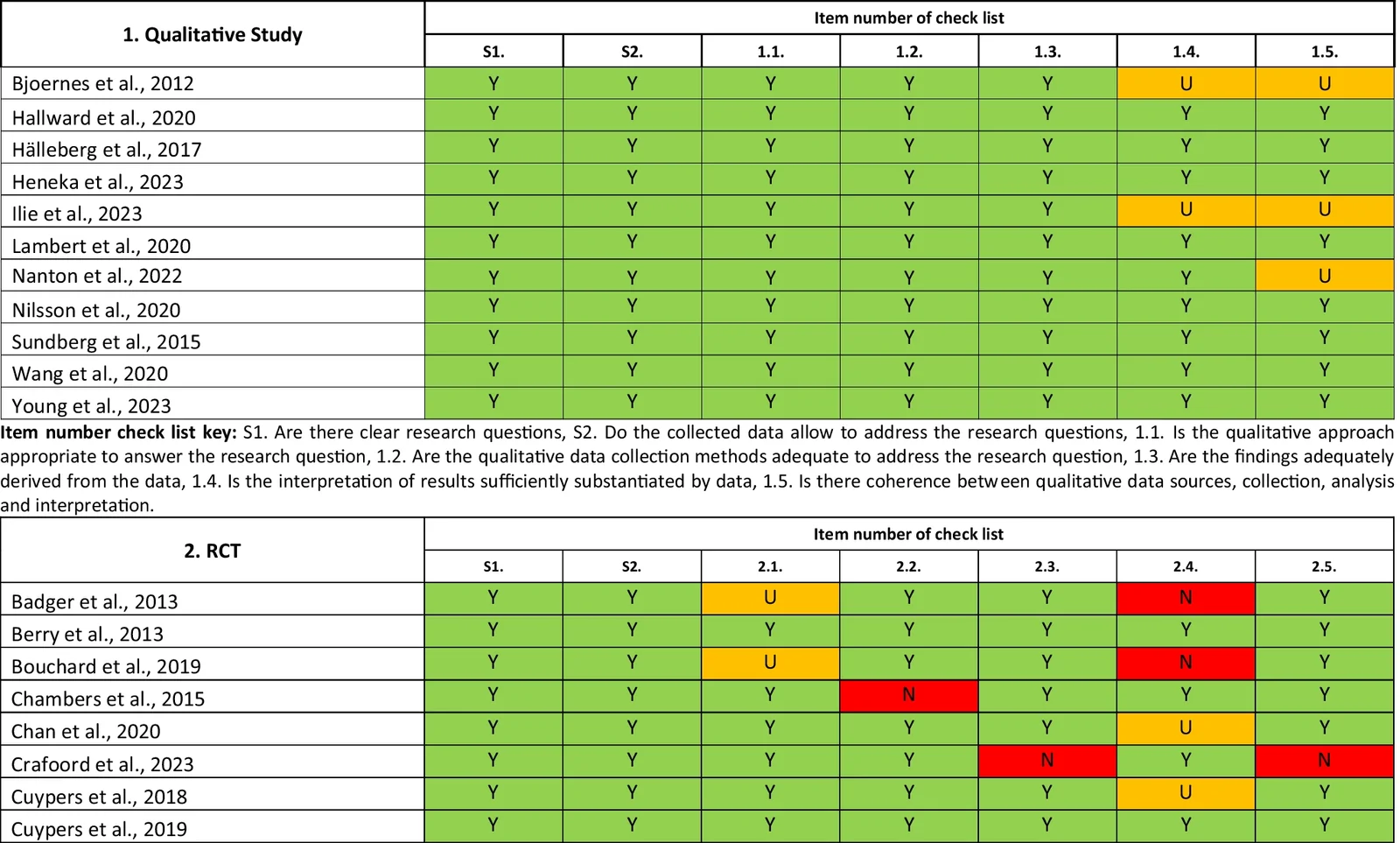

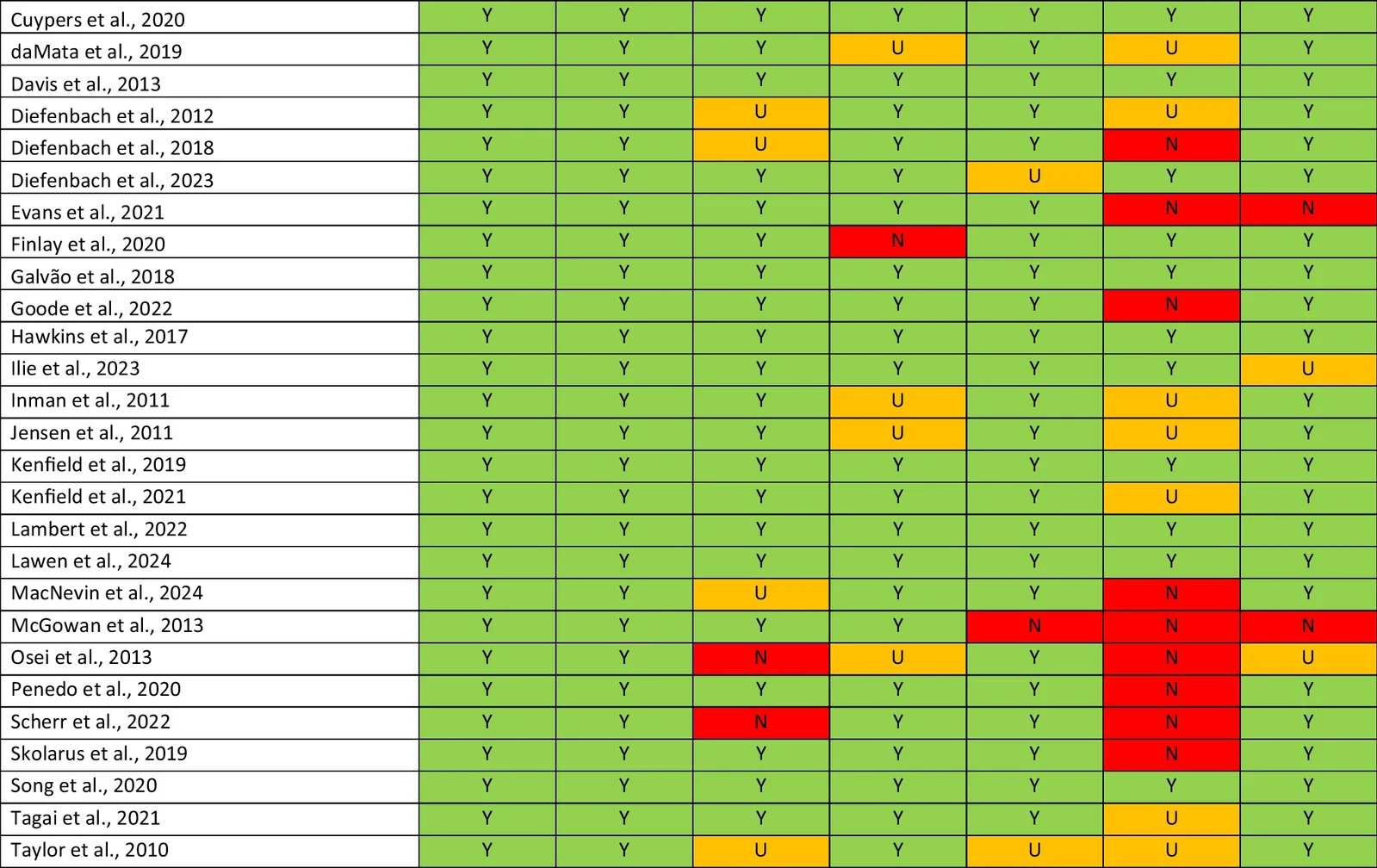

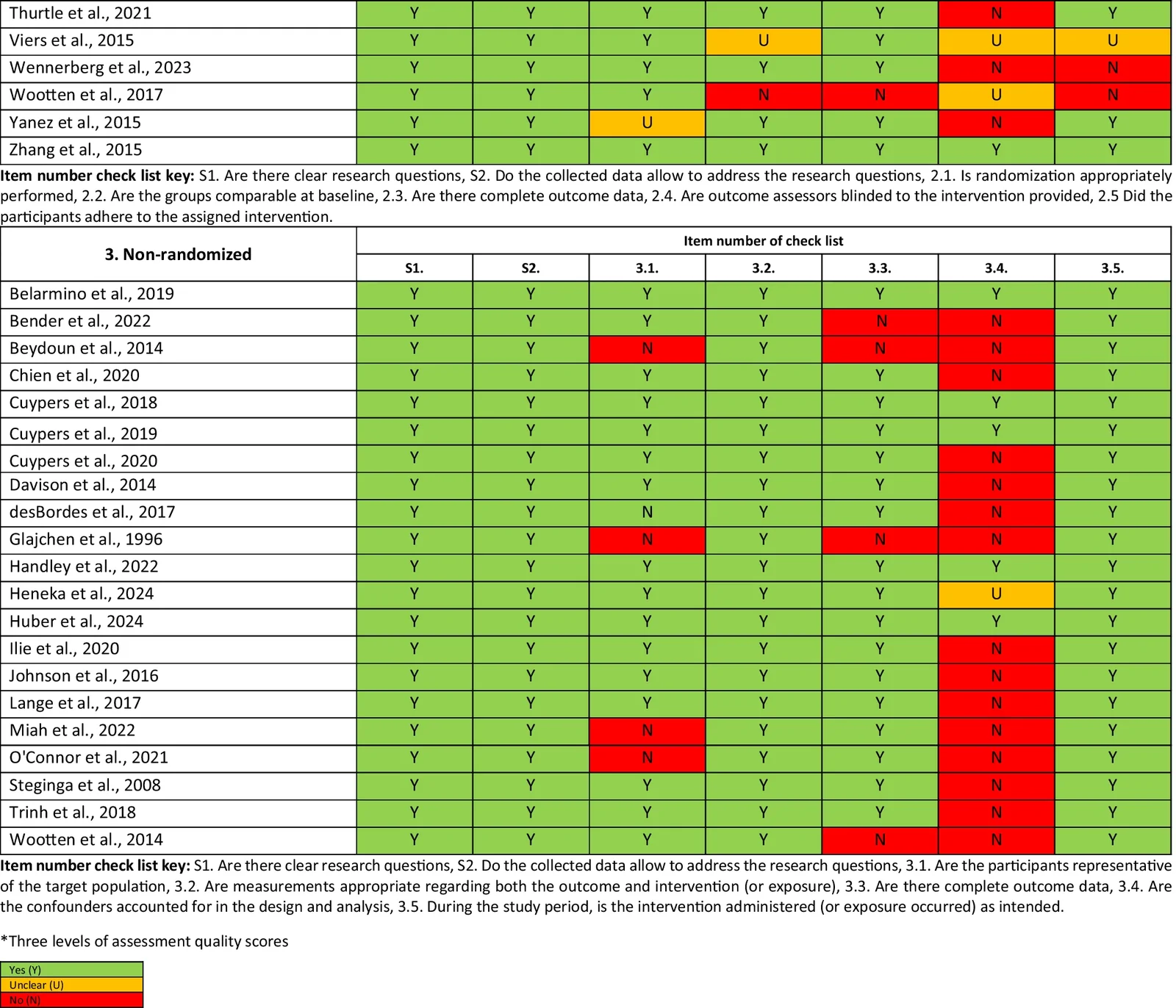

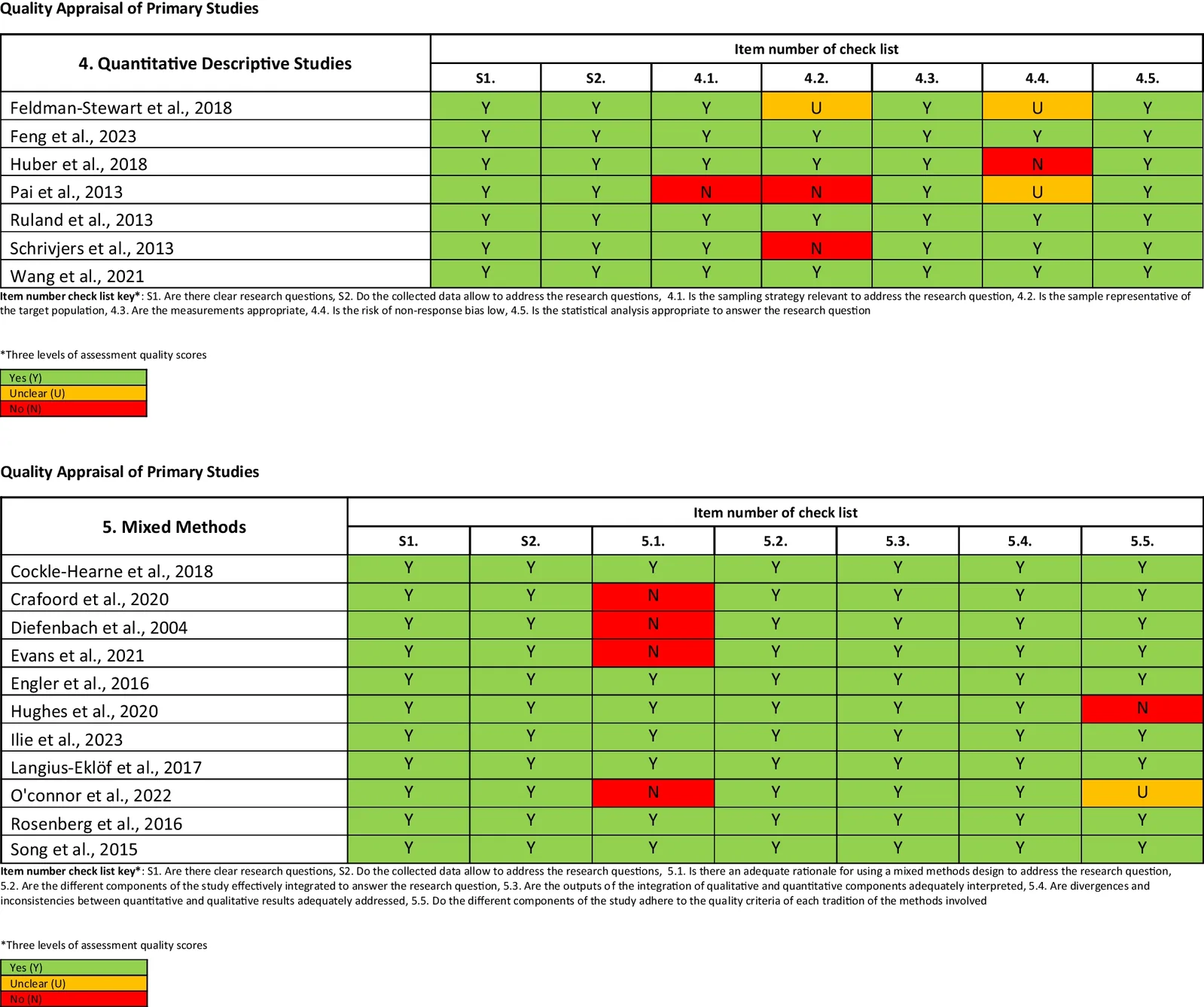
Quality appraisal of primary studies

**Fig. 2:**
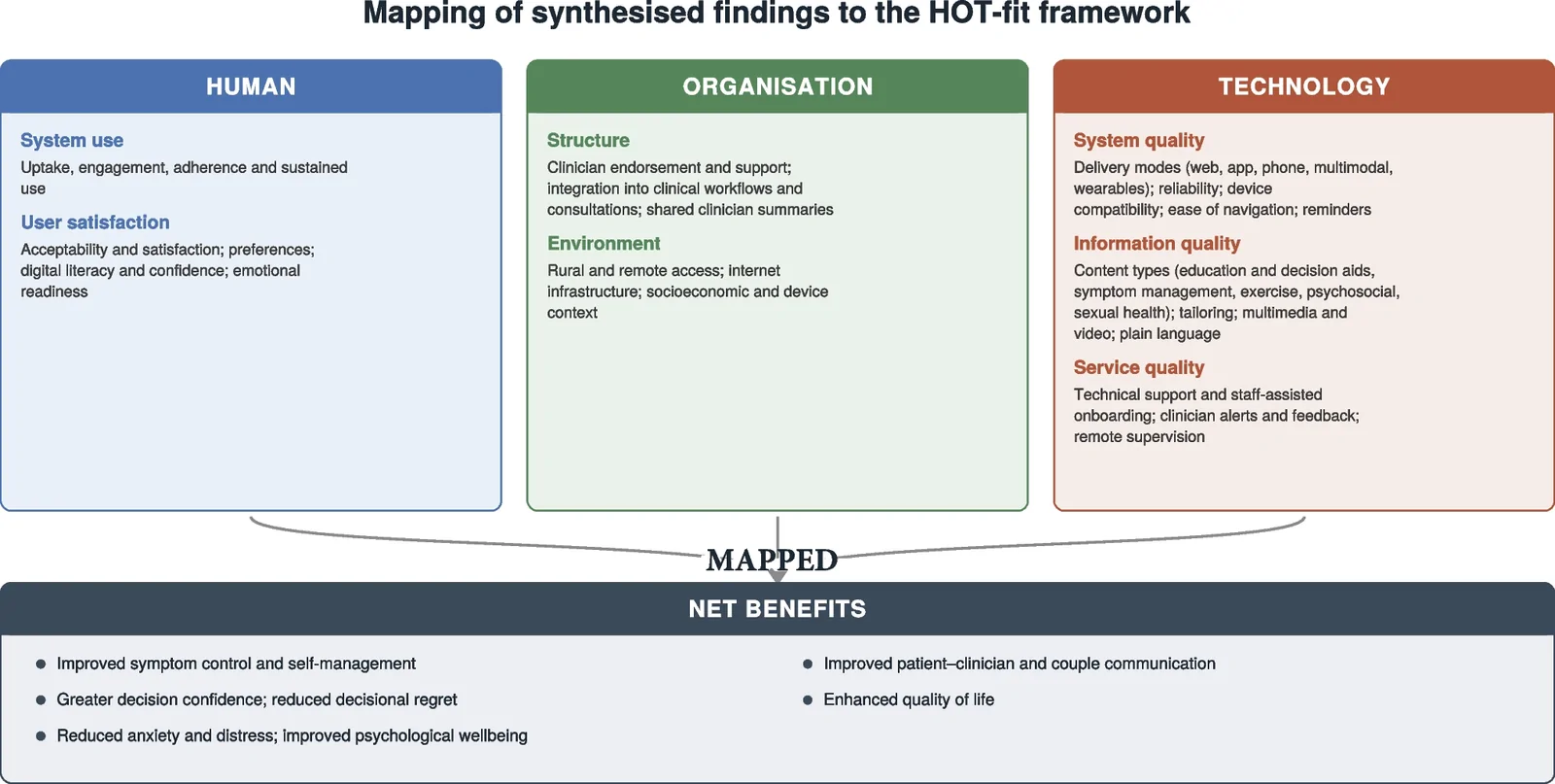
Summary of digital supportive care delivery modes

**Fig. 3:**
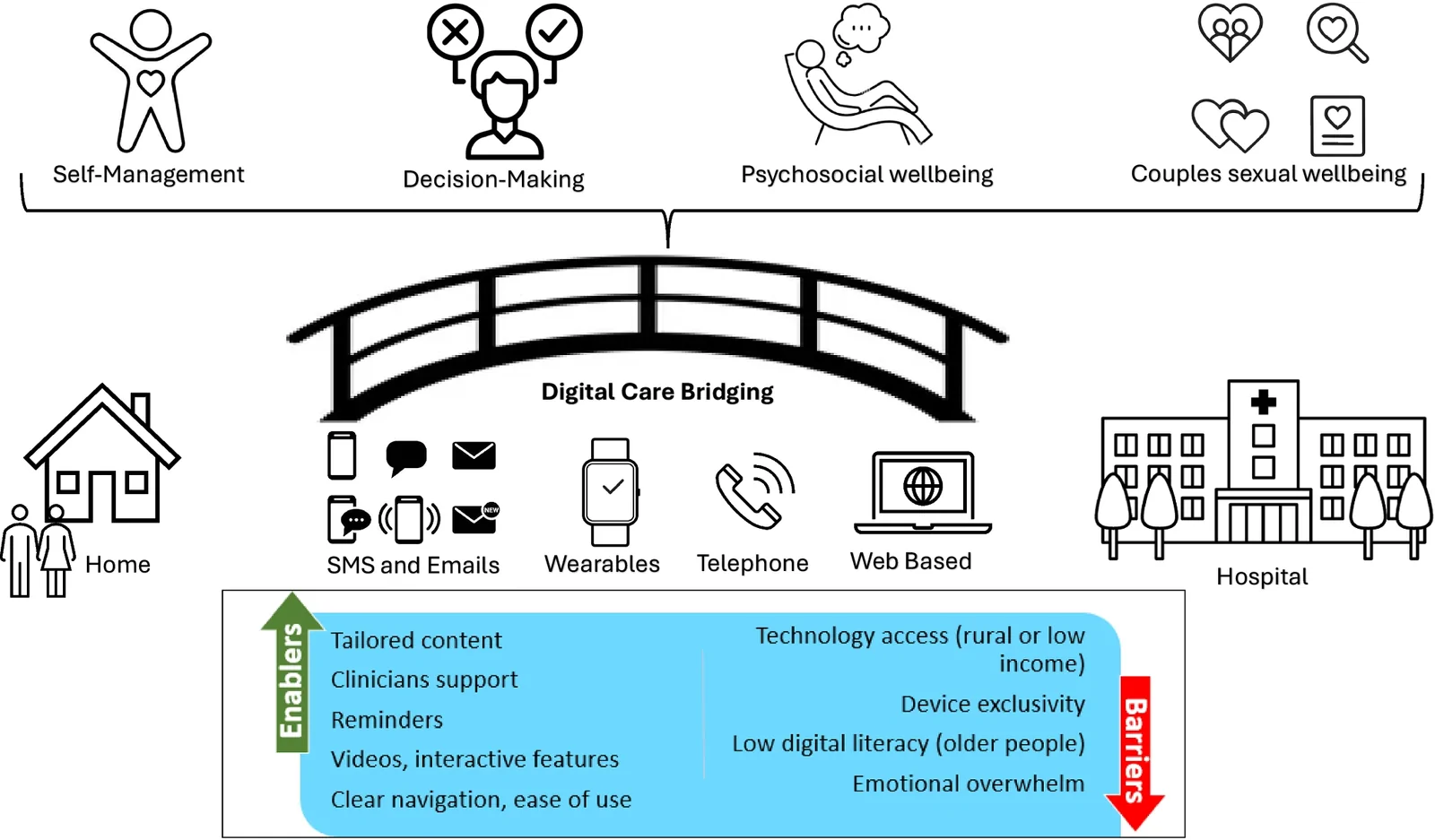
Digital supportive care

Preferences for digital supportive care emerged across four interconnected domains: (1) mode of delivery, (2) type of content desired, (3) factors enabling engagement, and (4) barriers limiting use. These four findings were synthesised through the Human–Organisation–Technology-fit (HOT-fit) framework. Delivery modes and content types correspond most closely to the *Technology* dimension, reflecting system quality (the usability, reliability, and accessibility of the delivery platform) and information quality (the relevance, personalisation, and format of the content). Enablers and barriers spanned all three fit dimensions: technical support, clinician alerts, and remotely supervised interaction reflected *service quality*; clinician endorsement and integration into clinical workflows reflected the *Organisation* dimension (structure and environment); and users’ digital literacy, emotional readiness, engagement, and satisfaction reflected the *Human* dimension (system use and user satisfaction). The benefits participants derived, improved symptom control, decision confidence, psychological wellbeing, and quality of life, which were mapped (Fig. 2) to the net benefits dimension and (Fig. 3) for digital supportive care model.

### Delivery modes of digital supportive care (technology—system quality)

Forty studies [26–64] evaluated web-based platforms, indicating this was the most commonly assessed and widely implemented digital delivery mode. Participants valued web-based interventions for their flexibility, structured presentation of information, and ability to be accessed at home or in clinical settings. Web-based platforms were used to deliver educational content [18, 26, 34, 35, 49, 51, 54, 56, 57, 60, 63, 65–69], decision-making support [55, 59], symptom monitoring and self-management [18, 47, 48, 54, 56–59, 63, 64, 70, 71], and peer-based resources [29, 35, 52, 61, 72]. Preference for web-platforms was strongest when integrated into clinical workflows, such as being introduced during consultations or generating summaries that could be shared with clinicians, which enhanced perceived legitimacy and usefulness of the digital application [27, 29, 31, 73].

Studies [74–78] assessed mobile applications (apps), revealing them to be highly valued for real-time interactions, symptom tracking, medication or exercise reminders, and automated alerts to healthcare providers. Apps such as symptom monitoring tools and postoperative recovery platforms were perceived as convenient, engaging, and supportive of self-management, particularly during active treatment phases [74]. However, device exclusivity (e.g. smartphone- or iPhone-only applications), the need for ongoing technical support, and varying levels of digital confidence constrained uptake and sustained use [74, 79]. Few studies [76, 78] indicated that those with lower digital literacy often required staff-assisted onboarding.

Phone-based delivery models included nurse-led counselling [80, 81], postoperative educational support and follow-up [82], symptom management [83], and physical activity intervention [84]. Nine studies [72, 80–82, 84–88] reported that these models were preferred by older adults, individuals with limited internet access, and those living in rural or remote areas [89, 90]. Phone-based interventions were reported to be more accessible and less cognitively demanding than digital platforms [82, 90]. Participants valued the interpersonal connection and immediacy of verbal communication, particularly for decision-making support [81] and psychosocial and sexual health support [79, 87, 88].

Models combining multiple modalities (e.g. web-based tools supplemented by phone support, print materials, online booklet, videos, wearable fitness tracker, email, or face-to-face interactions) demonstrated the greatest acceptability and inclusivity [31]. Twenty-eight studies [18, 31, 36, 65–71, 73, 77–79, 89, 91–100] reported that multimodal approaches allowed participants to engage with content aligned with their preferences and capabilities, while also supporting transitions across the cancer care continuum. Technology-based tools such as websites and apps were used for information and symptom monitoring [18, 54, 58, 73, 101], whereas phone or in-person contact was preferred for couple-based sexual and psychosocial consultation [79, 87] and psychological distress and self-management support [81–84, 88, 89]. Hybrid models also mitigated digital exclusion by offering alternatives for users unable or unwilling to engage with fully digital interventions.

Simple SMS and email reminders were frequently cited as effective adjuncts across delivery modes [36, 54, 69, 92, 102]. These low-burden strategies supported adherence, prompted engagement, and reinforced behaviour change, particularly in exercise, symptom monitoring, and follow-up activities. Participants generally viewed reminders positively when they were timely and non-intrusive [57].

Studies [71, 97, 103] reported that wearable devices integrated into exercise or lifestyle interventions provided real-time feedback and motivational support, particularly among participants who were already physically active or motivated to engage in exercise. People who received combination tailored online information, wearable devices, and coaching calls had higher lifestyle intervention engagement and satisfaction compared to those who received access to general online information only [65, 68].

Only two studies [65, 68] identified that a combination of paper-based resources with digital interfaces was a valued delivery mode for a subset of participants, particularly older individuals, those with low digital confidence, and patients who experienced emotional distress. Paper-based materials were perceived as reliable, easy to revisit, and less overwhelming and were often preferred for decision aids and educational content.

### Type of digital content (technology—information quality)

Digital supportive care interventions most frequently targeted educational and decision-making support, symptom management, psychosocial wellbeing, couple-based and sexual health, and post-surgery follow-up. See Fig. 3 for summary of digital supportive care delivery modes.

#### Educational information and decision-making support

Educational information and decision-making support tools were consistently reported as highly valued, particularly when personalised to individual diagnosis and treatment context. Seventeen studies [34, 37, 44, 57, 59, 65, 66, 69, 82, 85, 86, 90, 95, 104, 105] reported that educational information focusing on prostate health, treatment and side effects, and survivorship was the key content of the various interventions.

Decision-making support content was predominantly delivered through web-based patient decision aids (DAs) designed to facilitate shared decision-making at key treatment junctures [31, 32, 66, 91]. People consistently preferred content that provided clear, side-by-side comparisons of treatment options, including anticipated benefits, risks, and side-effect profiles. Visual risk communication tools such as graphs and probability diagrams were particularly valued for clarifying trade-offs between oncological control and functional outcomes and useful during consultation discussions [31]. Participants reported that DAs enhanced decision confidence; reduced confusion and anxiety; improved understanding of diagnosis, treatment options, and potential side effects [37, 46, 67, 104]; and reduced decisional-regret [81] due to the complexity and breadth of prostate cancer treatment options [99]. Collectively, these findings indicate that people preferred structured, visually clear, and accessible decision-making support integrated with communication preparation.

#### Symptom management

Symptom management content was consistently perceived as valuable, practical, and informative across the included studies. Interventions incorporating tailored self-management advice and structured symptom reporting enhanced patient engagement in active symptom management [75]. People reported increased confidence in recognising and managing treatment-related side-effects and increased reassurance when digital platforms facilitated communication with healthcare professionals [75]. High levels of self-reported symptom management effectiveness and increased engagement with healthcare providers were observed in app-based monitoring content [42]. Digital symptom management content was also described as very helpful, with people reporting that tailored advice contributed to reduced symptom burden, improved understanding of treatment side-effects, and quality of life [57, 84, 106]. Symptom management content was most effective when personalised, actionable, and supported by clinician or peer interaction [84], reinforcing its central role in optimising self-management in community settings.

#### Exercise and lifestyle

Exercise-focused content was consistently among the most frequently accessed and positively evaluated components of digital supportive care interventions [57, 65, 86]. Aerobic and resistance exercise interventions were associated with improvements in physical and emotional wellbeing that were observed at 3-month follow-up [65, 86]. Participants valued the flexibility of home-based exercise delivery, reporting that the ability to exercise in the comfort of their own home and access a starter home exercise kit facilitated participation and improved fitness [68, 86, 107]. Participants prioritised functionality and ease of navigation over aesthetic appeal and perceived the information as credible and useful. Video demonstrations improved confidence in completing exercises safely and independently at home [78, 107]. Exercise content was most effective and preferred when personalised, visually demonstrated, remotely supported, and designed with simple, functional interfaces that facilitated safe home-based participation.

#### Psychosocial and peer support

Psychosocial content in digital interventions was primarily delivered through web-based cognitive-behavioural therapy (CBT) and stress management programmes incorporating structured educational modules, peer video narratives, and moderated discussion forums. These interventions were associated with improvements in sexual satisfaction [61] and reductions in anxiety and distress [70, 81] with perceived emotional benefits [29]. Participants reported high satisfaction with multimedia formats, describing the information as useful and indicating willingness to recommend the interventions to others [28, 63, 79]. In addition, partners participating in psychosocial interventions demonstrated significant improvements in mental healthcare-related quality of life [79] and improved communication among couples [40, 48]. Some participants reported it was important to access psychosocial support outside of formal clinical settings, underscoring the accessibility of digital delivery in the community setting [77]. Psychosocial digital interventions were most effective when structured, interactive, and integrated with facilitated peer and clinician support.

#### Couple-based and sexual health

Couples-based and sexual health content [50, 108] were less frequently represented among the included studies but addressed an important relational dimension of supportive care. Participants reported improved understanding of how prostate cancer treatment affected their sex life [50]. Sexual health-focused content increased uptake of medical treatments for erectile dysfunction at 12 months, although this did not consistently translate into improved sexual or relationship outcomes [87]. Sexual dysfunction content was among the most frequently accessed educational resources by patient–partner dyads, highlighting high informational demand in this domain [56]. The uptake was moderated by emotional readiness and comfort discussing intimacy-related concerns, underscoring the need for sensitive framing and optional partner involvement. Couples-based and sexual health digital interventions addressed a clinically significant supportive care domain, and effectiveness was enhanced when personalised, relationally sensitive, and integrated within routine clinical pathways with opportunities for guided discussion.

### Enablers to digital supportive care implementation (technology, organisation, and human)

Several consistent enablers were identified that enhanced engagement, acceptability, and sustained use of digital supportive care interventions. Tailored content emerged as a central implementation facilitator. Interventions personalised to clinical characteristics, symptom profiles, or treatment stage were perceived as more relevant and useful, leading to greater engagement [30, 31, 49, 72, 75, 76, 79, 100]. Tailoring symptom management domains or decision-support outputs to individual priorities further enhanced perceived benefit and usability [37, 94]. Reminders and structured prompts supported adherence, particularly in symptom tracking and exercise programmes. Automated notifications, daily reporting prompts, and integrated reminders facilitated consistent engagement during active treatment [74–77, 86, 95, 101]. Goal-setting features and behavioural tracking further reinforced participation [75, 76].

Clinician endorsement and support significantly strengthened uptake and trust. Decision aids and digital platforms introduced or discussed during consultations were associated with higher utilisation and improved shared decision-making outcomes [30, 31, 46]. Symptom-monitoring platforms that incorporated clinician alerts and feedback increased perceptions of safety and engagement [41, 75]. Remote supervision by exercise professionals also improved adherence and feasibility of home-based interventions [68, 74].

Video and interactive features-based content was consistently preferred over text-only formats, particularly for exercise and educational modules. Participants reported that video demonstrations increased confidence in self-management strategies and improved perceived clarity of information [26, 34–36, 49, 67, 99]. Multimedia content was also associated with high satisfaction and perceived usefulness in psychosocial and educational interventions [79]. Interactive features, including symptom reporting dashboards, moderated discussion forums, and communication training components, enhanced engagement and perceived relevance [31, 87, 99]. Interventions combining informational content with communication preparation yielded stronger shared decision-making outcomes than information-only approaches [99].

Clear navigation and ease of use were critical implementation enablers. Participants prioritised simple, functional interfaces over aesthetic complexity, particularly among older adults and those with lower digital literacy [107]. Platforms described as intuitive, concise, and written in plain language were associated with higher satisfaction and continued use [31, 32, 51, 102, 107]. In summary, digital supportive care interventions were most successfully implemented when clinician-supported, personalised, interactive, multimedia-enhanced, and designed with simplicity and usability at the forefront. These enablers appear central to optimising reach, equity, and sustained engagement in prostate cancer supportive care.

### Barriers to digital supportive care implementation (technology, organisation, and human)

Several barriers to the implementation and uptake of digital supportive care interventions were identified across the included studies, primarily relating to technology access, device compatibility, digital literacy, and emotional readiness.

Technology access constraints, particularly among rural and lower-income populations, limited equitable participation in digitally delivered interventions. Studies noted that inconsistent internet connectivity, technical difficulties, and limited access to reliable devices reduced feasibility for some users, particularly those living outside metropolitan areas [26, 28, 52, 53, 61, 67, 79, 103, 105, 108]. Participants in rural settings expressed greater reliance on telephone-based modalities, rather than internet-dependent platforms [46, 89]. Device exclusivity further restricted access in certain interventions. Platforms that require specific operating systems or smartphone ownership pose barriers to participation for individuals without compatible devices, thereby limiting inclusivity and scalability [42, 75]. Such structural limitations were particularly problematic for older adults and socioeconomically disadvantaged groups.

Low digital literacy especially among older participants was consistently identified as a barrier to engagement. Complex interfaces, multi-step navigation, or dense informational content reduced usability and confidence in independent use [31, 36, 51, 64, 66, 67, 73, 102]. Participants with lower digital familiarity preferred simpler, more intuitive formats and in some cases defaulted to traditional or telephone-based support options [36].

Emotional overwhelm and psychological distress also moderated uptake and sustained engagement. Elevated anxiety, depression, or cancer-specific distress were associated with reduced satisfaction and lower perceived benefit from digital decision-support tools [30, 31]. Stigma surrounding psychosocial support and discomfort discussing sensitive topics such as sexual health further reduced participation in some interventions [87].

## Discussion

To our knowledge, this is the first systematic review on the use of preferences of digital interventions across the cancer care continuum among people affected by prostate cancer. Overall, there was willingness to engage with digital models due to their convenience, flexibility, and immediacy of access. Digital platforms facilitated self-management, which is an essential model of care given increasing survival rates and a growing population who require support across a protracted cancer continuum [109]. Self-management was enhanced via decision aids, symptom management tools, informational resources, and evidence-based tools to support aspects of psychosocial functioning and emotional wellbeing, physical activity, and sexual health. Patient education is a common offering in digital health [12], which is unsurprising given the simplicity and likely low-cost transfer of education content to an online format. Notably, this review highlighted a strong preference towards user-tailored content to increase perceived relevance of and engagement with the digital intervention. Personalising content based on user characteristics and clinical data requires additional investment [110]. While preliminary costs of establishing digital care are significant, evidence suggests they may prove more cost-effective in the long term [111]. More data are needed to determine the level of personalisation required to sustain user engagement and thus ensure ongoing viability of the programme, while balancing the associated financial investment.

The financial viability of digital programmes may too be impacted by the preference for multimodal interventions that were favoured by participants across reviewed studies. This is particularly relevant for digital interventions with a telehealth component or those requiring clinician response, which impose additional resource burdens. Indeed, this review indicated that people with prostate cancer prefer a degree of human interaction, such as through clinician or peer-support. This aligns with existing evidence about the significance of peer and healthcare professional support during a prostate cancer journey [112, 113]. Peer-support integration may be feasible given its low cost [114] but greater constraints accompany healthcare professional involvement. This points towards alternative approaches to meet this need, such as through AI and particularly, chatbots. Despite the rapidly evolving landscape of AI, a recent systematic review on the efficacy of chatbots in digital health highlighted that despite their potential to improve digital health delivery, there is a distinct lack of clinical evidence and standardised evaluation methods [115, 116]. Across the studies included in this review, there was a distinct lack of AI technologies to support people with prostate cancer. Investigations building on the research by Feng et al. [115] are warranted to understand the value of chatbots to support people with prostate cancer and in cancer care generally.

Digital health models offer confidentiality and anonymity, which may be particularly valuable for people with prostate cancer who often experience stigma related to their condition [117]. Given the significant impact of prostate cancer on sexual functioning, and the shame or stigma that can accompany these symptoms, digital platforms may provide a more accessible and comfortable way for people to engage with information and support on these topics. Despite this potential, few studies include couple-based or sexual health content, highlighting a clear gap and an important area for further development.

Interpreting these preferences through the Human–Organisation–Technology-fit (HOT-fit) framework [22] clarifies why some digital models were valued and sustained while others were not. Consistent with the framework’s premise that net benefits arise from the alignment, meaning the *fit*, between technological, human, and organisational factors rather than from any single dimension, this review found that the technical sophistication of a platform rarely determined its acceptability. Rather, acceptability and sustained engagement depended on the extent to which a technology suited its users’ capabilities and circumstances and the setting in which it was delivered in cancer care.

Within the Technology dimension, participants prioritised information quality and service quality over system complexity: tailored, plain-language content, multimedia demonstrations and options, and responsive technical or clinical support were valued more highly than feature-rich but impersonal platforms. Preferences aligned with the Human dimension were equally decisive, with system use and user satisfaction shaped by digital literacy, confidence, and emotional readiness, such that identical interventions were experienced very differently by older adults, those with limited digital confidence, or those in distress. The Organisation dimension emerged as a critical enabler: interventions embedded within clinical workflows were vital, and introduced during consultations, generating clinician-shared summaries, or supported by clinician alerts and remote self-management support, were consistently perceived as more legitimate and were more sustainably adopted, whereas those delivered in parallel to routine care showed declining engagement over time.

Viewed together, these findings suggest that the net benefits of digital prostate cancer supportive care, improved self-management, decision confidence, symptom control, and quality of life were realised when technological, human, and organisational elements were aligned and were attenuated when they were not. Poor fit was evident where sophisticated platforms neglected users’ digital literacy, where device exclusivity or unreliable connectivity excluded rural and lower-income groups, or where interventions lacked the organisational integration needed to sustain use. This framing reinforces that the central challenge for digital prostate cancer care is not technological capability per se, but designing for fit: matching the mode, content, and support of an intervention to the capabilities of its users and the realities of the care setting. It also helps explain why human interaction and clinician endorsement recurred so strongly as preferences; these supply the human and organisational fit that technology alone cannot.

## Future directions

Studies included in this review underrepresented or did not report on demographics related to certain groups. While findings revealed a difference in preference and engagement with digital content among people with low digital literacy, further research is needed to understand how preferences may vary among non-English speakers and individuals from lower-income populations. The use of established evaluation frameworks, such as RE-AIM, was rarely used by included studies, resulting in heterogeneity in the outcomes reported, including measures of patient preferences. Use of such frameworks will improve quality and scope of implementation data in future reviews. Few studies compared different delivery methods (e.g. websites versus apps), limiting the ability to generalise findings. Additionally, the sustainability of interventions or their long-term impact was rarely assessed, meaning the real-world benefits of interventions beyond the scope of research and evaluation contexts remain unclear. There is a need to investigate users’ ongoing engagement with digital models and their sustainability to ensure initial investment is not futile. Further, the current review did not compare cancer patients’ preferences between digital and non-digital approaches; the preferences and benefits of digitally delivered supportive care interventions can only be interpreted relative to other digital methods.

## Limitations

Several limitations should be considered when interpreting these findings. Most notably, the literature search was conducted up to July 2024, and studies published thereafter were not captured. Digital health is a rapidly evolving field, and recent developments, particularly artificial intelligence–based tools such as conversational agents and chatbots, may therefore be underrepresented in the included evidence. This limitation is tempered by the focus of the review: because we synthesised people’s preferences, perspectives, and requirements for digital supportive care rather than the effectiveness or technical features of specific technologies, the findings reflect relatively enduring prostate cancer care needs, such as preferences for tailored, plain-language content, human interaction, and accessible design, that are less susceptible to short-term technological change. The breadth of the included evidence (88 studies published between 1996 and 2024, spanning four consistent preference domains) further suggests that the identified domains are robust and unlikely to be materially altered by the addition of more recent studies. Nonetheless, given the pace of innovation, the preferences and barriers associated with emerging technologies warrant ongoing surveillance, and periodic updating of this evidence base will be important to ensure its continued relevance to contemporary digital cancer care.

## Conclusion

Digital models in prostate cancer care were acceptable and valued for their convenience, flexibility, immediacy of access, tailored content, clinician support, and interactive features, and facilitated self-management through decision-making, symptom management, and informational and psychosocial support. Interpreted through the HOT-fit framework, their value depended on the fit between the technology, the people using it, and the organisational context in which it was delivered, rather than on technological sophistication alone. Limited attention has been given to using digital platforms to support sexual wellbeing and recovery, which could offer a more accessible and comfortable way for people to engage with this sensitive area and represents an important priority for future development. Further research is also needed to understand how preferences vary among people with low digital literacy, non-English speakers, and lower-income populations and to evaluate the long-term engagement and sustainability of digital models so that initial investment is not futile.

## Supplementary Information

Below is the link to the electronic supplementary material.

ESM 1 DOCX (29.2 KB)

ESM 2 DOCX (428 KB)

## Data Availability

No datasets were generated or analysed during the current study.
