# Supplementary material for: Digital supportive care in prostate cancer: a systematic review
Source: Support Care Cancer. 2026 Oct 17;34(11):1117. doi: 10.1007/s00520-026-11192-x (PMC13676673; doi:10.1007/s00520-026-11192-x)
Supplement: Supplementary file 1 [file 520_2026_11192_MOESM1_ESM.docx]

Supplementary File 1. Database searches for the systematic review on the preferences of people with prostate cancer regarding digital supportive care.

| **Database:** APA PsycINFO via EBSCOhost | | | |
| --- | --- | --- | --- |
| **Date of search:** 23/07/2024 | | | |
| **Search #** | **Concept** | **Search Terms/Strategy** | **Results** |
| #1 | “Population” – Men affected by prostate cancer | PCa OR ((prostat*) N5 (cancer* OR carcinoma* OR neoplasm* OR tumo#r*)) | 8,590 |
| #2 | “Intervention” – Digital health | “artificial intelligence” OR AI OR digital OR electronic OR internet OR “mobile app*” OR online OR smartphone* OR technology OR telehealth OR telemedicine OR telephone OR video OR wearable* OR “web-based” | 619,330 |
| #3 | Supportive care | ((bereavement OR caregiver* OR cognitive OR communication OR “daily-living” OR decision* OR diet* OR employment OR emotion* OR “end-of-life” OR exercise OR existential OR family OR fear OR financial OR group OR health OR hospice OR “housekeeping” OR incontinence OR information* OR interperson* OR intima* OR isolat* OR lonel* OR model OR nutrition* OR pain OR “palliative care” OR partner OR “patient-clinician” OR “pelvic-floor” OR peer OR physical OR postoperative OR practical OR prehab* OR preoperative OR presurgical OR program* OR psychological OR psychosexual OR psychosocial OR sadness OR sexual OR social OR spiritual OR spous* OR terminal OR transition* OR uncertainty) N5 (care OR education OR need* OR support*)) | 979,664 |
| #4 | Outcomes of interest – acceptability and efficacy | acceptability OR adherence OR attendance OR attitude OR attrition OR awareness OR complian* OR complet* OR effectiv* OR efficacy OR enable* OR engagement OR experience OR facilitate* OR feasibility OR implement* OR motivat* OR opinion OR participat* OR perception OR preference OR retention OR success* OR uptake | 3,215,476 |
| #5 |  | #1 AND #2 AND #3 AND #4 | 495 |
|  | Peer-reviewed literature  Language: English | Limiters applied | 457 |

| **Database:** CINAHL via EBSCOhost | | | |
| --- | --- | --- | --- |
| **Date of search:** 23/07/2024 | | | |
| **Search #** | **Concept** | **Search Terms/Strategy** | **Results** |
| #1 | “Population” – Men affected by prostate cancer | (MH "Prostatic Neoplasms+") OR PCa OR ((prostat*) N5 (cancer* OR carcinoma* OR neoplasm* OR tumo#r*)) | 53,564 |
| #2 | “Intervention” – Digital health | (MH "Artificial Intelligence+") OR (MH "Digital Health+") OR (MH "Mobile Applications") OR (MH "Smartphone") OR "artificial intelligence" OR AI OR digital OR electronic OR internet OR “mobile app*” OR online OR smartphone* OR technology OR telehealth OR telemedicine OR telephone OR video OR wearable* OR “web-based” | 661,601 |
| #3 | Supportive care | ((bereavement OR caregiver* OR cognitive OR communication OR “daily-living” OR decision* OR diet* OR employment OR emotion* OR “end-of-life” OR exercise OR existential OR family OR fear OR financial OR group OR health OR hospice OR “housekeeping” OR incontinence OR information* OR interperson* OR intima* OR isolat* OR lonel* OR model OR nutrition* OR pain OR “palliative care” OR partner OR “patient-clinician” OR “pelvic-floor” OR peer OR physical OR postoperative OR practical OR prehab* OR preoperative OR presurgical OR program* OR psychological OR psychosexual OR psychosocial OR sadness OR sexual OR social OR spiritual OR spous* OR terminal OR transition* OR uncertainty) N5 (care OR education OR need* OR support*)) | 1,373,581 |
| #4 | Outcomes of interest – acceptability and efficacy | acceptability OR adherence OR attendance OR attitude OR attrition OR awareness OR complian* OR complet* OR effectiv* OR efficacy OR enable* OR engagement OR experience OR facilitate* OR feasibility OR implement* OR motivat* OR opinion OR participat* OR perception OR preference OR retention OR success* OR uptake | 2,560,336 |
| #5 |  | #1 AND #2 AND #3 AND #4 | 815 |
|  | Peer-reviewed literature  Language: English | Limiters applied | 767 |

| **Database:** Medline via EBSCOhost | | | |
| --- | --- | --- | --- |
| **Date of search:** 23/07/2024 | | | |
| **Search #** | **Concept** | **Search Terms/Strategy** | **Results** |
| #1 | “Population” – Men affected by prostate cancer | (MH "Prostatic Neoplasms+") OR PCa OR ((prostat*) N5 (cancer* OR carcinoma* OR neoplasm* OR tumo#r*)) | 262,902 |
| #2 | “Intervention” – Digital health | (MH "Artificial Intelligence+") OR (MH "Digital Health") OR (MH "Mobile Applications") OR (MH "Smartphone") OR (MH "Wearable Electronic Devices+") OR "artificial intelligence" OR AI OR digital OR electronic OR internet OR “mobile app*” OR online OR smartphone* OR technology OR telehealth OR telemedicine OR telephone OR video OR wearable* OR “web-based” | 6,071,908 |
| #3 | Supportive care | ((bereavement OR caregiver* OR cognitive OR communication OR “daily-living” OR decision* OR diet* OR employment OR emotion* OR “end-of-life” OR exercise OR existential OR family OR fear OR financial OR group OR health OR hospice OR “housekeeping” OR incontinence OR information* OR interperson* OR intima* OR isolat* OR lonel* OR model OR nutrition* OR pain OR “palliative care” OR partner OR “patient-clinician” OR “pelvic-floor” OR peer OR physical OR postoperative OR practical OR prehab* OR preoperative OR presurgical OR program* OR psychological OR psychosexual OR psychosocial OR sadness OR sexual OR social OR spiritual OR spous* OR terminal OR transition* OR uncertainty) N5 (care OR education OR need* OR support*)) | 2,761,199 |
| #4 | Outcomes of interest – acceptability and efficacy | acceptability OR adherence OR attendance OR attitude OR attrition OR awareness OR complian* OR complet* OR effectiv* OR efficacy OR enable* OR engagement OR experience OR facilitate* OR feasibility OR implement* OR motivat* OR opinion OR participat* OR perception OR preference OR retention OR success* OR uptake | 10,468,180 |
| #5 |  | #1 AND #2 AND #3 AND #4 | 3,452 |
|  | Peer-reviewed literature  Language: English | Limiters applied | 3,337 |

| **Database:** Scopus | | | |
| --- | --- | --- | --- |
| **Date of search:** 23/07/2024 | | | |
| **Search #** | **Concept** | **Search Terms/Strategy** | **Results** |
| #1 | “Population” – Men affected by prostate cancer | PCa OR ((prostat*) W/5 (cancer* OR carcinoma* OR neoplasm* OR tumor* OR tumour*)) | 425,141 |
| #2 | “Intervention” – Digital health | “artificial intelligence” OR AI OR digital OR electronic OR internet OR “mobile app*” OR online OR smartphone* OR technology OR telehealth OR telemedicine OR telephone OR video OR wearable* OR “web-based” | 9,735,161 |
| #3 | Supportive care | ((bereavement OR caregiver* OR cognitive OR communication OR “daily-living” OR decision* OR diet* OR employment OR emotion* OR “end-of-life” OR exercise OR existential OR family OR fear OR financial OR group OR health OR hospice OR “housekeeping” OR incontinence OR information* OR interperson* OR intima* OR isolat* OR lonel* OR model OR nutrition* OR pain OR “palliative care” OR partner OR “patient-clinician” OR “pelvic-floor” OR peer OR physical OR postoperative OR practical OR prehab* OR preoperative OR presurgical OR program* OR psychological OR psychosexual OR psychosocial OR sadness OR sexual OR social OR spiritual OR spous* OR terminal OR transition* OR uncertainty) W/5 (care OR education OR need* OR support*)) | 5,001,823 |
| #4 | Outcomes of interest – acceptability and efficacy | acceptability OR adherence OR attendance OR attitude OR attrition OR awareness OR complian* OR complet* OR effectiv* OR efficacy OR enable* OR engagement OR experience OR facilitate* OR feasibility OR implement* OR motivat* OR opinion OR participat* OR perception OR preference OR retention OR success* OR uptake | 27,492,231 |
| #5 |  | #1 AND #2 AND #3 AND #4 | 3,719 |
|  | Peer-reviewed literature  Language: English | Limiters applied | 2,398 |

| **Database:** Web of Science Core Collection | | | |
| --- | --- | --- | --- |
| **Date of search:** 23/07/2024 | | | |
| **Search #** | **Concept** | **Search Terms/Strategy** | **Results** |
| #1 | “Population” – Men affected by prostate cancer | PCa OR ((prostat*) NEAR/5 (cancer* OR carcinoma* OR neoplasm* OR tumor* OR tumour*)) | 386,153 |
| #2 | “Intervention” – Digital health | “artificial intelligence” OR AI OR digital OR electronic OR internet OR “mobile app*” OR online OR smartphone* OR technology OR telehealth OR telemedicine OR telephone OR video OR wearable* OR “web-based” | 5,772,338 |
| #3 | Supportive care | ((bereavement OR caregiver* OR cognitive OR communication OR “daily-living” OR decision* OR diet* OR employment OR emotion* OR “end-of-life” OR exercise OR existential OR family OR fear OR financial OR group OR health OR hospice OR “housekeeping” OR incontinence OR information* OR interperson* OR intima* OR isolat* OR lonel* OR model OR nutrition* OR pain OR “palliative care” OR partner OR “patient-clinician” OR “pelvic-floor” OR peer OR physical OR postoperative OR practical OR prehab* OR preoperative OR presurgical OR program* OR psychological OR psychosexual OR psychosocial OR sadness OR sexual OR social OR spiritual OR spous* OR terminal OR transition* OR uncertainty) NEAR/5 (care OR education OR need* OR support*)) | 2,382,080 |
| #4 | Outcomes of interest – acceptability and efficacy | acceptability OR adherence OR attendance OR attitude OR attrition OR awareness OR complian* OR complet* OR effectiv* OR efficacy OR enable* OR engagement OR experience OR facilitate* OR feasibility OR implement* OR motivat* OR opinion OR participat* OR perception OR preference OR retention OR success* OR uptake | 19,736,488 |
| #5 |  | #1 AND #2 AND #3 AND #4 | 1,911 |
|  | Peer-reviewed literature  Language: English | Limiters applied | 1,509 |
