# Supplementary material for: Digital supportive care in prostate cancer: a systematic review
Source: Support Care Cancer. 2026 Oct 17;34(11):1117. doi: 10.1007/s00520-026-11192-x (PMC13676673; doi:10.1007/s00520-026-11192-x)
Supplement: Supplementary file 2 [file 520_2026_11192_MOESM2_ESM.docx]

**Supplementary Table 2. Study Characteristics**

| **Study Characteristics**  (Author/ Code/  Country/  Design/  Aim) | **Population characteristics**  (Sample size/  Age/  Stage of ca/  Treatment) | **Intervention description**  (Supportive care type, Mode of delivery, Duration) | **Preference measure(s)** / scale(s) / assessment tool | **Findings /Outcomes (Re-AIM)** | | | | | | **Limitations** | **Implications/**  **Conclusions** |
| --- | --- | --- | --- | --- | --- | --- | --- | --- | --- | --- | --- |
|  |  |  |  | **Reported Preference (s)** | **Reach**  (Sample size, Participation %, Refusal % & reasons) | **Effectiveness** (QoL/ Positive/  negative effects /cost-  effectiveness) | **Adoption**  (Setting level/ provider or staff level) | **Implementation**  (Cost/  Identified enablers/  barriers) | **Maintenance**  (Org level /individual level / Follow up) |  |  |
| Badger et al 2013  **Country**: USA  **Design:** Randomised intervention  **Aim:** Examine which survivor characteristics moderate response to two interventions | **Population:** Men with PCa undergoing or completed treatment in past 6 months  **Sample:** N=71   - TIP-C (n=36) - THE (n=35)   **Age**: *M=*66.99 yrs  **Treatment:**   - Chemotherapy (14%) - RT (56%) - HT (42%) - Surgery (42%) - CAM (4%) - Monitoring (10%)   **Stage**:   - Stage 1 (15.5%) - Stage 2 (8.5%) - Stage 3 (11.3%) - Stage 4 (11.3%)   **Other**:   - White (85%) - ≥College (58%) - Retired (70%) - *M=*187 wks post-diagnosis. | **Intervention name:**   - Telephone Health Education (THE), vs. - Telephone Interpersonal Counselling (TIP-C)   **Supportive care type:**   - THE: Educational information focusing on prostate health & PCa; treatment, side effects & strategies; lifestyle interventions; & referrals & resources. - TIP-C: interpersonal psychotherapy & cancer education.   **Mode of delivery:** Phone  **Duration:**   - 8 weeks (one call/wk). - THE ~ 28 min/session - TIPC ~31 min/session | **Preference measures**: Participation  **Preference tools:** N/A  **QoL measures:**   - Centre for Epidemiological Studies-Depression Scale (CES-D) - Positive & Negative Affect Schedule (PANAS) - Perceived Stress Scale (PSS) - Perceived Social Support-Friend (PSS_FR) scale. - UCLA-PCI | High participation (93% T1-T2). | N=71  **Refusals:** N/R.  **Participation**: 93% (n=66/71) of enrolled participants completed intervention  **Dropout reasons:** N/A | **QoL:**   - THE more effective in reducing depression, negative affect, & stress. Effects pronounced in older men with lower prostate-function, social support & cancer knowledge. - TIP-C more effective in increasing positive affect, especially in men with higher education, prostate functioning, social support, & cancer knowledge.   **Positive effects:** N/A  **Negative effects:** N/A  **Cost-effectiveness:** N/A | **Setting:**   - Participants recruited from regional cancer & healthcare centres - Setting adoption N/R   **Provider/staff:**   - Trained interventionists: TIP-C: social worker (n=1) & nurse (n=1); THE: paraprofessional (n=2) - Staff adoption N/R | **Cost**:  N/A  **Enablers**:   - Educational PCa information for PCa developed by the National Cancer Institute - Interventions structured, standardised & manualised to ensure fidelity. - Sessions delivered weekly by trained staff.   **Barriers:**  N/A  **Adaptation:**  N/A | N/A | - Small sample size - Predominately white & educated sample - No control group - Potential self-selection bias - no comparison to other modalities | - Phone-based delivery increases accessibility. - Patient characteristic must be considered when recommending QoL interventions - Improving QOL in PCa may rely on addressing unmet informational needs before addressing psychosocial wellbeing |
| Belarmino et al 2019  **Country**: USA  **Design:** Non-randomised feasibility pilot  **Aim:** Examine feasibility & utilisation of mobile app to monitor & guide robotic assisted radical prostatectomy (RARP) recovery | **Population:** Men with PCa undergoing RARP  **Sample:** N=43   - participants (n=20) - non-participants (n=23)   **Age:**   - *M* = 63.53 yrs - Range = 47-75 yrs   **Stage: N/A**  **Treatment:** N/A  **Other:**   - Not Hispanic (65%) - White (75%) | **Intervention name**: N/A  **Supportive care type:**  App consisting of HRQOL instruments; push notifications to complete surveys, ambulate,  hydrate, & perform Kegel exercises (x3 per day); step-count tracking; & library of pre-& postoperative  instructions & Kegel instructions with diagrams.  **Mode of delivery:**  iPhone app.  **Duration/time**: 60 days | **Preference measures:**   - Satisfaction - Engagement   **Preference tools:**  Satisfaction survey  **QoL measures:** N/A | *Satisfaction*   - High satisfaction at day 30 (75%;): - Easy to use, understand & navigate (93%) - Reminders useful (100%) - recommend to others (93%) - More-developed app could replace in-person follow-ups (87%)   *Engagement:*   - 85% complied with push notifications to ambulate, hydrate, & perform Kegel exercises. - Better-than-average PRO response rates | N=43  **Refusal:** 53% (n=23/43) of people declined to enrol out of all who were approached  **Reasons**   - No iPhone (28%) - Did not speak English (9%) - Disinterested (9%) - Did not download app (7%)   **Participation:**  95% of participants accessed app at baseline  **Dropout reasons:** N/A | N/A | **Setting:**   - Urology Clinic - Pilot successfully implemented in clinical workflow   **Provider/ Staff:**   - Clinical research assistant approached participants to download app - Staff adoption N/R | **Cost: N/A**  **Enablers:**   - Integrated reminders & surveys - Easy onboarding (supported by staff) - HIPAA-compliant cloud storage   **Barriers:**   - Non-enrolment due to no iPhone. - Forgetting password   **Adaptation:**  87% interested in developed version to replace in-person follow-ups | N/A | - Low response rate - EPIC-CP captured but data not presented - Step count effectiveness N/A | - Overall high satisfaction with app. - Push notifications prompted ambulation, fluid intake, Kegel exercises, & survey completion. |
| Bender 2022  **Country:** Canada  **Design:** Single-arm pre-post pilot feasibility  **Aim:** Assess feasibility, acceptability & potential effects of peer navigation program among men with PCa & caregivers. | **Population:**   - Men with LPCa or stable metastatic PCa receiving treatment or follow up - Family caregivers   **Sample:** N=34   - patients (n=29) - caregivers (n=5)   **Age:**   - *M*= 65.2 yrs - Range= 48–78yrs   **Stage:**   - Stage I-II (88.2%) - Stage III-IV (8.8%) - Unsure (2.9%)   **Other:**   - White (88.2%) - Partnered (91.2%) - Heterosexual (91.2%) - University educated (66.8%) - Income ≥$100,000 (38.2%) - Unspecified (11.8%) | **Intervention name**:  True North Peer  Navigation (PN)  **Supportive care type:**  Peer navigation program including peer matching, peer navigator profiles, private messaging, health library of PCa resources and a case management dashboard for peer navigators  **Mode of delivery:**   - Online - Phone - FtF   **Duration:** 3 months | **Preference measures:**   - Feasibility (e.g., retention) - Acceptability/Satisfaction - Usage and usability   **Preference tools:**   - Feasibility (CHERRIES guideline for web-based survey, CONSORT for eHealth intervention - Acceptability (PSN-I for satisfaction, the 10-item System Usability Scale, terms of matching criteria, health library access, a 20-item measure perceived benefits)   **QoL measures:**   - Patient Activation Measure (PAM) - Health Quality of Life (EQ-5D-5L) - PC Quality of Life (PORPUS) - Hospital Anxiety & Depression Scale (HADS -A) (HADS-D) - Social Support (ESSI) - Support Care Needs Survey – Short Form (SCNS) | *Feasibility:*   - 78% registered to use the website. Of these, 93% matched with a peer navigator. - High engagement (600 messages, 157 interaction logs) - Matching rate: 92.7%   *Acceptability*:   - High satisfaction with Peer Navigator relationship, peer matching process, app registration, support received (>74%) - Moderate satisfaction with program & interactions with program staff (>66%)   *Usability:*   - Preferred to be matched with navigator who shared characteristics (71.4%) than any navigator (28.9%) - Health library accessed 22 times - Participants matched with navigator accessed at least 1 health library resource | N=34  **Refusal:**   - 42% of approached individuals (n=50 patients, n=11 caregivers) declined to participate:   **Reasons:**   - Lack of perceived need - Feeling they were adequately supported   **Participation**:   - 78% registered used website   **Dropout reasons:**   - lost to follow-up - overwhelmed - not needing support. | **QoL:**   - Significantly increased PCa-specific QoL - Significantly decreased fear of recurrence   **Positive effects:**   - Significant improvements in informational support,   **Negative effects:** N/A  **Cost-effectiveness:** N/A | **Setting:**   - cancer centres (n=2) - Integrated into supportive care offerings   **Provider/staff:**   - Primary support providers (peer navigators) and secondary healthcare support/supervision (oncology nurses, urologists, radiation oncologists, psychologists) - Limited support for peer navigators; staff availability scored low (3.4–3.7/10) | **Cost:** N/A  **Enablers:**   - Personalized matching - 6-week peer navigator training and peer navigator supervision and support. - Integration of theory-based design (Stress & Coping, Social Cognitive, Optimal Matching)   **Barriers:**   - lack of perceived need for support impacted enrolment - Number of steps in the registration & matching process, necessitating a streamlined approach. - Technology proficiency impacted registration/navigation - Low engagement with health library   **Adaptation:**  Potential integration into standard cancer care services. | N/A | - Small sample - Single-arm design; no control group - Sample predominantly white, educated & literate - Missing data for some QoL items - Limited generalisability | - Participants valued interpersonal connection & validation through peer navigation, especially when matched on preferred criteria. - Program & app satisfaction were moderate. - Participants highly satisfied with support received from peer navigator & reported several benefits. - most important matching criterion was spoken language, treatment received & sexual orientation. |
| Berry et al 2013  **Country:** USA  **Design:** Prospective RCT  **Aim:** Compare usual patient education plus the internet-based P3P, vs. usual education on conflict associated with treatment-related decision making. | **Population:** Men with newly diagnosed LPCa not yet on therapy who are candidates for at least 2 treatment options.  **Sample size:** N=494   - Intervention (n=266) - Control (n=228)   **Age:**   - Intervention median 62 yrs (range 40-84 yrs) - Control median 63 yrs (range 45-86 yrs)   **Stage:** Localised (T1 or T2)  **Treatment:** Pre-treatment  **Other:**   - ≥College (56.8%) - Married/partnered (77.1%) - ≥4 weeks since biopsy (66.6%) - Treatment preference (51.1%) - Undecided about treatment (48%) | Intervention name:   - Personal Patient Profile-Prostate (P3P), vs - Education (usual care)   **Supportive care type:**  Decision support system  **Mode of delivery:**   - Touch-screen computers in clinic waiting rooms - web-based access at home   **Duration:** 6 months | **Preference measures:**   - Acceptability - Satisfaction - Acceptability - Usefulness - Use   **Preference tools:**   - Acceptability E-scale.   **QoL measures:**   - Spielberger State-Trait Anxiety Inventory (STAI) - Expanded Prostate Index Composite (EPIC-26) - The Decisional Conflict Scale (DCS) | - Acceptability E-Scale *M*: 25.1/30 - High satisfaction with teaching on statistics & external website links - Usefulness of content: *M* ≥ 3.7/5   *Useability*   - 69% accessed P3P at home; 88% follow | N=494  **Refusal:**   - 27% (n=228/841) approached declined participation.   **Reasons**: N/A.  **Participation**: 88% follow up at 1 month and 6 months  **Dropout reasons: N/A** | **QoL:**   - significant reductions in 2 decisional conflict subscales (uncertainty & values clarity) - No change to remaining subscales - Undecided men in intervention group started treatment sooner (not statistically significant)   **Positive effects:**  Useful links to access external resources  **Negative effects:**  N/A  **Cost-effectiveness:**  N/A | **Setting:**   - Consent and questionnaire completed in 6 clinic waiting rooms - P3P successfully integrated into clinic workflow at 6 clinics across 4 cities   **Provider/staff** :   - Research assistants provided in-clinic support - Automated system required little staff input; no training needed | **Cost:** N/A  **Enablers:**   - Online access offered flexibility & potentially increased LPC engagement with the P3P - text & video intervention tailored to personal factors (e.g. demographics)   **Barrier:**  Limited internet access  **Adaptation:**  Intervention was automated, requiring minimal provider time or effort. | Setting level:  N/A  **Individual level**:  Reduced decisional at 6 months | - No stratification in randomization - Limited generalisability - Some participants had already formed treatment preferences, limiting intervention impact - Short-term evaluation; long-term decision regret & QoL N/A | P3P significantly reduced decisional conflict related to uncertainty & personal values   - Highly rated personalisation, web-delivery, acceptability & usefulness of program. |
| Beydoun 2014  **Country**: Australia  **Design**: Three-arm non-RCT  **Aim**:  Assess combination of education, short-term aerobic & resistance exercise on ADT treatment | **Population:** Men with relapsed or metastatic PCa on leuprorelin acetate  **Sample size:** N=859   - FtF exercise ( n=379) - At Home exercise (n=255) - Man Plan Support (n=208)   **Age:** *M=* 71± 8 yrs  **Treatment:** ADT  **Stage:** Relapsed or metastatic | **Intervention name**   - FtF: Supervised group exercise sessions & home-based exercise program, vs - At Home: Home-based exercise program, vs - Man Plan: Education on diet, exercise, & psychosexual function.   **Supportive care type:**  Aerobic & resistance exercise program**.**  **Mode of delivery:**   - FtF: In person, phone, magazines - At Home & Man Plan: phone, magazine   **Duration:**   - F2F: 10-weeks (2 groups /week), followed by 6-month home exercise - At Home: 6 months - Man Plan: N/A. | **Preference measures:**   - Satisfaction - Program interest - Motivation   **Preference tools:** Satisfaction phone survey  **QoL measures: N/A** | *Note: FtF reported below*   - Enjoyment of exercise sessions: 100% - Motivation to return to sessions: 86% - Interest in continuing with program & receiving quarterly magazines: 100% | N=859.  **Refusal:** None.  **Participation:**   - Overall: 91% completed program. - F2F (96%) - At Home (88%) - Man Plan (87%)   **Drop-out reasons:**   - discontinuation of ADT (n = 52) - no longer wishing to participate (n=8) - deceased (n=8) - *M* time in program withdrawal was 147 days. | Note: FtF reported below  **Qol**   - Improved wellbeing (91%) - Significant improvement in cardiorespiratory fitness & strength at 10 weeks. - **Positive effect** group exercise motivating (86%). - positive impact on overall fitness at T1 & T3 (98%); 6 months (87%) - exercise increased fitness: 98% - starter home exercise kit useful (77%) - Interest in joining At Home program (76%) - improvements in physical & emotional wellbeing by 3 months   **Negative effect:** N/A  **Cost-effectiveness:** N/A | **Setting:**   - Participants recruited from several institutions (details N/R) - Community-based, coordinated centrally, conducted in clinics & homes. - Effective in multiple institutions; high engagement across models   **Provider/ staff:**   - Accredited exercise physiologist health coaches - Staff adoption N/R | **Cost:**  N/A  **Enabler**   - 2-yr ongoing support (phone & magazines) yrs - Structured program, health coach support, educational materials - F2F: asked only to complete intensity of - exercise comfortable to them. - Group sessions provide a socially stimulating & motivating environment. - majority found home kit (manual & DVD) beneficial (77%) - Allowed patients to exercise in comfort of their own home   **Barriers**:  Medical conditions precluding physical activity  **Adaptation:**  Exercise programs individualised based on patient capabilities & preferences. | **Setting level:**   - Continued support through newsletters & phone lines.   **Individual level:**   - Plans to continue exercising(97%), continue program & receive magazines (100%) - improved physical (93%) & emotional wellbeing (79%) at 6 months | - Insufficient information on validity of measurement tools - Low response rate - Group allocation based on patient preference, medical comorbidities & fitness level. - Patients with musculoskeletal, cardiovascular or neurological disorders precluding them from exercise were enrolled into support program. - Limited completion of follow-up surveys. - Limited demographic data reported. - Differences in measures across groups precludes direct between-group comparison | - short-term structured exercise intervention resulted in high compliance & significant improvements in muscle strength & fitness in PCa patients on ADT. - Emotional & physical wellbeing improvements sustained at 6 months for majority |
| Bjoernes 2012  **Country:** Denmark  **Design:** Qualitative  **Aim:** Explore how a dialogue-based web application contributes to the accessibility of HCP & information exchange for men undergoing PCa treatment. | **Population:** Men with PCa planning for prostatectomy  **Sample**: N=34  **Age**: N/A  **Stage**: N/A  **Treatent**: RP | **Intervention/tool name**:  The Online  Patient Book©  **Supportive care type:**  Dialogue-based web app that enhances personalised access to HCP.  **Mode of delivery:** Online website, with open access (public) area & login (personal) area  **Duration:** Throughout treatment | **Preference measures**:   - Perspective/ experience - Acceptability & usability   **Preference tools:**   - Web-based survey with open-ended questions. - Hermeneutics qualitative interview (open-ended interview)   **QoL measures:** N/A | - Improved accessibility to & communication with HCP - application a "lifeline" to timely answers within 24 hours. - Preference for written communication to formulate thoughts without time pressure. | **N=34**  **Refusal: N/A**  **Participation:**  59% (n=34/58) of potential evaluators completed written responses.  **Dropout reasons: N/A** | **Qol:**   - Increased feelings of security & empowerment, & reduced anxiety   **Positive effects:**   - Enhanced accessibility to HCP   **Negative effects:** N/A  **Cost-effectiveness:** N/A | **Setting:**   - Clinical practice of a urology department - intervention incorporated into patient care workflow   **Provider/ staff:**   - Clinical nurses enrolled patients into system - HCP monitored and answered patients' queries 24/7 - HCP adapted schedules to allocate time for answering online queries alongside in-person duties | **Cost:**  N/A  **Enabler:**   - Flexible & easy to access - Accessible 24 hours - Secure & fast response from HCP   **Barriers:**  N/A  **Adaptation:**  N/A | **Setting level:**  Integrated into standard patient care  **Individual level:**  Patients were allowed to evaluate system from 2-7 months post-surgery. | - No long-term follow-up data - Lack of demographic information | - High patient satisfaction & positive feedback. - dialogue-based web application improved enhanced accessibility & provided secure platform for communication with HCP. - Patients valued online contacts, experienced feelings of partnership, & appreciated the flexibility & calm environment. They felt empowered & secure, with the ability to be active partners in their care. - Engagement with Q&A portion of website poorly utilised. |
| Bouchard 2019  **Country:** USA  **Design:** RCT  **Aim:** Examine the acceptability & efficacy of a tablet-delivered psychosocial intervention for older men with advanced PCa | **Population:** Men diagnosed with advanced PCa who received ADT  **Sample size**: N=192   - CBSM (n=95) - HP (n=97)   **Age:** *M=*68.8 yrs  **Stages:** III-IV  **Treatment:** ADT  **Other**:   - Non-Hispanic White (56.8%) - Black (37.5%) - ≥College (68.1%) - Married/partnered (67.1%) - Retired (56.5%) - Income ≤ $35,000 (38.6%) | **Intervention name**:   - Cognitive-behavioural stress management (CBSM) vs - health promotion (HP) control   **Supportive care type:**  Psychosocial intervention focused on CBSM & HP.  **Mode of delivery:**  Tablet-based, web-delivered group sessions  **Duration**: 10 weekly sessions   - CBSM: 90 min (30 min relaxation; 60 min discussion/stress management) - HP: 60 min (healthy lifestyle information) | **Preference measures:**   - Engagement - Acceptability   **Tools:**   - satisfaction surveys - Retention & attendance rates - Weekly evaluation & post-program surveys   **QoL Tools:**   - 18-item Memorial Anxiety Scale for PC (MAX-PC) | *Engagement*   - both CBSM & HP acceptable (>70%) - Attendance (≥70%) - Two thirds (67%) of participants attended eight or more sessions.   *Acceptability*   - Favourable in both CBSM & HP - Significant greater acceptability of all CSBS & HP components in black men - In HP, black men (vs. NHW men) rated the weekly information as more useful & reported greater acceptability of all HP components. - In CBSM, black men reported significantly greater acceptability of all CBSM components. | N=192  **Refusal**:   - 5% (n=258/4824) of individuals screened declined participation or were unable to be contacted. - 5% (194/4824) of individuals screened enrolled in study - most (4372/4630) ineligible.   **Participation**   - Overall: 89% completed group allocation. - CBSM: 88% completed intervention. HP Control: 87% (completed   **Dropout reasons:** N/A | **QoL:**   - Reduction in PCa anxiety in both groups at 6 mo, greater in CBSM, but not sustained at 12 months for either group - Black men in CBSM reported significant reduction in PC-anxiety at T2, maintained at 12 months.   **Positive effects**: N/A  **Negative effects:** N/A  **Cost-effectiveness:**  N/A | **Setting:**   - medical centres (n=2) & veterans’ affairs medical centre (n=1) - Setting adoption N/R   **Provider/staff:**   - Trained therapists facilitated intervention via video conferencing - Psychologists supervised fidelity of the intervention - Staff adoption N/R | **Cost:**  N/A  **Enablers:**   - Flexible scheduling & transportation support to increase minority participation - Participant training for using platform & tablet - Providing tablets as part of study - Participants could join sessions via phone if had connectivity issues with tablets.   **Barriers:**   - Reliant on tablets & internet access - Potential technology access issues outside the study - Lower socioeconomic status among Black participants may affect future accessibility. | **Setting level:**  N/A  **Individual level:** Anxiety reduction among black men in CBSM was maintained at 12 months | - No long-term assessment beyond 12 months - Prior knowledge of stress management techniques not assessed. - Program relies on tablets | - Tablet-delivered CBSM was feasible, acceptable, & effective in reducing disease-specific distress, particularly among Black men. |
| Chambers 2015  **Country:** Australia  **Design:**  Three-arm pilot RCT  **Aim:** Evaluate efficacy of couples-based peer-vs nurse delivered phone support vs usual care in improving sexual & psychosocial adjustment among men with LPCa & female partners | **Population:** Heterosexual couples where male was diagnosed with LPCa  **Sample size:** N=189 dyads   - Peer intervention (n=63) - Nurse intervention (n=62) - Usual care (n=64)   **Age:**   - Men *M=* 62.7 yrs (SD=6.80) - Women *M=*59.8 yrs (SD=7.38)   **Stage:** LPCa  **Treatment:**   - RP in past 12 months - Time since diagnosis *M=*127.6 days (SD=146.8) - Median Gleason score=7   **Other:**   - Relationship duration *M=*32.5 yrs (SD=11.8) - Tertiary educated (Men 65.1%, Women 47.6%) - Full-time employment (Men 42.3%, Women 25.9%) - Income >$60,000 (53.4%) | **Intervention name**: N/A   - Supportive care type: Couples-based psychosexual support. Three arms: - Peer-support: trained PCa survivors (≥12 months post-treatment) - Nurse-counselling: experienced PCa nurses with CBT principles - Usual care: Standard medical management with patient education   **Mode of delivery:**  Phone  **Duration:**   - 7 sessions for pre-surgery - 6 sessions for post-surgery - Peer group M=29.07 min/session - Nurse group M=36.46 min/session | **Preference measure:**   - Engagement   **Preference tools:**   - Helpfulness rank of telephone calls - Open-ended questions for success of program/feedback   **QoL measures:**   - International Index of Erectile Function (IIEF) - Female Sexual Function Index (FSFI) - Supportive Care Needs Survey (Sexuality Needs subscale) - Psychological Impact of Erectile Dysfunction—Sexual Experience - Masculine Self-Esteem Scale - Revised Dyadic Adjustment Scale - Miller Social Intimacy Scale | - Participants rated phone support from nurses higher in helpfulness & therapeutic alliance compared to peers. - Peer support valued for shared experience & empathy - nurse support appreciated for professional advice & patient education | *Note: Reported for dyads*  N=189  **Refusal rate**:   - 75% (588/782) of referred individuals did not participate (included ineligible & refusal). - 53% (216/405) of eligible couples declined participation. - Refusal reasons included not needing support, not interested, not completing questionnaire, & being too busy.   Participation (at 6-mo):   - Overall 92% (n=173/189) - Usual care:92% (n=59/64) - Nurse intervention: 92% (n=57/62) - Peer intervention: 90% (n=57/63).   **Dropout reasons:** Primarily lack of interest. | **Qol:**  No significant improvement in sexual function, sexual self-confidence, masculine self-esteem, marital satisfaction, or intimacy  **Positive effects:**  Increased use of medical treatments for erectile dysfunction (ED) in intervention groups at 12 months  **Negative effects:**  N/A  **Cost-effectiveness:**  N/A | **Setting level:**   - Telephone intervention delivered from multiple private and public hospitals - structure was feasible for community-wide implementation by avoiding need for FtF delivery in clinical setting   **Provider/ staff:**   - CNS counsellors (n=2) delivered nurse intervention; Psychologist (n=10) provided clinical supervision on intervention; Trained volunteer peers delivered peer support - High protocol adherence and effective training | **Cost:** N/A  **Enablers:**   - phone delivery enhanced accessibility - Strong therapeutic alliance (especially in nurse group)   **Barriers:**   - Desire for earlier support & better matching with support providers - Preference for FtF interactions in some participants | **Setting level:**  N/A  **Individual level:**  High adherence during the 12-month study period | - Not generalizable to same-sex couples - Possible recruitment bias: couples most in need may not have participated | - Peer & nurse-delivered phone interventions increased the uptake of medical treatments for ED, but did not translate into improved sexual or relationship outcomes. - Both support models were valued but offered different types of preferred support. |
| Chan 2020  **Country**: USA  **Design:** Four-arm pilot RCT  **Aim**: Evaluate feasibility, acceptability & change in diet & physical activities after online exercise & diet educational information program | **Population:** Men with PCa  **Sample size**: N=202   - Level 1 (n=49) - Level 2 (n=51) - Level 3 (n=50) - Level 4 (n=52)   **Age:** Median=70 yrs  **Stage:**   - I (30.7%) - II (43.1%) - III (13.9%) - IV (5.9%;) - Unknown (5.9%)   **Treatment:** Completed treatment for localised disease & PSA low or undetectable (53%)  **Other:**   - White (92.6%;) - ≥College (82.7%) - Married/partnered (91.6%) - Retired (62.4%) | **Intervention name**:  TrueNTH Community  of Wellness  **Supportive care type:**  Web-based diet & physical activity with 4 arms:   - L1: Website with educational materials - L2: Personalized diet & exercise prescription via website - L3: L2 + Fitbit device + supportive text messages - L4: L3 + 2x30-minute coaching calls (exercise trainer & dietician)   **Mode of delivery:** Online  **Duration:** 3 months | **Preferences measures:**   - Feasibility - Acceptability   **Preference tools:**   - Web-based acceptability survey & website usage analytics   **QoL measures:**   - Community Health Activities Model Program for Seniors (CHAMPS) survey - Validated food frequency questionnaire (FFQ) | *Feasibility*   - Retention at   3-month (82.7%), 6-month (77.2%)  *Acceptability*   - Community of wellness intervention: moderate satisfaction across all arms (>40%), L2 highest - Resource directory: low use, non-usage highest in L3 (41%) - Exercise prescription: mostly “good” - Diet prescription: mostly rated “good” to “excellent” - Weekly progress report mostly rated “good” to “very good” - Fitbit: mixed use- 20% of L3 rated “excellent”; 29% of L4 “did not use” - text message (Good: L3=32%; Did not use; L4=29%) - Exercise coach: rated positively in L4 by 42% | **Sample Size:** N= 202  Note low response rate: 5% (292/6406) of individuals who were contacted responded.  **Refusal Rate & reasons:**   - 8% (n=2/292) who responded to recruitment refused or failed to consent. - Reasons included: failing to consent, not interested, not tech savvy, & health reasons.   **Participation Rate:**  Overall, 79% (n=160/202) who were randomised were exposed to the intervention over 3 months.   - Level 1 97% (38/49) - Level 2 75% (38/51) - Level 3 84% (42/50) - Level 4 81% (42/52)   **Dropout reasons**: primary reason was time commitment | **Qol:**  N/A  **Positive effects:**   - Small improvement in diet score at L4 - increased physical activity in less active participants at baseline.   **Negative effects:**  Minor adverse events (AEs) like muscle pain & fatigue increased with intervention intensity but were mild/moderate.  **Cost -effectiveness:**  N/A | **Setting:**   - academic centres (n=3). - Required no changes to existing hospital infrastructure.   **Provider/staff:**   - Registered dietitian (n=1) and exercise delivered coaching for participants at intervention level 4 - Staff interaction minimal, but those who received coach calls reported higher satisfaction and engagement | **Success Rate:** Surveys completed at 3-month follow-up (82.7%; n=167/202)  Surveys completed at 6-month follow-up (77.2%; n=156/202) | N/A | - Sample predominately white & educated - Use of a single physical activity tracker was mentioned in exit surveys, that participants would prefer options for devices they already owned to allow easier use in current lifestyle | - Men who received tailored online information, wearable and coaching calls had higher intervention engagement and satisfaction, and reported diet and physical improvements in diet and physical activity more than those who received access to general online information. |
| Chien 2020    **Country:** Taiwan  **Design:** Quasi-experimental  **Aim:** Develop  & examine effectiveness of a couple-based psychosocial information  packet & multimedia psychosocial information | **Population:** Newly diagnosed PCa patients & intimate partners  **Sample size:**   - N=103 dyads - Control (n=50 dyads) - PIP (n=25) - MPI (n=28)   **Age:** Patients *M=*67.8 yrs  **Stage:**   - Early-stage T2N0M0 (69.9%) - T3N0M0 (30.1%)   **Treatment:** RP or RT  **Others:**   - ≥College (45.6%) - Employed (40.8%). - Religious (78.6%) - Relationship duration *M*=39.7 yrs (SD = 9.7). | **Intervention name**:   - Psychosocial Information Package (PIP), vs - Multimedia Psychosocial Intervention (MPI)   **Supportive care type:**  Couple-based aimed at improving disease appraisal, emotion status, relationship satisfaction, & HRQOL  **Mode of delivery:**  PIP: Mail & phone counselling  MPI: Education films sent via mobile messaging application, information manual & professional support via message or phone  **Duration:** 6 weeks (1/week) | **Preference measures**:   - Satisfaction   **Preference tools**:   - Modified questionnaires   **QoL Measures:**   - Short-Form Health Survey (SF-12) - MAX-PC - Effectiveness in emotional status (Positive & Negative Affect Schedules – PANAS) | - Patients satisfied with the multimedia information & believed information was useful - Intended to recommend information to others - Satisfaction with MPI: *M=*8.63 (*SD* = 1.46) & *M*=8.91 (*SD* = 1.40). | N=103 dyads  **Refusal rate:**   - 12% (n=60/521) of individuals assessed for eligibility declined to participate; - 67% (n=351/521) were ineligible. - 61% (n=103/170) of eligible participants were allocated to groups - Reasons N/A   **Participation**:   - Overall: 99% completed - Control Group: 100% completed - PIP Group: 96% completed - MPI Group: 100% completed   **Dropout reasons:** N/A | **QoL:**  No significant improvements | **Setting:**   - urology outpatient departments or wards in hospitals (n=3) - Minimal structural adaptation required. Technology (smartphone apps) was leveraged to avoid major setting-based disruptions.   **Provider/staff:**   - Urology-trained nurses delivered scripted support by phone/app; received training in psychosocial support and digital delivery. - Nurses adapted to a hybrid digital-coaching model, requiring moderate training, technological fluency, and emotional support skills | **Cost:**  N/A  **Enablers:**   - Multimedia delivery increased engagement - Telephone support provided personalised assistance.   **Barriers:**   - Challenge in Scheduling telephone counselling. - Potential issues with technology access for some participants. | N/A | - Relatively small sample size, and non-equivalent group sizes - Only early-stage PCa patients (who had radical prostatectomy and radiotherapy) and their partners – could not extrapolate to patients with advanced PCa or patients with alternate treatments - Participants or partner had to use a smartphone for program – therefore can’t apply success for people without a smartphone | - Partners of PCa patients in PIP group saw significant improvement in mental HRQOL - Future study requires more rigorous and larger sample sizes to aid in showing effectiveness of intervention |
| Cockle-Hearne 2018  **Country:** UK  **Design:** Mixed method  **Aim:** Assess feasibility of web-based CBT program for reducing distress after PCa treatment | **Population:** Men experiencing distress after PCa treatment  **Sample size:** N=135   - Phase 1 (n=61) - Phase 2 (n=74)   **Age:**   - Phase 1 Median 68 yrs - Phase 2 Median 62 yrs   **Stage:** I-IV  **Treatment:**   - Prostatectomy - RT - brachytherapy - HT | **Intervention name**: N/A  **Supportive care type:**  Web-based, self-guided CBT program with filmed peer support & interactive chat rooms  **Mode of delivery:**  Online  **Duration:**  1 hour/week for 4-weeks | **Preference measures**   - Uptake - Adherence - Satisfaction   **Preference tools:**   - Participation Satisfaction Questionnaire   **QoL measures:**   - Patient Health Questionnaire-9 (PHQ-9) - General Anxiety Disorder Scale- 7 item (GAD-7) - General health Questionnaire-28 (GHQ-28) - SESCI | *Uptake & Adherence*   - High adherence and low attrition - Low initial uptake (12–14%).   *Satisfaction:*   - High acceptability of the program - High satisfaction with peer support videos & chat forum - The use of the devices was comfortable, effortless, flexible - link provided direct access to search for appropriate information - chat room was a safe environment to share truthful information - Facilitator involvement improved chat room participation | **Sample size:**  N=135  **Refusal:**  87% (903/1038) of invited men did not enrol or ineligible.   - Phase 1: 86% (371/432) of invited men did not enrol or were not eligible. - Phase 2: 88% (532/606) of invited men did not enrol or were not eligible - Refusal reasons: N/A.   **Participation:**   - Phase 1: 80% (8/10) of participants started & completed the intervention. - Phase 2: 80% (16/20) of participants started & completed the intervention.   **Dropout reasons:** bereavement or not provided | **QoL**   - Reduction in distress score for both phases after - Reduction in anxiety & somatic symptoms. - Improved coping   **Positive effects:** N/A  **Negative effects:** N/A  **Cost-effectiveness:**  N/A | **Setting:**   - low-intensity psychological support service in primary care (Phase I) or a secondary care cancer service (Phase II) - no service disruption; risk protocols adapted from IAPT; CMS redevelopment improved usability - Successfully integrated into clinical practice in both settings.   **Provider/staff:**   - Psychological practitioners (trained via IAPT framework, Phase I) or a CNS (Phase II) - Some difficulty in shifting from traditional support role to facilitative, self-management role. | **Retention rate:**   - 80% of participants in both phases remained in the 4-week intervention & completed all assessments   **Enablers:**   - Strong engagement with peer support & chat rooms. - Accessibility of web-based intervention.   **Barriers:** stigma around psychological support. | N/A | - Small sample size limits generalizability. - Lack of control group - No long-term follow-up to assess the sustainability of effects. - No cost effectiveness analysis - Few men enrolled or eligible to participate - Inconsistent module length | - web-based intervention successfully integrated into both primary & secondary care settings. - formal cost-effectiveness analysis is still required |
| Crafoord 2020  **Country**: Sweden  **Design:**  Mixed method  **Aim**: Describe engagement with interactive symptom self-management app | **Population:** Patients with locally advanced PCa undergoing RT  **Sample size**: N=75  **Age:** Median 72 yrs (range 44-81 yrs)  **Stage:**   - T1-T3 - Gleason score 6-9   **Treatment:** RT antihormonal treatment (73%)  **Other:**   - Married/partnered (78%) - University (42%) - Retired (68%) | **Intervention name**:  Interaktor  **Supportive care type:**  Symptom monitoring & self-care  **Mode of delivery:**  Smartphone or tablet app  **Duration:** Daily from first day of RT until 3 weeks after treatment (approximately 9 weeks) | **Preference measures:**   - Adherence - Satisfaction   **Preference tools:**   - Usage - Qualitative interviews   **QoL measures:**  N/A | *Adherence:*   - high adherence (83%) - Triggered at least one alert: 72% - Viewed at least one self-care advice: 87% - Free text function used: 75% (n=56/75)   *Satisfaction:*   - Patients found app was user friendly, easy to use & convenient - Daily reporting was meaningful, particularly at start of treatment - HCP interactions created feelings of support - Self-care advice valuable, applicable & informative - Requests for additional psychological & dietary advice | **Sample size**: n=75  **Refusal**: N/A  **Participation**: All participants completed intervention. | **Qol:**  N/A  **Positive effects:**   - Increased patient engagement in symptom management - Provided reassurance & continuous contact with HCP - Facilitated self-care & symptom awareness   **Negative effects:**  Some patients found symptom reporting to be a stressful reminder of illness  **Cost-effectiveness:**  N/A | **Setting:**   - University hospitals (n=2) - intervention well-integrated into standard outpatient workflows using digital augmentation   **Provider/staff:**   - Oncology nurses monitored symptom reports & responded to alerts - Staff adoption efficient with minimal training and moderate workload impact. Nurses integrated app-based alert response into their daily workflow without disrupting existing services. | **Implementation:**  Adherence rate: 83%; dropped at day 39  Reporting period:  54- 89 days (range)  **Cost:**  N/A  **Enablers**   - Easy-to-use interface - Automated symptom alerts & nurse response system - Reminder notifications   **Barriers:**   - Some patients had difficulty remembering to report symptoms - Technical issues for some (e.g., graph readability, lack of familiarity with apps)   **Adaptations:** | **Setting level:**  N/A  **Individual level:**  Some patients wanted to continue using the app post-treatment for symptom tracking | - Differences in app versions between patient groups No logging of interactions with links or symptom history graphs - Some patients did not recall details about their app usage during interviews - Potential social desirability bias in interview responses | - app was valued for continuous healthcare contact & symptom management. |
| Crafoord 2023  **Country:** Sweden  **Design:**  Two-arm RCT  **Aim:**  Evaluate patients' perceptions of individualized care & health literacy using interactive app during curative treatment for BCa & PCa | **Population:**  Patients undergoing curative treatment for non-metastatic BCa or PCa  **Sample size:** N=150   - Intervention (n= 75) - Control (n= 75)   **Age:** *M=* 70.1 yrs  **Stage:** Non-metastatic  **Treatment:**  External RT  **Other:**   - university (40%) - Married/cohabiting (73.9%) - Employed full time (30.1%) | **Intervention name**:  Interaktor  **Supportive care type:**  Symptom monitoring, self-care & HCP follow up alerts  **Mode of delivery:**  Smartphone or tablet app  **Duration:**  Daily from first day of RT until 3 weeks after treatment (approximately 9 weeks) | **Preference measures**:   - Perceived Support regarding care: ICS-A, ICS-B   **Preference tools:**   - Individualised Care Scale (specific nursing interventions - ISC-A & individuality in the care delivered – ISC-B)   **QoL measures**: N/A | Higher perceived individualised care in decision control | **Sample size**: N=150 (n=75 intervention, n=75 control)    **Refusal rate**:   - 55% (193/349) declined to participate (excluding n=6 ineligibilities). - 43% (150/349) of eligible participants consented to participate.   **Refusal reasons**:  N/A  **Participation**: 100% | **Qol**  N/A  **Positive effects:**   - Improved decision control & health literacy - Stable perceptions of individualised care   **Negative effects:**  N/A  **Cost-effectiveness:**  N/A | **Setting:**   - Oncology clinics (n=2) - Integration in routine cancer care, specifically during neoadjuvant chemotherapy (BCa) and radiotherapy (PCa).   **Provider/staff:**   - Registered oncology nurses managed app alerts and contacted patients - intervention absorbed into routine daily tasks, requiring no new staff or major time reallocation. | **Implementation:**  High adherence rate  Completion/success rate:79 % (n=58/73)  **Cost:** N/A  **Enablers:**  Real-time symptom tracking & alerts facilitated timely healthcare provider intervention   - Education level significantly influenced health literacy outcomes.   **Barriers:**   - Challenges in older participants using digital tools - No analysis of app usability or technical difficulties | **N/A** | No cost-effectiveness analysis. | An interactive app improved decision control & health literacy in PCa patients |
| Cuypers 2018  **Country:**  The Netherlands  **Design:** Pragmatic cluster RCT  **Aim:**  Compare patients’ evaluation of treatment decision-making in LPCa between standard counselling vs counselling + online DA. | **Population:**  Patients newly diagnosed with LPCa who had at least two treatment options.  **Sample size:** N=336   - DA (n=235) - Control (n=101)   **Age:**   - DA *M=*64.9 yrs - Control *M=*66.3 yrs   **Stage:** LPCa  **Treatment:** N/A (new diagnosis)  **Other:**   - Married/partnered (87%) - Highly educated (36%–44%), - Gleason Score 6 (DA=61%; Control=69%) | **Intervention name**: N/A  **Supportive care type:**  A web-based PCa DA with values clarification exercises (VCEs) to support informed treatment choices.  **Mode of Delivery:** Online  **Duration:**  Before treatment decision-making | **Preferences measures:**   - Preparation - Satisfaction   **Preference tools:**   - Preparation for Decision-making Scale (PDMS) - PCa Decision Quality Instrument (PCDQI) - Satisfaction with Cancer Information Profile (SCIP-B)   **QoL measures:** Hospital Anxiety & Depression Scale (HADS) | - Lower satisfaction with information provision was reported in the DA group compared to the control group - Some patients preferred a paper-based version of the DA rather than an online format. | **Sample Size:** N=336  **Refusal Rate:**  21% (102/484) of invited participants refused participation.  **Refusal reason: N/A**  **Participation Rate:**   - Overall: 88% (n=336/382) of allocated participants completed the evaluation. - Intervention Group: 86% (n=235/273) - Control Group: 93% (n=101/109)   **Dropout reasons:** N/A | **QoL:**   - Mean HADS scores not statistically significant between groups - Medium/severe HADS scores (>8) was common in participants preferred printed DA - DA users who preferred online format had lower HADS score (<8)   **Positive effects:** Patients using DA felt more knowledgeable but did not score better on knowledge.  **Negative effects:**   - No significant difference in decisional conflict between DA & control groups - DA users reported lower satisfaction with information provision. - Patients with anxiety or depression had higher decisional conflict in the DA group compared to the control group. | **Setting:**   - hospitals (n=18) - DA was incorporated into standard care   **Provider/staff:**   - Urologists & oncology nurses introduced DA and provided access cards with individualised treatment. - No formal or intensive training required | **Cost:** N/A  **Enablers:**  structured treatment information.  Useful online formats  **Barriers:**  Some patients preferred a printed DA.  Patients with anxiety & depression had worse decision-making experiences.  Some patients expected the DA to provide a treatment recommendation, which it did not. | N/A | - Limited power for subgroup analysis due to few control participants | - The web-based DA did not reduce decisional conflict or improve involvement compared to **standard** counselling. - Patients using the DA felt more knowledgeable but were less satisfied with the information provided. - additional decision support may be needed for patients experiencing anxiety and depression |
| Cuypers 2019  **Country:**  The Netherlands  **Design:** RCT  **Aim:** Evaluate implementation, uptake, & usage of web-based PCa treatment DA in routine clinical practice | **Population:**  Patients newly diagnosed with LPCa who had at least two treatment options  **Sample Size:** N=351  **Age:**   - DA *M=*65.2 yrs - Non-users *M=*65.6 yrs   **Stage:** N/A  **Treatment:**   - Surgery - RT   **Other:**   - Married / partnered (90%) - Gleason Score 6 (DA users 64%; non-users 40%) - Gleason Score 7 (DA users 36%, non-users 60%) | **Intervention name**: N/A  **Supportive care type:**  Web-based PCa DA, including values clarification exercises (VCEs)  **Mode of Delivery:**  Online  **Duration:** Within a week of diagnosis & before the treatment decision. | **Preference measures**:   - Uptake/ usage - Usage - Experience with DAs   **Preference tools**:   - Questionnaire - Values clarification Exercises (VCEs)   **QoL measures:** N/A | *Uptake*:   - 35% of eligible patients received DA (hospital range: 16%–84%) - 79% of those who received it accessed the tool. - Highest uptake occurred in hospitals involved in DA development   *Experience*   - DA users discussed DA summary with a clinician (71%) - Had treatment preference (71%) - Felt sufficient explanation was sufficient (85%) | **Sample Size:** n=351  **Refusal Rate:** N/A  **Refusal reasons:** N/A  **Participation Rate:** 79% (277/351) accessed the DA.  **Dropout reasons:** N/A | **QoL:** N/A  **Positive effects:**   - Most DA users engaged in shared decision-making with their provider. - DA users felt more knowledgeable & reported better alignment between treatment decisions & values.   **Negative effects:**   - DA uptake lower than expected (35%). - Patients with higher Gleason scores were less likely to engage with DA.   **Cost-Effectiveness:** N/A | **Setting:**   - Urologist departments in Dutch hospitals (n=18) - DA distribution added to existing clinical workflows.   **Provider/staff:**   - Urologists & oncology nurses introduced DA & provided access cards with individualised treatment. - Nurses actively participated in DA delivery, uptake rates were higher. | **Cost**: N/A  **Enablers:**  well-integrated into hospital routines  Distribution by nurses improved uptake.  sufficient explanation  **Barriers:**   - Varied distribution across hospitals (16%–84% uptake rate). - Limited nurse involvement hindered adoption. - Some p preferred paper DA over online version. - Patients with anxiety or depression require additional support. | **Organisational/Staff Level:**  Successfully implemented in urologist departments at 9 hospitals  **Individual Level:**  N/A | - Variation in implementation across hospitals (likely due to differences in staff engagement). - No long-term outcomes on patient satisfaction or treatment adherence. - Did not assess cost-effectiveness. | - most patients who received DA engaged with it & used it to support shared decision-making but overall implementation was lower than expected (35% uptake). - Involvement of nurses improved DA distribution, but additional strategies are needed to ensure broader & more consistent DA adoption across clinical settings. |
| Cuypers 2019  **Country:**  The Netherlands  **Design:**  Cluster RCT  **Aim:**  Investigate effect of online DA during PCa treatment counselling on decisional regret & information satisfaction | **Population:**  Patients newly diagnosed with localised low- or intermediate-risk PCa who had at least two treatment options  **Sample size:** N=382   - DA group (n=235) - Control (n=100)   **Age:**  DA *M=* 64.9 yrs  Control *M=*66.2 yrs  **Stage:**  LPCa  **Treatment:** N/A (new diagnosis)  **Others:**   - Married/partnered: (86%) - Highly educated (36%) - Gleason Score 6 (DA users 61%) | **Intervention name**: N/A  **Supportive care type:**  Web-based PCa DA, including values clarification exercises (VCEs)  **Mode of Delivery:**  Online  **Duration:** before treatment decision | **Preference measures:**   - Regret after treatment - Satisfaction   **Preference tools**:   - Decision Regret Scale (DRS) - Satisfaction with Cancer Information Profile (SCIP-B) - VCEs   **QoL measures:**   - HADS | - 15% of patients regretted their treatment choice, regardless of DA use at 12 months - 36% of patients were dissatisfied with the information received at 12 months | **Sample Size:** N=384 enrolled.  **Refusal Rate:** N/A.  **Participation Rate:**   - Overall: 89% (340/384) of participants signing consent received control/intervention. - Intervention: 84% (229/273) of consenting participants received intervention. - Control: 100% (111/111) of consenting participants received control.   **Dropout reasons:** N/A | **QoL Measures:** N/A  **Positive effect:** N/A  **Negative effect:** Patients with anxious or depressive symptoms were more likely to regret their decisions & feel dissatisfied with the information provided  **Cost-Effectiveness:** N/A | **Setting:**   - hospitals (n=18) - Adoption required only inclusion of DA distribution in standard consultation procedures   **Provider/staff:**   - Urologists & nurses introduced the DA & provided access cards with individualised treatment - Staff adaptation minimal, primarily involving DA explanation and encouragement of use during routine post-diagnosis consultations. | **Cost: N/A**  **Enabler:**  Accessible  **Barriers:**   - Anxiety & depressive symptoms influenced dissatisfaction & regret.   The DA did not appear to impact long-term decision outcomes. | N/A | - fewer patients in the control group. - Power limited for subgroup analyses. - Anxiety & depression may have influenced results but were not targeted by the DA. | - Exposure to an online DA did not reduce regret or improve information satisfaction 12 months after treatment. - Anxious & depressive symptoms were associated with increased regret & dissatisfaction, suggesting that psychological factors may play a larger role in decision-making than access to DAs. |
| Cuypers 2020  **Country:** The Netherlands  **Design:** Quasi-experimental  **Aim**:  Assess implementation rates of 3 PCa treatment DAs in routine care & evaluate patient-perceived barriers & facilitators to DA usage. | **Population** Patients newly diagnosed with LPCa.  **Sample size** N=908   - DA1 (n=225) - DA 2 (n=183) - DA 3 (n=235)   **Age**   - DA1 *M=*66.0 yrs - DA2 *M=*66.3 yrs - DA3 *M=*64.9 yrs   **Stage:** LPCa  **Treatment: N/A** | **Intervention name**: N/A  **Supportive care type:**  DAs designed to support shared decision-making in PCa treatment  **Mode of delivery:**   - DA1: Concise booklet (14 pages), available during consultation or at home. - DA2: Hybrid (booklet or online) chosen by patient. - DA3: Elaborate online DA with VCEs   **Duration:** before treatment decision | **Preferences measures:**   - Usability & acceptability - Satisfaction   **Preference tools:**   - Questionnaire (survey) - 24-item list of barriers & facilitators - Implementation rate calculations - VCEs   **QoL measures:** N/A | - Received sufficient explanation about the DA (85%) - Satisfaction with the format: DA1 (99%), DA2 (96%), DA3 (21%) - Participants preferred paper-based DAs | **Sample Size:** n=908  **Refusal Rate:** N/A  **Reasons for Refusal:** N/A  **Participation:** 40% (908/2285) of eligible participants were enrolled in the study & received the intervention (implementation rate).  **Dropout reasons:** N/A | **QoL Measures:** N/A  **Positive effects:**   - Improved patient satisfaction & engagement in decision-making. - DAs were considered a valuable addition to **standard** care   **Negative effects:**  Some patients found DAs unpractical or not personalized  **Cost-effectiveness:**  N/A | **Setting:**   - hospitals (n=33) across 3 regions. - Highest uptake in hospitals familiar with/involved in DA development   **Provider/staff:**   - Urologists and nurses introduced & provided access to DAs - Nurse involvement often boosted success - Staff not required to discuss DAs in follow-up but patients could raise them in conversation | **Implementation:**  implementation rate: 40%  Some hospitals had high (>80%) implementation; others ranged from 2–80%.  Enabler:  DA1 & DA2 could be incorporated in clinical consultation & at-home  **Barriers:**   - DA designed for use outside consultation - Unpractical, unadjusted to needs, no user confidence, uncertainty. | N/A | - Variability in hospital implementation rates. - Implementation rate based on estimated eligibility rather than actual numbers. - Patients who received a DA but didn’t complete surveys were N/A. - Non-randomized DA distribution limits generalizability. | - Patients were generally satisfied with DAs, especially concise paper versions. However, sustained implementation in routine care requires additional encouragement & monitoring. |
| daMata 2019  **Country:** Brazil  **Design:**  Parallel single-blind RCT  **Aim:**  Evaluate effectiveness of home care teaching program for patients undergoing RP based on self-efficacy from Cognitive Social Theory. | **Population**: Men undergoing RP for PCa  **Sample Size:** N=68   - Intervention (n=34) - Control (n=34)   **Age**: *M=*64.2 yrs  **Stage**: N/A  **Treatment**: RP  **Other**   - Education yrs (Intervention *M=*6.9 (SD = 4.8); Control *M=*5.5 (SD = 4.8)) - Married (97.1%) - Comorbidities (73.5%) | **Intervention name**: N/A  **Supportive Care Type:**  Structured teaching program for post-discharge home care, including combination of oral guidance, booklet “Guidelines for Home Care: Radical Prostate Surgery”, & phone follow-ups.  **Mode of delivery**:   - Booklet - Phone   **Duration:** Two phone follow-ups post-hospital discharge:   - phone 1: 3-5 days (15 min) - phone 2: 30 days post-discharge (12 min) | **Preference measures:**   - Satisfaction/ Effectiveness   **Preference tools:**   - Self-efficacy Scale - Single-item Satisfaction Score (1-5 scale)   **QoL measures**:   - HADS - Home Care Knowledge Questionnaire (23 items) | Satisfaction significantly higher in intervention than control group | **Sample Size:** N=75    **Refusal Rate**: 7% (7/96) refused participation.  **Reasons for Refusal: N/A**    **Participation:**   - Overall: 91% (n=68/75) received allocated condition. - Intervention: 97% (n=34/35) received allocated condition. - Control: 85% (n=34/40) received allocated condition.   **Dropout reasons**:   - Stroke - Not returning - , - prolonged use of UCD - contamination in the - intervention. | **QoL measures:**   - Significantly higher knowledge in intervention group - Significant reduction in intervention group anxiety   **Positive Effects:**   - Increased patient knowledge of post-discharge care - Improved patient satisfaction - Reduced anxiety levels in intervention group   **Negative effects: N/A**  **Cost-effectiveness:**  N/A | **Setting:**   - Surgical units in hospitals (n=3) - Implemented during routine inpatient care & post-discharge period - Easily integrated into existing discharge processes with no reliance on digital systems or hospital IT.   **Provider/staff:**   - Two trained nurse researchers conducted intervention - Intervention feasible within standard nurse-patient ratios; required minimal training and no additional clinical staff. | **Cost:**  N/A  **Enablers**  Structured booklet, phone-based reinforcement, & use of self-efficacy principles  **Barriers:**  N/A. | Organizational Level:  No information on long-term implementation in hospital settings  Individual Level:  No follow-up beyond the 2-month study period | - Limited to a single geographic region in Brazil - Study only followed patients for 2 months post-discharge | - The program, was effective in increasing patient satisfaction & knowledge about home care. - No significant improvement was observed despite the intervention - Improved communication between patients & HCP was crucial for these outcomes. |
| Davis 2013  **Country:** USA  **Design:** RCT  **Aim:** Compare impact of technology-assisted symptom monitoring system vs usual care on HRQOL & doctor/patient communication in PCa survivors. | **Population:** PCa survivors 10–19 months post-treatment  **Sample Size:** N=94   - Intervention (n=49) - Control (n=45)   **Age:** *M=*62 yrs  (range 48–78 yrs)  **Stage:** Early-stage PCa  **Treatment:**   - RP - RT - ADT - Watchful waiting   **Other:**   - African American (61.7%) - ≥College (50%) - Married/partnered (78.7%) - Employed full-time (60.6%) - **I**ncome >$100,000 (57.4%) - ≥Two comorbidities (43.6%) | **Intervention name**: N/A  **Supportive Care Type:**  technology-assisted symptom monitoring system where participants used phone-based system to report symptoms before follow-up visits  **Mode of delivery:**   - Automated phone system (PCa Monitoring System, PCMS**)**   **Duration:** 3 days before follow up visits over ~7 months | **Preferences measures:**  Satisfaction/  **Preference tools:**   - Evaluation questionnaire: questionnaire for control group   **QoL measures:**   - SF-12: Mental component Summary (MSC) & Physical Component Summary (PSC) - Doctor/Patient Communication: Primary Care Assessment Survey (PCAS) - General HRQOL: SF-12 - Cancer-Specific HRQOL: Functional Assessment of Cancer Therapy – General (FACT-G) - PCa-Specific HRQOL: UCLA PCa Index (UCLA-PCI) (urinary, bowel, & sexual function) - Post-Visit Ratings (PVR): Evaluations from patients & physicians | - monitoring system easy/very easy to use - 85% believed all patients would benefit from it - 62% preferred to use system before each visit, 14% every other month, 5% monthly, 19% never - 96% of intervention completed at least one monitoring session, 83% completed both | **Sample Size:** N=94    **Refusal Rate:**  27% (38/142) of men who were contacted declined participation or could not be reached.  **Reasons for Refusal:** N/A    **Participation:**   - Overall: 93% (87/94) completed T1 condition, & 74% (70/94) completed T2 condition. - UC: 91% (41/45) completed T1 condition, & 71% (32/45) completed T2 condition. - Intervention: 94% (46/49) completed T1/condition, & 78% (38/49) completed T2 condition.   **Drop out reasons:**   - Discomfort discussing PCa - too busy - confidentiality concerns - illness - moved away. | **QoL measures:**   - No significant differences in general or cancer-specific HRQoL - improved sexual functioning in intervention group among African American men compared to White men - Improved doctor/patient communication and mental health in African American men in intervention compared to African American men in control   **Negative Effects:** N/A  **Cost-Effectiveness:** N/A | **Setting:**   - hospitals (n=2) - Limited adoption due to minimal physician training and engagement - **Provider/staff:** Urologists & radiation oncologists received symptom reports before patient visits - Low physician involvement. Additional training needed for future implementation | **Cost: N/A**  **Enablers:**   - Automated system - reminders call for non-adherence - structured monitoring process   **Barriers:**   - Variability in follow-up care across treatment types - Low physician engagement with reports - Potential for contamination between groups since physicians treated both intervention & control patients | N/A | - High baseline HRQOL Limited ability to detect changes - Follow-Up varied due to care differences between RP & RT | - Although there was no overall effect of the technology-assisted symptom monitoring system, exploratory analyses suggest that it may be particularly beneficial for African American men, improving their sexual function, doctor/patient communication, & mental HRQOL. - Future studies should include larger samples, more intervention points, & increased physician training to maximize impact. |
| Davison 2014  **Country**: Canada  **Design**: One-arm quasi-experimental  **Aim**: Measure preferences & values of men newly diagnosed with PCa using a web-based decision support technology | **Population:** Men newly diagnosed with LPCa  **Sample Size:** N=49  **Age**: *M*=60.5 yrs (SD=6.7)  **Stage**: LPCa  **Treatment:** N/A (newly diagnosed)  **Other:**   - Caucasian (96%) - Married (87.8%) - Highschool or less (51%) - Rural location (57.1%) | **Intervention name**: N/A  **Supportive Care Type:** Decision support intervention for PCa (DSI-PC) utilised prior to consultation  **Mode of delivery:**  Web-based  **Duration:** N/A | **Preference measures**:   - Satisfaction - Values - Health information-seeking behaviour - Factors influencing treatment decision   **Preference tools:**   - satisfaction & utility questionnaires   **QoL measures:**  N/A | - High levels of satisfaction with treatment decision & involvement of support in decision making - 61% shared summary sheet with their physician - 63% wanted access to in-depth or detailed information | **Sample Size:** N=49    **Refusal Rate:** 8% (n=4/53) of individuals approached declined to participate.    **Reasons for Refusal:** N/A    **Participation:** 94% (n=46/49) completed measures prior to the treatment consultation & again after a treatment decision had been made.  **Dropout reasons**: N/A | **QoL measures**: N/A  **Positive Effects:**   - Significantly lower levels of decision conflict after a treatment decision was made - High satisfaction with information received & involvement in decision making   **Negative Effects:** N/A  **Cost-Effectiveness**: N/A | **Setting:**   - Urology outpatient clinic - DSI-PC used at home or at education centre   **Provider/Staff:**  Nurse educator provided education sessions; physicians (urologists and radiation oncologists) incorporated summary sheet information into discussions   - Physicians instructed on how to use summary sheet in discussions | **Cost:** N/A  **Enablers:**  Web-based access, summary sheet for treatment discussions  **Barriers:** N/A | N/A | - Small sample size - Lack of randomized control study design - Need for further testing in larger centres with diverse populations | The DSI-PC intervention may be a useful tool to help patients identify & communicate their values & preferences to physicians at the time of treatment discussions, leading to high levels of patient satisfaction & reduced decision conflict. |
| desBordes 2017  **Country:** USA  **Design:** Pre-post pilot  **Aim:** Develop & evaluate online educational tool to promote bone health in PCa & BCa survivors. | **Population:** PCa & Bca survivors at risk of osteoporosis  **Sample Size:** N= 20   - PCa (n=10) - BCa (n=10)   **Age:** *M*=63.2 yrs  **Stage: N/A**  **Treatment:** ADT  **Other:**   - White (70%) - Married (80%) - ≥Highschool (80%) - Employed (60%) - Adequate Health Literacy (90%) | **Intervention name**: N/A  **Supportive Care Type:**  Online educational website with videos, images and animations to promote bone health in PCa & BCa survivors  **Mode of delivery:**  Online  **Duration:** Participants navigated website at their own pace in one clinic session | **Preference measure(s):**   - Acceptability - Satisfaction:   **Preference tools:**   - Ottawa Acceptability Scale (OAS) - Usability, Satisfaction, & Ease of Use (USE) questionnaire   **QoL measures**: Effective Consumer Scale (ECS). | - High OAS acceptability (60% -100%) - All (100%) of participants found website helpful, easy to use, and well-organised | **Sample Size:** N=20    **Refusal Rate:** 79% (76/96) of eligible participants did not enrol.    **Reasons for Refusal:** Conflicting schedules.    **Participation:**   - Intervention completion N/A - 90% (18/20) completed follow-up questionnaire.   **Dropout reasons:** N/A | **QoL:** PCa survivors showed an increase in self efficacy (confidence for physical activity & calcium intake), but results not statistically significant  **Positive Effects:** Knowledge increased 1 month after intervention  **Negative Effects:** N/A  **Cost-effectveness:** N/A | **Setting:**   - Cancer centre - Website made publicly available through MD Anderson’s website   **Provider/staff:**   - educational content designed, reviewed, & managed by multidisciplinary research team, including physicians, health communication researchers, patient educators and eHealth technology developers. - No routine clinical staff involved in delivering or promoting intervention | **Success:** Participants easily navigated the website & completed all required tasks  **Cost: N/A**  **Enablers:**   - Use of videos, animations, & interactive tools to improve engagement. - Clear layout & readability of the website (Grade 7-8).   **Barriers:**   - Low participation rate (21%) due to recruitment difficulties. - Limited sample diversity (mostly White, well-educated individuals). - No physician involvement in delivering the intervention. | **Organizational Level:**  The website remains accessible on MD Anderson’s institutional platform.  **Individual Level:**  No long-term tracking of continued website use. | - Low Participation Rate (21%) limits generalizability. - Short Follow-Up Period (1-month), preventing long-term outcome evaluation. - Small sample size - Limited Diversity: Majority White, well-educated participants. - No Physician Engagement in the intervention. | - The online educational website improved knowledge about bone health in PCa & BCa survivors & was well-received by participants. However, self-efficacy & disease management did not significantly improve. - Future studies should increase sample size, diversify participants, & incorporate physician engagement to enhance impact. |
| Diefenbach 2004  **Country:** USA  **Design:** Mixed method  **Aim:**  Develop & evaluate multimedia interactive education system in treatment decision-making. | **Population:**  Men diagnosed with LPCa & partners  Sample Size: N=33   - Men with PCa n=18 - Partners n=15)   *Note: PCa patients reported below*  **Age:** *M*=66 yrs (SD = 8)  **Stage:** Early stage (tumours confined to prostate)  **Treatment:**   - Surgery - RT - brachytherapy   **Other:**   - White (61%) - High school graduates (33%) - Married (83%) - Retired (60%) - Computer access (65%) - Internet access (70%) - familiar with computer (80%) - family support with computer issues (76%) | **Intervention name**:  Prostate Interactive Education System (PIES)  **Supportive Care Type:** multimedia interactive education & decision-making tool that offers tailoring and virtual health centre metaphor (i.e., participants navigate “rooms” to access information)  **Mode of delivery:** Internet and CD-ROM  **Duration:**   - Average 3 hours per week - Total duration N/A | **Preference measures:**   - Interest, usefulness, & willingness to use PIES   **Preference tools:**   - Preference Ratings - Focus group evaluations   **QoL measures: N/A** | - Interest & usefulness scored high (*M*=4.71/5) - Preferred software over books/pamphlets (83%) but wanted it in addition to print materials   (100%)   - Willing to spend time 1-2 hours & pay $50 on average if program was commercially available - Highly rated virtual health centre metaphor as intuitive & engaging - Liked accessing information in any order. - Requested virtual guide for navigation - Library valued for combining text with videos & illustrations - Men liked hearing real experiences from survivors in support group videos | **Sample Size:** N=33 (18 men with PCa, 15 spouses)    **Refusal Rate: N/A**    **Reasons for Refusal: N/A**    **Participation:** 100% (18/18) of men with PCa participated in focus group after using the intervention/tool. | **QoL measures:** N/A  **Positive Effects:**   - High acceptability among patients & spouses - Participants felt it provided useful decision-making support   **Negative Effects:** N/A  **Cost-Effectiveness:** N/A | **Setting:**   - Cancer Centre and a university - Patients used PIES independently at home/in a quiet setting with no direct infrastructure requirement   **Provider/Staff:**   - No direct clinical staff involved in deploying or facilitating tool in focus groups, or implementation - One psychologist linked to PIES reviewed reports generated from patient interactions with software. | **Cost: N/A**  **Enablers:**   - User-friendly interface (virtual rooms, interactive videos, multimedia content) - Tailoring of information to patient preferences   **Barriers:**   - Need for improved navigation assistance (requested a virtual guide/tutor) - Some participants had limited digital literacy and a minority had no personal computer (18%) or internet access (6%) - 40% did not use internet to access PCa information | N/A | - Preliminary study – no long-term outcomes measured - Small sample - Participants were already past treatment decision-making stage - PIES not evaluated in real-world conditions | - The PIES multimedia education system was highly accepted by PCa patients - Further testing in a randomized trial is needed to evaluate long-term effectiveness. |
| Diefenbach 2012  **Country**: USA  **Design**: Three-arm unblinded RCT  **Aim**: Examine acceptability & feasibility of educational intervention | **Population:** Men diagnosed with PCa considering treatment options  **Sample Size:** N=91   - Intervention + tailoring (n=32) - Intervention (n=21) - Control (n=19)     **Age:** *M=*61.9 yrs (SD = 8.1) yrs  **Stage**: Early-stage (T1–2N0M0)  **Treatment:** N/A (newly diagnosed)  **Other:**   - White (59%) - College-educated (56%) - Married/partnered (72%) - Employed (49%;) - Access to home internet (87%) | **Intervention name**: Prostate Interactive Educational System (PIES)  **Supportive Care Type:**  Multimedia DA for PCa treatment decision-making presented as virtual health centre  **Mode of delivery:**  Internet and CD-ROM  **Duration:** 45-minute/session | **Preference measure(s):**   - Usability - Confidence, information needs, & decisional clarity - Information-seeking style - Acceptability - Feasibility   **Preference tools:**   - 17-item questionnaire on usefulness, satisfaction, clarity, emotional reassurance - Decisional Conflict Scale - Monitoring/Blunting Style Scale - (Impact of Event Scale-Revised)   **QoL measures:** N/A | - Satisfied with the educational materials - No differences between PIES tailored & non-tailored | **Sample Size:** N=91    **Refusal Rate:**  25% (30/121) of referred patients did not enrol in the study.   - **Reasons for Refusal:** Lack of time & interest - made a treatment decision.     **Participation:** 79% (n=72/91) of enrolled participants completed the study.  **Dropout reasons**: lack of time. | **QoL Measures:**  N/A  **Positive Effects:**   - Increased decision confidence. - Reduced confusion & anxiety related to decision-making. - Better understanding of diagnosis, treatment, & side effects   **Negative Effects:** None  **Cost-effectiveness:** N/A. | **Setting:**   - Urban medical centre (n=1) - Intervention integrated successfully into pre-consultation workflow.   **Provider/Staff:**   - Research staff facilitated recruitment & informed consent - No clinical staff involved. Research assistants provided support; no major barriers reported. | **Cost:** N/A  **Enablers:**  High patient access to computers & internet, supportive staff, engaging interface.  **Barriers:**   - patients sometimes called to appointments before completing assessments - Limited tailoring (only applied to text in library section). | N/A | - Small sample size & pilot nature; limited statistical power - Sample not fully representative; overrepresentation of educated; White patients. - Short follow-up duration (only immediate post-intervention measured) - Tailoring was minimal & limited to library content. | PIES acceptable & feasible for patients with early-stage PCa. It significantly improved patients’ confidence & emotional clarity in treatment decision-making, though tailoring to information-seeking style did not provide added benefit. |
| Diefenbach 2018  **Country:**  USA  **Design:**  RCT  **Aim:**  Evaluate impact of multimedia educational & DA program on decisional conflict & distress among men newly diagnosed with early-stage PCa | **Population:**  Men diagnosed with LPCa who had not yet made a treatment decision  **Sample Size:** N=349   - Intervention (n=181) - Control (n=168)   **Age:** *M=*64.7 yrs  **Stage:** LPCa  **Treatment:** N/A  Other   - White (76.2%) - ≥College (54.2%) - Income   ≥$80,000 (30.7%)   - Comorbidities (82.8%) - Medically insured (91.6%) | **Intervention name**:  “Healing Choices for Men with Prostate Cancer”  **Supportive Care Type:**  An interactive, multimedia DA designed to provide education, emotional support, & decision-making tools versus control (standard consultation only)  **Mode of delivery:**   - Online - CD-ROM   **Duration:** 2 months | **Preference measures:**   - Acceptability   **Preference tools:**   - DCS - Impact of Event Scale (IES): - Charlson Comorbidity Index   **QoL measures:** N/A | P Increased decision support among intervention participants overall, African American participants, and those with lower education  **Subgroup Preferences:** | **Sample Size:** N=349    **Refusal Rate:** Not explicitly stated.  **Reasons for Refusal:** Not explicitly stated.    **Participation:**   - Overall, 80% (349/440) of enrolled participants completed 2-mo assessment.   **Dropout reasons:**   - No computer access (30%) - No need for additional information (17%) - Technical difficulties with the computer (15%) - Lack of time (13%) - Unfamiliarity with multimedia programs (9%). | **QoL Measures:** N N/A  **Positive Effects:** No differences in cancer-related distress or overall decisional conflict  **Negative Effects:** N/A  **Cost-Effectiveness:** N/A. | **Setting:**   - National Cancer Institute’s Cancer Information Service (CIS) used for recruitment - CIS provided intervention to patients as an adjunct to phone counselling. - No report integration into routine clinical care.   **Provider/Staff:**   - Cancer information specialists provided standardised phone consultations - Minimal engagement from clinicians beyond CIS specialists. | **Cost: N/A**.  **Enablers:**   - Existing CIS infrastructure helped with recruitment. - Multimedia format increased engagement for some subgroups.   **Barriers:**   - Technical issues (15% dropped out due to computer difficulties). - Limited digital literacy among some participants. - Attrition higher in younger, more distressed patients with comorbidities. | **N/A** | - Short follow-up period (2 months). - High attrition among younger & more distressed patients. | - While the multimedia DA improved decision support, it did not significantly reduce decisional conflict or distress. - African American men & those with lower education benefitted the most, suggesting that DAs should be tailored to specific patient subgroups. |
| Diefenbach 2023  **Country:** USA  **Design:**  RCT  **Aim:** Evaluate efficacy of brief interactive web-based DA | **Population:** Men diagnosed with LPCa who had not yet made a treatment decision  **Sample Size:** N=327   - Intervention (52.3%) - Control (47%)   **Age:** *M=*64.2 yrs (SD = 6.9)  **Stage:** LPCa (T1, T2, N0, M0)  **Treatment:** N/A  **Other:**   - White (76.1%;) - Non-Hispanic (96.5%) - ≥University (3.5%) - Employed (50.9%) - Married/living with partner (78.4%**)** - Income >$75,000 (65%) - Comorbidities (82.8%;) - Health Literacy (Newest Vital Sign Score) Adequate (60%) | **Intervention name**:   - Healium, vs - Healing Choices (control)   **Supportive Care Type:**  interactive web-based DA designed to help patients elicit treatment preferences before discussing options with HCP  **Mode of delivery:**   - Healium: tablet in clinic - Healing Choices: multimedia videos   **Duration:**   - Healium: 10-13 min/ session - Healing Choices: Several hours of content (self-paced, online) | **Preference measures:**   - Usability of tool   **Preference tools:** N/A  **QoL measure:**   - Anxiety/Tension (Profile of Mood States - POMS subscale) - Depression (POMS subscale) | - Healium rated as more user-friendly for low-health literate populations. - High completion rates (majority of patients completed the tool before consultation). | **Sample Size:** N=327    **Refusal Rate:** Not explicitly stated.    **Reasons for Refusal:** Not explicitly stated.    **Participation:** Overall, 100% (327/327) of participants received allocated condition, including 100% receiving the intervention (171/171). | **QoL:**   - Anxiety/Tension: Small decrease, not significant in either group. - Depression: Significant decrease in both groups at 6 weeks (Healium p = .001, Healing Choices p ≤ .001). - patients had significantly higher depression scores than those choosing RT or surveillance at 6 weeks (p = .04). Differences no longer significant at 3 months.   **Positive effects:**   - Decisional conflict significantly decrease from T1 to 6 weeks in both groups (p ≤ .001); non-significant decrease at 3 months - Significant increase in decision satisfaction in both groups from 6 weeks to 3 months   **Negative effects:**  N/A  **Cost-effectiveness:**  N/A | **Setting:**   - Major cancer centre (n=2) - program was embedded within treatment decision-making - Potential for integration into Electronic Medical Records (EMR) and routine clinical workflow.   **Provider/Staff:**   - Study coordinators introduced tool in clinic - Physicians encouraged but not required to review patient preferences before consultation - No clinical staff directly involved in intervention delivery | **Cost:** N/A.  **Enablers:**   - Designed for low-health literate populations. - Brief intervention time allowed easy integration into clinical practice   **Barriers:**   - Limited racial diversity in sample (predominantly White participants). - Not yet fully integrated into EMR systems. | **N/A** | - Limited Diversity: Predominantly White & well-educated sample. - Short Follow-Up Period: Limited to 3 months. - No Direct EMR Integration Yet: Future work needed to embed Healium into patient records for physician access. | The Healium DA was as effective as Healing Choices in reducing decisional conflict but was significantly shorter & more accessible for low-health literate populations. |
| Evans 2021  **Country**: Australia  **Design**:  Mixed method  **Aims:**  Examine & refine usability & acceptability of computer-tailored web-based exercise intervention; & assess safety & participants’ ability to replicate video-guided resistance exercises. | **Population:** Men with metastatic PCa  **Sample Size**: N=11   - Cycle 1 (n=5) - Cycle 2 (n=6)   **Age**:  *M* = 73.4 yrs (SD = 6.7;  range: 63–82 yrs)  **Stage**: Metastatic  **Treatment:**   - ADT (82%) - Chemotherapy (18%;)   **Other**   - Married (91%) - University (36%) - Retired (64%) | **Intervention name**:  ExerciseGuide  **Supportive Care Type:** Personalised, computer-tailored web-based multimodal exercise intervention including video demonstrations, modules, & tailoring  **Mode of delivery:**  Website  **Duration:** 1 x 90 –120-minute session | **Preference measures:**   - Acceptability - Usability - User feedback   **Preference tools:**   - System Usability Scale (SUS) - 6-item acceptability scale (5-point Likert) - Think-aloud protocol - Qualitative interviews   **QoL measures:**   - SF-12 (Physical component-PSC12 & mental component-MCS-12) | - Usability improved between cycles (cycle 1 *M* SUS = 60; cycle 2 *M* = 69.6) - High acceptability across both cycles (*M* = 25.4/30) - All would recommend to others (100%) - Appreciated tailoring & flexibility & expert support options - Preferred video over text exercise; functionality over appearance - Challenges included technical language, dense content, and lack of printable versions | **Sample Size**: N=11    **Refusal Rate**: N/A  **Reasons for Refusal**:  N/A    **Participation Rate**:  100% | **QoL:**  Quality of Life Measures: SF-12 (PCS, MCS) reported at baseline  **Positive Effects:**   - Improved confidence in completing exercises safely - Felt information was credible & useful   **Negative Effects:**   - Minor pain in 2 participants (not bone-related)   **Cost-Effectiveness:** Not evaluated | **Setting:**   - University laboratories (n=2) - Intervention designed for home use.   **Provider/staff:**   - Research staff administered intervention - Independent exercise physiologists with ≥5 years of experience assessed safety and technique of exercises via video review. - Iterative feedback loop used for tailoring & revision; user-informed changes adopted | **Cost:** N/A  **Enablers:**   - Tailored, relevant content - Video demonstrations - Modular format   **Barriers:**   - High cognitive load for some users - Technical literacy challenges - Low interrater reliability in movement scoring   **Other:**   - Suggested improvements (e.g., more aerobic options, visual cues, simpler definitions) - Safety confirmed by expert review & participant self-reports | **N/A** | - Small, non-diverse sample (mostly Caucasian, tech-literate men) - No aerobic safety testing - Low interrater reliability in exercise scoring - Website was an abridged prototype | ExerciseGuide was found to be usable & acceptable by men with metastatic PCa, who preferred personalized video-based support & highlighted the importance of simple, functional design over aesthetic appeal. |
| Engler 2016  **Country**: Germany  **Design**:  Mixed method  **Aim:**  Investigate how cancer patients use & perceive narrative-based website that shares experiential stories from others with cancer | **Population:** Individuals diagnosed with BCa, PCa or CCa  *Note: PCa subgroup reported on*  **Sample Size:** N=56   - PCa (n=23)   **Age:**   - 50–59 (12%) - 60–69 (53%), - ≥70 (35%, 6),   **Stage: N/A**  **Treatment:** N/A | **Intervention name**:  krankheitserfahrungen.de  **Supportive Care Type:**  Peer-based narrative health information via multimedia (text, video, audio)  **Mode of delivery:**  Website  **Duration:** Two weeks of unguided access to website site prior to evaluation | **Preference measures:**   - Attitudes - Value - Usage   **Preference tools:**   - website navigation - Focus group/interviews   **QoL measures**: N/A | - High engagement across modules; person pages & videos heavily accessed - Website information was valuable (84%) and content diverse (92%) | **Sample Size:** N=56.    **Refusal Rate:** N/A  **Reasons for Refusal: N/A**.    **Participation:** 100% of participants used the intervention/tool. | **QoL Measures:**  N/A  **Positive Effects:**   - Emotional support - Reassurance - Felt less alone - Reflected on their own decisions & identity   **Negative Effects:**   - Some participants found videos emotionally overwhelming over time   **Cost-Effectiveness:** N/A | **Setting:**   - intervention deployed as a web-based, public-access platform hosted by academic institutions - No setting adaptations needed.   **Provider/staff:**   - Researchers and moderators for interviews; not a clinical setting - Developed & maintained by academic researchers; no clinical staff involved | **Cost:** N/A  **Enablers:**   - Filter options for personalization - Academic credibility of content - Inclusion of video/photos increased authenticity   **Barriers:**   - Lack of medical detail (e.g., PSA or Gleason scores for PCa) - Limited filter options for certain clinical features   **Other:**   - Users preferred content that could be quickly navigated; emotional readiness influenced media preferences | **N/A** | - Small sample sizes, especially for colorectal cancer group - Technical issues led to incomplete log data - Short duration of website access (2 weeks) - Lack of quantitative health outcome measures | Cancer patients valued relatable peer narratives presented with academic rigor. Preferences leaned toward authenticity; diversity of experience, & navigable formats tailored to individual characteristics. |
| Evans 2021  **Country:** Australia  **Design**: Two-arm pilot RCT  **Aim:** Examine acceptability, safety, feasibility, & preliminary efficacy of a web- & phone-based personalized exercise intervention for individuals with metastatic PCa. | **Population**:  Men diagnosed with metastatic PCa  **Sample Size:** N=40   - Intervention (n=20) - Control (n=20)   **Age:** *M=*70.2 yrs (SD = 8.5)  **Stage:** Metastatic  **Treatment**   - ADT (92.7%) - Chemotherapy (56.1%) - RT (46.3%) - Previous surgery (31.7%)   **Other:**   - Married/partnered (68.3%) - ≥University (36.6%) - Retired (70.8%) - Major cities (63.4%) | **Intervention name**:  ExerciseGuide  **Supportive Care Type:** Tailored exercise & behaviour change support for aerobic, resistance, & flexibility training  **Mode of delivery:**   - Web-based program - telehealth (x2 consults) - SMS/email   **Duration:** 8 weeks (usage time *M*=93.3 ± 101.6 mins) | **Preference measures:**   - Acceptability - Satisfaction   **Preference tools:**   - Client Satisfaction Questionnaire-8 (CSQ-8) - SUS - Perceived Environmental Supportiveness Scale (PESS) - Personal relevance items (3 Likert-scale questions) - Website usage metrics - Open-ended feedback   **QoL Measures:**   - EORTC QLQ-C30) - Functional   Assessment of Chronic Illness Therapy-fatigue subscale (13-items)   - HADS - Pittsburgh   Sleep Quality Index questionnaire | *Satisfaction:*   - High satisfaction, usability, perceived supportiveness, and module satisfaction - Positives: Personalized support, structure, motivational elements - Challenges: Lack of exercise variety, boredom, navigation issues - Suggestions: More variety, more expert contact   *Acceptability*   - Completed exercise diaries (89%) - 100% Completed key modules (100%) - Weekly site logins highest in weeks 1 & 3 | **Sample Size:** N=40    **Refusal Rate:**   - 26% (37/141) of interested individuals declined to participate or had no further contact/interest; - 28% (40/141) of interested individuals were randomised to condition.   **Reasons for Refusal:** N/A    **Participation:**  Overall 95% (38/40) of participants completed the allocated condition; 95% (19/20) of each condition completed allocated condition.  **Dropout reasons**: N/A | **Quality of Life:**   - No statistically significant changes in QOL - Fatigue and fatigue improved but not significant (both p=0.06)   **Positive effect:**  Increased physical  **Negative effect:**  All adverse events were mild; no serious events reported  **Cost-Effectiveness:** N/A | **Setting:**   - multi-site, national pilot - Successfully implemented across diverse locations   **Provider/staff:**   - Accredited Exercise Physiologist (7 years’ experience), provided telehealth consultations and SMS support - High engagement by lead EP (consults, emails, texts); no major barriers reported | **Cost:**  Not directly reported; time burden tracked (consults, calls, messages**)**  **Enablers:**   - Personalisation - Multiple communication channels - Autonomy in module completion   **Barriers:**   - Drop-off in tracking module completion after week 2 - Low use of expert Q&A feature - Exercise boredom, usability challenges for some | **N/A** | - Small sample size; underpowered for some outcomes - No long-term follow-up - Generalizability limited by self-selected, mostly urban, tech-literate participants - Usability just below benchmark | - ExerciseGuide was highly acceptable & moderately usable for men with metastatic PCa. - Participants preferred personalized content, supportive structure, & flexibility, though additional variety & ongoing contact may further enhance engagement. |
| Feldman-Stewart 2018  **Country**: Canada  **Design**: Observational  **Aim**:  Evaluate how Canadian patients use & perceive a widely available online DA for early-stage PCa | **Population:**  Canadian patients with early-stage PCa who voluntarily accessed the DA  **Sample Size:**   - Website visitors (N= 2,210) - DA users (N=993) - Evaluation respondents (N=168)   **Age: N/A**  **Stage:** Early    **Treatment:** N/A | **Intervention name**: N/A  **Supportive Care Type:**  Online patient DA supporting VCEs & treatment decision-making for early PCa  **Mode of delivery:**  Website  **Duration:** N/A | **Preference measures:**   - Usage - Effectiveness   **Preference tools:**   - Google Analytics (for tracking website usage - Usability Assessment   **QoL measures:**  N/A | - High rates of component use, especially early modules - DA regarded as helpful (M = 4.1/5) - Participants “Would recommend” (M = 4.2/5) - Users found the PtDA helpful regardless of how many components were used | **Sample Size:** N=168    **Refusal Rate:** ~85% of DA users did not complete usability assessment    **Reasons for Refusal:**  N/A  **Participation:** Not applicable (voluntary use) | **QoL:**  N/A  **Positive Effects:**   - High user satisfaction (*M* ≥4 on core items) - Demonstrated usefulness across diverse preferences for support   **Negative Effects:** N/A  **Cost-Effectiveness:** N/A | **Setting:**   - Online platform accessed nationally in Canada - Widely available and promoted by developer & linked from relevant databases   **Provider/Staff:**   - No clinical staff required to deliver or facilitate PtDA; patients independently accessed program. - Research staff involved in the initial design and usability assessment - Staff adoption N/R | **Cost:** N/A  **Enablers:**   - No login/account required - Modular design allowing variable depth of use - Privacy preserved to minimise barriers - Designed to accommodate individual preferences for decision-making support   **Barriers:**   - Users did not view core educational content (52%) - Social media not a source of traffic - Limited demographic or contextual information collected | **Organizational/Staff Level:** Maintained online with increasing usage; not dependent on staff   - **Individual level:** Continued growth in usage across provinces (e.g., 8x increase from Sept 2016 to Sept 2017) | - No demographic or clinical data collected from (anonymous) users - Unable to determine population-level effectiveness (e.g., decision regret, treatment outcomes) - Usability data may reflect a self-selected subset of more engaged users | PtDA rated as helpful & recommended by users, regardless of how many components were used, supporting its flexibility in accommodating diverse decision-support preferences in early-stage PCa patients. |
| Feng 2023  **Country**: Canada  **Design**: Observational prospective cohort  **Aim**: Assess feasibility & acceptability of remote symptom monitoring in older adults with metastatic PCa | **Population:**  Older adults with metastatic PCa initiating treatment  **Sample Size:** N=90  **Age:** *M=*77 yrs (range 65-91yrs)  **Stage:** Metastatic  **Treatment:**   - Chemotherapy (n=34) - Androgen therapy (n=43) - Radium-223 (n=13)   **Other:**   - ≥College (76%) - Reside with others (78%) - Retired (86%) - Frailty (VES-13 ≥ 3): (58%) - ECOG 0–1 (90%) - Bone metastasis (76%) - Comfortable with smartphone (59%), Internet (59%) | **Intervention name**: N/A  **Supportive Care Type:** Remote symptom monitoring including tracking (daily & weekly) & triggered alerts  **Mode of delivery:** Online (computer or phone)  **Duration:** 3–4 weeks (1 treatment cycle) | **Preference measures:**   - Feasibility - Acceptability   **Preference tools:**   - Satisfaction Questionnaire   interviews  **QoL measures:**   - Edmonton Symptom Assessment Scale (ESAS) - Weekly validated tools for pain, fatigue, mood, anxiety and insomnia - Triggered questionnaires (participants who reported high scores on ESAS) | - High satisfaction - 38% preferred phone-based RSM (38%) - Benefits: daily reflection, motivation, health awareness, psychological support - Challenges: questionnaire fatigue, digital literacy barriers, timing/logistics - Suggestions: reduce frequency (weekly vs daily), simplify questions, clarify scales, include more physical symptom details | **Sample Size**: N=90    **Refusal Rate:** 26% (33/129) eligible participants declined to participate.    **Reasons for Refusal:**   - Not interested (n=17) - Overwhelmed (n=11) - Too invasive (n=2) - Low tech literacy (n=2) - Too busy (n=1).   **Participation:** 94% (90/96) of enrolled participants completed the study.  **Dropout reasons: N/A** | **QoL:**   - Some participants reported daily ESAS helped keep track of symptoms (improving and worsening)   **Positive Effects:**   - Reported perceived benefits (66%) - 210 severe symptom alerts prompted clinical responses   **Negative Effects:**   - daily reporting burdensome (8%) - Some technological & survey content issues   **Cost-Effectiveness:** N/A | **Setting:**   - Two tertiary cancer centres - Successfully embedded into routine care during recruitment   **Provider/Staff:**   - Research coordinators (data collection, alert triage) & oncologists, nurses (follow-up) - Symptom alerts acknowledged by clinical team (97%) - Follow-up actions included medication changes, referrals, or ED advice | **Cost:** N/A  **Enablers:**   - Flexibility in mode (web/phone) - Integration with clinical team - High patient motivation & engagement   **Barriers:**   - Digital literacy for some - Length & repetitiveness of surveys - Timing of survey delivery (emails late in the day) | **Organisational level:**  Not studied beyond one treatment cycle  **Individual level:** Follow-up limited to 3–4 weeks; long-term adherence N/A | - No control group - Single-cycle duration limits long-term insight - Volunteer bias likely (tech-literate, motivated participants - Burden from daily reporting for some participant - Triggered alert system limited to weekdays/working hours | Remote symptom monitoring was feasible & acceptable for older adults with metastatic PCa. Preferences varied by modality, with a substantial portion favouring phone-based support. Participants valued regular health engagement & personalization, but preferred simplified & less frequent symptom assessments. |
| Finlay 2020  **Country**: Australia  **Design**: Three-arm RCT  **Aim**: Examine how website architecture (free-choice vs. tunnelled) effects engagement, usability, & acceptability of a web-based computer-tailored physical activity intervention for PCa survivors. | **Population:**  Post-treatment PCa survivors with localised or locally advanced disease  **Sample Size:** N=71   - Free choice (n=27) - Tunnelled (n= 27) - Control (n=17)   **Age:** *M*=66.6 yrs (SD = 9.66; range = 38–91)  **Stage:**   - Stage I: 11.7% (n=9) - Stage II: 32.5% (n=25) - Stage III: 45.5% (n=35)   **Treatment:**   - Surgery (79%) - RT (35%) - HT (17%) - Surveillance (7%)   **Other:**   - Married/partnered (84.5%) - High school (81%) - Retired (53.5%) - Employed (40%) - Resided in major cities (45.5%), inner regional (27.3%), outer regional (15.6%) | **Intervention name**:  Prostate Cancer Health & Fitness (PCHF)  **Supportive Care Type:**  Physical activity promotion via online tailored behavioural modules released weekly (tunnelled), all at once (free choice), or non-tailored links to static resources (control).  **Mode of delivery:** Website  **Duration:** 4 weeks | **Preference measures:**   - Engagement - Usage   **Preference tools**:   - SUS - E-health Engagement Scale (12-item) - Perceived Relevance Scale (3-item) - Website usage data (e.g., logs completed, module access) - qualitative feedback   **QoL measures:** N/A | - Tunnelled condition scored highest in usability, perceived relevance and engagement - Users appreciated autonomy, but many preferred guidance - Mixed feedback on applicability of content - Tunnelled group completed more physical activity logs (M = 2.6 vs 1.5; p = 0.01) - Free-choice participants accessed more “once-off” content - Free-choice participants more often accessed social support & habit formation modules (p < 0.01) - Recommendations included tailored resistance training, clearer content, & mobile app adaptation | **Sample Size:** N=71    **Refusal Rate:**   - 0% (0/156 individuals assessed for eligibility refused participation - 50% of those (78/156) were eligible, registered, & were randomised.   **Reasons for Refusal:** N/A  **Participation:**   - Overall: 72% - Free choice: 65% - Tunnelled: 76% - Control: 78%   **Dropout reasons:**   - not completing baseline - lost interest - health concerns | **QoL:** N/A  **Positive Effects:** General increase in PA across all groups  **Negative Effects:** Low engagement with self-monitoring in free-choice group  **Cost-Effectiveness: N/A** | **Setting:**   - National online intervention - Successfully implemented, though recruitment target not met   **Provider/Staff:**   - Behavioural scientist, exercise physiologist supported “Ask an Expert” feature - Limited direct interaction; low usage of “Ask an Expert” feature | **Cost:** N/A  **Enablers:**   - Autonomy-supportive content - Tailored advice with self-monitoring tools   **Barriers:**   - Low engagement with interactive features - Perceived content irrelevance for active participants | **N/A** | - Underpowered; did not meet recruitment target - High dropout in free-choice arm - Self-reported outcomes only - High baseline activity may have limited intervention impact - Limited tailoring for demographic/clinical subgroups | Website architecture influenced how PCa survivors engaged with a physical activity intervention. While many preferred autonomy, tunnelled formats encouraged greater engagement with self-monitoring tools, suggesting that guided approaches may better support behaviour tracking. |
| Galvão 2017  **Country**: Australia  **Design:** Two-arm RCT  **Aim**: Evaluate effectiveness of peer-led, phone-based, multimodal self-management intervention on exercise participation, psychological distress, & QoL in men with LPCa | **Population:**  Men with LPCa  Sample Size: N=463   - Intervention (n= 232) - Usual care (n=231**)**   **Age =** *M=*64.4 yrs (SD=7.7); yrs  **Stage:** LPCa  **Treatment:**   - RP (70.4%) - EBRT + ADT (12.5%) - EBRT (3.5%) - Brachytherapy ± ADT (6.5%) - ADT (2.4%) - Other/mixed (2.8%)   **Other:**   - Married/partnered (84.5%) - ≥High school (62%) - *M*=10.8 months (SD=3.0) post-diagnosis - *M*=7.3 months (SD=3.0) post-treatment | **Intervention name**: N/A  **Supportive Care Type:** Peer-led telephone-based group sessions with self-management & exercise promotion  **Mode of delivery:**  Phone   - Online - Printed materials - Exercise equipment (e.g., heartrate monitor)   **Duration:** Monthly 60 min teleconference/6 months | **Preference measures:**   - Participation - Feedback   **Preference tools:**   - Website usage metrics - Attendance rates at sessions - Qualitative feedback   **QoL measures:**   - Brief Symptom Inventory (BSI) for psychological distress - AQoL-8D for general QoL - EPIC & International Prostate Symptom Score (IPSS) for disease-specific QoL | *Participation*   - Session attendance median = 4/6 - 28% accessed website (median 1 visit, avg. time = 23 min)   *Feedback*   - Positive perception of peer support & self-management tools - Some valued resistance training element - Suggested benefit for rural/remote participants due to accessibility | **Sample Size:** N=463    **Refusal Rate:**  Of patients sent study information, 53% (801/1501) refused participation or did not respond; 31% (463/1501) were eligible & enrolled in the study.    **Reasons for Refusal: N/A**    **Participation:**   - Overall: 82% (381/463) enrolled men completed the allocated 6-mo condition. - Intervention: 79% (183/232). - Usual care: 86% (198/231).   **Dropout reasons: N/A** | **QoL:**   - AQoL-8D Relationship subscale improved at 3 months in intervention ( =.038) - At 12 months: significant group differences for Mental Superdimension, Happiness, Coping, Self-Worth subscales   **Psychological Distress:**   - Some improvements (BSI global score interaction p .038), but no sustained or clinically significant differences - **Positive Effects:** Resistance Exercise: Intervention group significantly higher resistance exercise at 3 months (+19.4 min/week, p=.003) & 6 months (+14.6 min/week, p=.027) - Significant improvement in proportion meeting sufficient activity guidelines at 3 months (18% INT vs. 7.8% UC, p = .003)   **Negative Effects**: N/A  **Cost-Effectiveness:** N/A | **Setting:**   - Statewide with phone-based delivery - Implemented across multiple hospitals and locations   **Provider/Staff:**   - Trained peer support volunteers & Nurse counsellor overseeing & responding to medical questions - Peer leaders effectively delivered the intervention; professional oversight ensured fidelity | **Cost: N/A**  **Enablers:**   - Peer support - Group-based, remote delivery - Provision of exercise tools   **Barriers:**   - Website underutilized - Decline in resistance exercise post-intervention suggests sustainability challenge   **Other:**   - Peer leaders received substantial training & support - Remote access enabled inclusion of rural participants | **Organizational/Staff Level:**  Not maintained beyond the 6-month intervention period  **Individual level:**   - Gains in resistance exercise & activity adherence not sustained at 12 months - 81.6% completed 12-month follow-up | - No objective exercise measures (self-report only) - No measure of fatigue - Website engagement low - Short intervention duration may limit long-term adherence | A peer-led, telephone-based intervention increased resistance exercise & adherence to activity guidelines in the short-to-medium term. However, sustained improvements & engagement beyond 6 months may require extended contact or alternative delivery methods to maintain motivation & usability. |
| Glajchen 1996  **Country**: USA  **Design**: Evaluation  **Aim**: Evaluate effectiveness of a teleconferenced educational intervention | **Population:**  Men with advanced PCa  **Sample Size:** N=107  **Age:** Range 40–89 yrs  yrs  **Stage:** Advanced  **Treatment:**  N/A | **Intervention name**: N/A  **Supportive Care Type:** Educational psycho-oncology workshop on advanced PCa & pain  **Mode of delivery:** Phone teleconference  **Duration:** 1 hour (25 min urologist education, 25 min social worker psychosocial & pain management, 10 min Q&A) | **Preference measures:**   - Satisfaction   **Preference tools:**   - Posttest knowledge recall assessment on treatment & pain - Satisfaction Survey - Qualitative feedback   **QoL measures:** N/A | *Satisfaction (n=40)*   - Workshop useful (88%). - Information helpful (95%). - more encouraged about prognosis (95%). - Preferred features: anonymity, strontium info, pain management clarity - Suggested improvements: longer Q&A, fewer statistics, availability of audiotape | **Sample Size:** N=107    **Refusal rate & reasons:** N/A | **QoL: N/A**  **Positive Effects:**   - High recall of key messages on pain, treatment options - Increased hope & perceived support - Increased knowledge about advanced PCa   **Negative Effects:**   - Some respondents disliked hearing negative statistics   **Cost-Effectiveness:** $15–$20 per participant (teleconference operator); potentially lower if self-hosted | **Setting:**   - Remote phone-delivery - Easily delivered from presenters’ offices; program deemed replicable   **Provider/Staff:**   - Urologist (MD) for medical education & social worker for psychosocial/pain management - Interdisciplinary collaboration effective | **Cost:** $15–$20 per person (operator-facilitated call); cost-effective at scale  **Enablers:**   - Anonymity, home-based access - Low barrier to participation (no travel, free) - Targeted information for older men   **Barriers:**   - Low return rate for follow-up tools - No pre-post comparison possible - Limitations for hearing-impaired or non-English speakers not addressed   **Other:**  Recommendations included mailing questions in advance & using audiotapes or the internet in future | **N/A** | - No pretest to assess knowledge gains - Low follow-up response rates - No clinical outcomes measured - Results may reflect a self-selected, motivated sample | Participants highly valued the anonymity, accessibility, & focused information provided by the teleconference. They expressed strong preference for the interactive format & content on pain management, with requests for longer sessions & broader dissemination (e.g., audiotapes or online versions). |
| Goode 2022  **Country**: USA  **Design**:  Two-arm RCT  **Aim**: Test effectiveness of a perioperative behavioural training program delivered via mobile telehealth (mHealth) to reduce urinary incontinence after RP. | **Population:**  Men undergoing RP for PCa  **Sample Size:** N=245   - Intervention: 123 - Control: 122   **Age:** *M=*62 yrs  **Stage:**  N/A  **Other**  White (control 67%; intervention 77%) | **Intervention name**: N/A  **Supportive Care Type:**  Daily mobile behavioural training with pelvic floor muscle training (PFMT), bladder control techniques, & self-monitoring  **Mode of delivery:** mHealth  **Duration:**   - 1–4 weeks pre-op + 8 weeks post-op - Total duration: 9–12 weeks - daily 5–10 min mobile sessions | **Preference measure s:**   - Adherence - Satisfaction   **Preference tools:**   - Incontinence Impact Questionnaire ICIQ-Short Form (primary outcome: time to continence) - EPIC-UI - Incontinence Impact Questionnaire Short Form (IIQ-SF) - IPSS QoL - Global Patient Ratings (perceived improvement, satisfaction) - Activity restriction, pad use, return to work/normal activity - Adherence monitoring via mHealth   **QoL measures: N/A** | *Adherence:*  ≥80% of sessions completed: 80% (intervention), 84% (control)  *Satisfaction:*   - No significant difference in satisfaction or global perception of improvement between groups - Some evidence that daily interactive education (even in control) was perceived as helpful - Engagement supported by real-life stories, visual aids, & “fun facts” for personalization & motivation - Alerts triggered nurse outreach for non-engagement or learning difficulties | **Sample Size:** N=245    **Refusal Rate:**   - 11% (37/325) assessed for eligibility declined to participate; - 75% (245/325) were randomised into study.     **Reasons for Refusal: N/A**.    **Participation:**   - Overall: 71% (175/245) - Intervention: 73% (90/123) - Control: 70% (85/122). - **Dropout reasons** not starting intervention - declining to continue, health issues. | **Positive Effects:**   - Continence improvement > 12 months in both groups   **Negative Effects:**   - No added benefit from behavioural training over education-only mHealth in continence (ICIQ, EPIC-UI, IIQ-SF, pad use), satisfaction, return to work and resumption of normal activity   **Cost-Effectiveness:** N/A | **Setting:**   - U.S. Veterans Affairs medical centres (n=3) & university-affiliated urology practices - Successfully deployed across sites using mHealth platforms   **Provider/Staff:**   - Nurse practitioners responded to alerts - Behavioural scientists, urologists, and a multidisciplinary team developed the program - Limited direct interaction; nurse engagement triggered only for low adherence | **Cost: N/A**  **Enablers:**   - Mobile format accessible anytime - Customizable branching logic - Real-time alerts & nurse backup - Functional back-end support for monitoring, customization, & engagement   **Barriers:**   - Lack of in-person feedback or hands-on training for PFMT - Possible contamination: some control participants accessed PFMT elsewhere - Lower-than-expected incontinence rates reduced effect size - No significant benefit in those with high adherence vs. low adherence | **Organizational/Staff Level:** N/A post-study  **Individual level: Participants:** Follow-up to 12 months showed no sustained group differences | - No comparison to usual care or in-person training - Potential contamination of control group (outside PFMT access) - High baseline knowledge/access may have diluted intervention effect - Limited interactivity compared to in-person therapy - Self-report measures & dropout rates limit generalizability | Although well adhered to, the mHealth-delivered behavioural training program did not improve continence outcomes over a general prostate education mHealth program. Participants were engaged with both formats, suggesting that remote delivery is acceptable, but more interactive or in-person methods may be needed for greater impact. |
| Hallward et al. 2020  **Country**:  Canada  **Design**: Qualitative  **Aim**: Explore how participants  describe their experiences using behaviour change techniques (BCTs) while enrolled behaviour changes technique program | **Population**: PCa survivors &caregiver partners.  **Sample**: N=46   - Patients (n=19) - Caregivers (n=17) - Dyads (n=19)   *Note: only patients reported below*.  **Age**:  M= 64.7yrs (SD=7.22, range 40-70+)   - **Treatment**: Surgery (63%) - Chemotherapy (21%) - RT (53%) - HT (37%) - Brachytherapy (11%) - Watchful waiting (11%) - Other (5%) - Stage: - Early (58%) - Advanced (32%) - Unknown (26%)   **Other**:   - English speaking (84%) - Married (89%) - Post-secondary (37%) - University (32%) - income >$100,000 (37%) - 1–3 yrs post-treatment (52%) | **Intervention name**:  TEMPO: A tailored, self-management BCT program.  **Supportive care type**:  Psychosocial & physical activity self-management & behaviour change program, using 5 modules:  (1) dyadic needs assessment; (2) Goal setting & action planning; (3) Coping; (4)  sources of support & motivational tools; & (5) past successes &  post-intervention.  Also included library on areas including making treatment decisions, dealing with stress & worry, or fitness & health.  **Delivery mode**:  Website  **Duration**: 10 weeks with modules released every 2 weeks. | **Preference measures**:   - Engagement   **Preference tools:**   - Qualitative interviews - Use & discussion of BCTs.   **QoL Measures:** N/A | Generally positive experience of the program, with some reporting benefits from/in:   - Learning about behaviour change techniques (BCTs), such as proper (SMARTT) goal setting to ensure goals are achievable, & their beneficial use in PCa support context. - Reinforcement of previous learnings of BCTs, & usefulness in doing module worksheets & activities. - Resistance to BCTs: some felt the BCTs were unnecessary, not relevant, too much for them to manage, or they were already familiar with them as a concept. | **Sample size**: N=19 dyads (n=19 PC).  **Refusal rate & reasons: N/A**.  **Participation**: 100% | **QoL:** N/A  **Positive effects**:   - Realised value of social support & help seeking - Relationship strengthening - Engagement with physical activity - Improved self-efficacy, confidence, & positive self-talk. - Normalising symptoms & challenges.   **Negative effect**: Some founds certain BCTs unnecessary or too much to manage.  **Cost-effectiveness**: N/A. | **Setting:**   - Community and outpatient clinics - successfully used in homes.   **Provider/Staff:**   - Delivered entirely by research staff; no clinicians needed | **Cost**: N/A.  **Enablers**: Participants provided with pedometers & resistance bands. Provided with worksheets to assist implementing BCTs in modules.  **Barriers**: Discussions revolved around mandatory modules, may suggest non-mandatory modules were not utilised. BCTs which were less thoroughly taught/ emphasised were less likely to be adopted. Complexity of making multiple behaviour changes may also represent a considerable challenge to those already facing the burdens associated with cancer. | N/A | - Data primarily relate to dyads - Sample are PCS in relationship - Convenience sample with high levels of education, socioeconomic status, & motivation | - The program provided valuable support & facilitated behaviour change for PCa survivors & their caregivers. - Dyads experienced positive outcomes from using BCTs, although some resisted full engagement. - The program's structured approach & focus on both physical & psychological health were appreciated by participants. |
| Hälleberget al. 2017  #2186  **Country**:  Sweden  **Design**: Qualitative  **Aim**:  Explore how patients with PCa perceived participation with or without  support of smartphone app during radiotherapy | **Population**: PCa patient receiving adjuvant RT  **Sample**: N=28  Intervention (n=17) Control (n=11)  **Age**:   - Overall range 57 -77 yrs - Intervention M=70 yrs (range 63-76 yrs) - Control M=70 yrs (range 57-76 yrs)   **Treatment**  Intervention   - RT EBRT (35%) - Brachytherapy + EBRT (65%)   Control   - RT EBRT (18%) - Brachytherapy + EBRT (82%)   **Stage:** N/A  **Other**:  Intervention   - Married/partnered (70%) - Rural/suburban residence (53%) - ≥University (47%) - Retired (72%) - Adjuvant HT (71%)   Control   - Married/partnered (82%) - Urban residence (64%) - Junior compulsory education (55%) - Retired (91%) - Adjuvant HT(64%) | **Intervention name**:  Interaktor.  **Supportive care type**:  Symptom reporting, risk assessment model, evidence-based  self-care advice, along with the provision of immediate access to clinicians  **Delivery mode**:  Smartphone app  **Duration**: N/A | **Preference measure(s)**:   - Participation   **Preference Tools:**   - Patient-reported outcome measures (PROM) - Open-Ended Interviews - Content analysis of experiences with app   **QoL Tools:** N/A | - High engagement with the app, patients reported symptoms daily. - Patients who used app described it as facilitating factor, especially for mutual participation. | N=28 *(purposive sampling used)*  **Refusal**: 12% (4/32) declined to participate.  **Refusal reasons**: N/A  **Participation**: 100% | **QoL**: N/A.  *Note: Intervention reported below*  **Positive effects**:   - Perceived participation when reporting symptoms in app & when they received dialogue from nurse about how to resolve problem - felt active, took their own initiatives, & had e opportunity to express their problems & concerns - experienced greater ease in collecting prescriptions when they were from reporting symptoms in the app - self-care advice & weblinks promoted participation in care.   **Negative effect**: N/A.  **Cost-effectiveness**: N/A. | **Setting:**   - university hospitals (n=2, one rural & one urban). - app integrated into care process in intervention group   **Provider/ Staff:**   - Contact Nurses (primary point of contact for patients); Radiotherapy staff (delivered radiation treatments but had limited capacity to answer broader health questions); study-specific nurses (monitored symptom alerts via the app and contacted patients when alerts were triggered).   Nurses acknowledged app facilitated real-time communication & improved timely responses to patient needs. | **Cost**: N/A  **Enablers**:   - Provided with a smartphone with app installed - Clear instructions - technical support - immediate response from nurses.   **Barriers**: | N/A | - Predetermined analysis scheme may have influenced data interpretation.   Distribution of educational level & Gleason scores differed between groups. | The app was regularly utilised & facilitated mutual participation & provided a sense of security by ensuring immediate responses to patient needs. |
| Handley et al. 2022  **Country**: USA  **Design**: Single-arm pilot feasibility  **Aim**: Assess feasibility of digital health coaching for men with PCa | **Population**: Men with a history of PCa requiring active treatment in past 2 yrs  **Sample**: N=88  **Age**: *M=*68.9 (SD=7.8).  **Treatment**   - Active surveillance (16%) - Radiation (16%) - Radiation + ADT (26%) - ADT alone (7%) - Surgery (16%) - ADT + NAAD (17%) - ADT + chemotherapy (2%)   **Stage**:   - I (11%) - II (41%) - III (18%) - IV (23%) - Unknown (7%)   **Other**:   - non-Hispanic White (95%) - retired (64.9%) - married (83.6%) - public insurance (61.4%) | **Intervention name**: N/A  **Supportive care type**:  Digital health coaching program, with a designated health coach/advisor (HA) & e-modules around illness management & healthy lifestyle behaviours.  Content included: fatigue, pain management, healthy  eating, exercise, managing incontinence, sexual health,  managing stress & anxiety, financial toxicity, goal setting  during treatment, managing side effects, communicating  with the health care team, & medication adherence. Communication frequency & mode based on participant preference.  **Delivery mode**:  Digital health platform  phone calls  digital nudges (text, email, or app)  **Duration**: 12 weeks, weekly telephone calls  ≤4 digital nudges/week | **Preference measures**:   - Retainment - Engagement (time spent in calls, volume of opened content, inbound communications to HA)   **Preference tools:**   - Cancer Behaviour Inventory (CBI) version 3 - Expanded PCa Index Composite for Clinical Practice (EPIC-CP) - COmprehensive Score for financial Toxicity (COST)   **QoL measures:**   - Patient-Reported Outcomes Measurement Information System (PROMIS) Global Health Scale | *Engagement:*   - On average, participants completed 12.1 calls, with a total call time of 153 min (SD 106.9), consisting of 1.5 initiated from the participant, & 10.6 initiated from the HA. - average of 13.5 e-mails most of which were HA initiated (11.0) - Average50.3 SMSs most of which were HA initiated (34.6) & online sessions (18.7). | **Sample Size:** N=88    **Refusal Rate:** Not explicitly reported however 91% (1008/1108) of individuals approached did not consent.    **Reasons for Refusal: N/A**.    **Participation:** 72% (63/88) of enrolled individuals completed the intervention.  **Dropout reasons:** N/A | **QoL**:   - Significant improvements at 3 months in hormonal - symptom score, irritation obstruction score, urinary function score, & urinary incontinence score, - Nonsignificant improvements in bowel symptom score & sexual symptom score - Physiologic & psychosocial well-being did not significantly change.   **Positive effects**: Self-efficacy & financial toxicity both significantly improved at 3-months.  **Negative effect**: N/A  **Cost-effectiveness**: N/A. | **Setting:**   - cancer centre. - Fully remote delivery model   **Provider/Staff:**   - Health coaching delivered by external team of 4 certified advisors - No burden on medical oncology or nursing staff. | **Cost**: N/A.  **Enablers**: Engagement centred on establishing small, measurable health goals established by the participant in collaboration with their HA. HA communication frequency & mode was based on participant preference. Opportunities  were available for both structured or ad lib interaction between  visits using digital engagement techniques.  **Barriers**: N/A | N/A | - Majority recruited using targeted e-mailing, which may have increased selection bias. - participants were largely white, healthy, & educated. - Participants required access to a smart device/ computer. - Acceptability was not formally evaluated. | Digital health coaching for men with PCa was feasible in this study. |
| Hawkins et al. 2017  **Country**: USA  **Design**: Three-arm RCT  **Aim**:  Determine whether combining computer-based support with a mentor would benefit PCa patients more than either intervention alone. | **Population**: Newly diagnosed PCa patients.  **Sample**: N=310   - CHESS (n=103) - Mentor only (n=105) - CHESS+ (n=102)   **Age**: *M*=60 yrs  **Treatment**:  Surgery: 3% (n=9)  **Stage**: 1 & 2  **Other**: ~42-47 days post-diagnosis | **Intervention name**:  Comprehensive Health Enhancement Support System (CHESS)  **Supportive care type**:   - CHESS: online resource providing information, social support, & interactive tools - Mentor: ongoing contact with mentor - CHESS+: CHESS and mentor   **Delivery mode**:   - CHESS: website - Mentor: email & phone - CHESS+: Website, email, phone   **Duration**: 6 months.   - CHESS & CHESS+: Weekly phone calls from mentor in first month, twice in second month, & once monthly in final 4 months - average 12 min /call, longer initial calls | **Preference measures**: Engagement  **Preference tools:**   - EPIC - Functional Assessment of Cancer Therapy – Prostate (FACT-P)^24^   **QoL measures:**   - World Health Organization’s Quality of Life (WHOQOL) measure | - Of CHESS conditions 22% (n=43/198) never logged in - Of Mentor conditions, 8% (n=16) did not complete 2 calls, & were defined as nonusers, i.e., 92% engaged in Mentors. - About 10%-15% in the Mentor conditions-initiated calls to the mentor for additional support; 15% of scheduled calls took place as e-mail exchanges. - average number of full calls or e-mails was 6.4 per participant. | **Sample Size:** N=310    **Refusal Rate:** 32% (147/461) invited individuals declined to participate.  **Reasons for Refusal:**   - Not a computer person not interested - did not return pretest, - overwhelmed   too busy    **Participation**:   - Overall: 97% (301/310). - Mentor only: 97% (102/105) - CHESS: 96% (99/103) - CHESS+Mentor: 98% (100/102). | **QoL**:   - Improved functional well-being, positive coping, & bonding in the combined (CHESS+) condition at different time points, inconsistently. - Trends towards Mentor only group having better outcomes than CHESS only group.   **Positive effects**: N/A.  **Negative effect**: N/A.  **Cost-effectiveness**: computer-based system may be preferable due to cost | **Setting:**  cancer centres (n=3)  Home-based internet access support  **Staff:**   - Mentors were Cancer Information Service specialists. Included experienced mentor who received extra training and a male mentor with experience in the Man-to-Man program. - No burden on clinical staff. | **Cost**: N/A  **Enablers**:   - internet access & loan computers - training on using Google search. - mentor resources. - Alternative for email instead of calls   **Barriers**:   - Patient frustration & overwhelm by the volume of information - preferring to focus only on what was most relevant - some calls missed because of scheduling difficulties - some participants stopped last few calls. | **Organisational level:** N/A.  **Individual level:**   - Modest 6-month effects for QoL, emotional wellbeing, cancer information competence, cancer-patient functioning, - Higher effects for functional wellbeing & positive coping over time. | - Recruitment may have biased towards people with lower computer ability or digital literacy, or fewer comorbidities. - No baseline measures reported (i.e. only 6-weeks, 3-months, & 6-months). | - Modest benefit of combined condition was lower than anticipated. - Combining human & computer-based interventions did not produce the expected much stronger benefits to patients. Given the costs involved, the computer-based system alone is likely preferable. |
| Heneka et al. 2023  **Country**: Australia  **Design**:  Qualitative  **Aim**: Assess acceptability of a nurse‐led PCa survivorship  intervention adapted for virtual delivery & tailored to post‐surgical care, in a  regional Australian hospital & health service. | **Population**: Men newly diagnosed with LPCa, scheduled to undergo, or had undergone, radical or robotic prostatectomy surgery  **Sample**: N=22   - PCa patients (n=16) - HCP & stakeholders (n=6)   Note: patients reported below  **Age**: *M=* 64 yrs.  **Treatment**: Undergone or scheduled for prostatectomy  **Stage**: N/A | **Intervention name**: N/A  **Supportive care type**:  Virtual PCa survivorship care program, including teleconferencing, with modules related to  post‐operative care & symptom management; psychoeducation.  stress management & coping flexibility; conjoint couple coping,  problem solving & goal setting for the future.  **Delivery mode**: Website  **Duration**: 12‐weeks | - **Preference measures**: Affective attitude - Intervention coherence - Self-efficacy - Burden - Opportunity costs - Ethicality - Perceived effectiveness   **Preference tools:**   - Semi-structured interviews using Theoretical Framework of Acceptability (TFA) constructs   **QoL tools:** N/A | *Note: PCa patients reported below*   - High acceptability, convenience, cost saving, & improved quality of care - Affective attitude: positive feelings, especially prospect of regular HCP contact, information access, & normalising recovery. Virtual format enabled discussion of sensitive issues. - Intervention coherence: program created strong sense of support. - Self-efficacy: - Confidence in utilising & navigating technology. Greater convenience than attending FtF - Burden: Low perceived burden - Opportunity costs: No perceived sacrifice of care quality delivered virtually - Ethicality: program aligned with men’s values - Perceived effectiveness: High self-reported symptom management & engagement with HCP. | **Sample Size:** N=22    **Refusal Rate: N/A**  **Participation: N/A** | **QoL**:   - N/A   **Positive effects**: Participants felt active in recovery & empowered to discuss PCa   - improvements in care coordination, timely identification of needs, & overall care quality.   High satisfaction & convenience  **Negative effect**: N/A  **Cost-effectiveness**: Virtual engagement more cost-effectiveness than in person HCP | **Setting:**   - Regional Hospital and Health Service - Fully remote program   **Provider/Staff:**   - Specialist virtual care nurse leading program with minimal additional training - High staff confidence due to existing virtual care experience - High perceived self-efficacy and support for program delivery | **Cost**: N/A   - **Enablers**: Integrated tablet with videoconferencing, resource access, remote monitoring tools, & self-management book. - Safe environment to seek support and use technology. - Incremental access to virtual care nurse - Program previously tested with expert & consumer consensus.   **Barriers**: Difficulty with written resources; preference for audio/visual formats.  Need for a more user-friendly tech platform. | **N/A** | - Internet access in the study region met the needs but may not be the case in other regions - Participants were all in the early phases of PCa care with timely access to the virtual care program (within 3 months post‐operatively). | - Virtual post-surgical care delivered via videoconferencing is highly acceptable to PCa survivors in regional Australia. - Men with PCa often given large amounts of pre-operative information. These findings suggest a preference to receiving information in smaller amounts, as needed, & when they were ready. |
| Heneka et al. 2024  **Country**: Australia  **Design**:  Quasi-experimental  pre-  post-pilot  **Aim**:  Determine acceptability & feasibility of post-surgical nurse-led virtual PCa survivorship intervention in a rural Australian hospital & health service. | **Population**: Men newly diagnosed with LPCa, scheduled to undergo, or had undergone, radical or robotic prostatectomy surgery  **Sample**: N=17  **Age**: *M*= 64.0 yrs (range 56-72 yrs)  **Treatment**: N/A  **Stage**: N/A  **Other**:   - Resided regionally/rurally (53.0%) - Australian-born (58.8%) - Aboriginal or Torres Strait Islander (18%), - English speaking (100%) - Partnered (64.7%) - Lived with spouse/partner (58.8%) - concession card (64.7%) - Income below national median (53%) | **Intervention name**: N/A  **Supportive care type**:  Virtual PCa survivorship care program tailored to t post-operative  recovery journey  (targeting  care, psychoeducation, problem-solving  & goal setting),  **Delivery mode**: Website  **Duration**: 12‐weeks | **Preference measures**:   - acceptability   **Preference tools:**   - Acceptability of Intervention Measure (AIM) - Intervention Appropriateness Measure (IAM) - Feasibility of Intervention Measure (FIM) - eHealth Literacy Scale (eHEALS) - EPIC - Godin-Shephard   Leisure-Time Physical Activity Questionnaire (GSLTPAQ)  **QoL measures:**   - Health-related quality of life (HRQoL) - Brief Fatigue Inventory (BFI) | All measures showed very acceptability (≥4/5) (AIM), appropriateness (IAM) & feasibility (FIM) at baseline, with measures increasing as participants worked through the program. | **Sample Size:** N=17    **Refusal Rate:** 39% (12/31) declined participating in the program.  **Reasons for Refusal:**   - did not require additional support - hesitant about using technology or study procedures.   **Participation:** N/A | .  **QoL**:   - trend for HRQoL to increase over time. - At 3-mo follow-up, significantly reduced distress, & problems related to urinary function, pain, uncertainty about   future, & work.  **Positive effects**: N/A  **Negative effect**: Increase in mean global fatigue over time.  **Cost-effectiveness**: Health resource utilisation cost per participant averaged $438 for outpatient  care & $7446 for hospitalisation | **Setting:**   - rural hospital and health service - Infrastructure already in place; implementation highly feasible.   **Staff:**   - Specialist virtual care nurse. - High staff confidence in virtual care delivery. - Low additional burden due to structured manuals and dedicated virtual care roles. - High perceived self-efficacy and support for program delivery. | **Cost**: N/A  **Enablers**:   - program ran in parallel with follow up - delivered by healthcare team - received self-management resource addressing key PCa-related challenges - low-intensity model underpinned by CBT - Regular videoconferencing - dedicated tablet for access - skilled virtual care nurse.   **Adaptations**: N/A.  **Barriers**: Impact of COVID-19 on surgery scheduling | N/A | - High e-health literacy in participants may not be representative of other regions - Small sample size may not have detected effects, & limits generalisability. | A tailored, nurse-led virtual care program was acceptable & feasible for rural PCa survivors. |
| Huber et al. 2018  **Country**: Germany  **Design**:  Cross-sectional comparison  **Aim**: Compare  users of traditional FtF vs online support groups | **Population**: PCa patients  **Sample**: N= 1641   - FtF (n=955) - Online (n=686)   **Age**:   - FtF *M=*72 yrs - Online *M*=65 yrs   **Treatment**:  *FtF*   - Surgery: (74% - RT (28%) - Other (5%) - Deferred treatment (6%) - Systemic (25%)   Online   - Surgery (68%) - RT: (39%) - Other (7%) - Deferred (8%) - Systemic (29%)   **Stage**: N/A  **Other**:   - Online reported higher education, income, and time since diagnosis - FtF comprised more retirees | **Intervention name**: N/A  **Supportive care type**:  Support group  **Delivery mode**: FtF or online.  **Duration**: N/A | **Preference measures**:   - Helpfulness in decision-making   **Preference tools:**   - Patient Health Questionnaire (PHQ-2)   **QoL measures:**   - EORTC QLQ-C30 - EORTC QLQ-PR25 (prostate specific QoL) - Generalized Anxiety - Disorder Scale (GAD-2) - distress   thermometer | Majority of participants rated support groups as helpful for making treatment decision (a little 15%, moderately 18%, & very helpful 34%) | **Sample Size:** N= 1,641    **Refusal Rate:** ~71% for FtF & 76% for Online (29% (971/3330) & 24% (814/3357) survey return rates)  **Reasons for Refusal:** N/A    **Participation:** N/A | **QoL**:   - Global health status slightly lower in FtF than OSG - FtF reported significantly higher symptom scores & lower function scores than OSG - Online had higher sexual activity scores than FtF - Distress significantly less frequent in FtF - Assessment of survivor concerns, depression & anxiety symptoms did not differ between groups.   **Positive effects**: FtF reported consistently higher agreement across all positive benefits than OSG  **Negative effect**: Online significantly less in control, less content with themselves, & less understanding of illness  **Cost-effectiveness**: N/A. | **Setting:**   - Regional support groups (n=230) - existed parallel to standard medical care; no integration with hospitals/clinical pathways. Both modalities adopted by users.   **Provider/Staff:**   - Trained volunteer group leaders (FtF); self-moderated (OSG). - FtF time burden absorbed by trained group leaders; OSG had minimal workload implications | N/A | N/A | - Patient self-report - Unspecified participation and refusal rates | - Both modalities of peer support received very positive ratings from their users & had a significant impact on treatment decision-making. - F2FSG reported consistently higher agreement across all positive benefits received from the support group, than OSG**.** |
| Huber et al. 2024  **Country**: Germany  **Design**: Single-arm evaluation  **Aim**: Evaluate nationwide online DA for patients with non-metastatic PCa from perspective of patients & urologists | **Population**: Patients with non-metastatic PCa & urologists  **Sample**:   - Patients (n=11,290) - Urologists (n=194)   **Age**: Median=67 yrs (range=39-90 yrs).  **Treatment**: N/A.  **Stage**:  **Clinical T stage**   - cT1a/b: 10.6% (n=1182) - cT1c: 49.5% (n=5495) - cT2a: 15.3% (n=1695) - cT2b: 8.6% (n=950) - cT2c: 12.2% (n=1350) - cT3: 3.6% (n=404) - cT4: 0.3% (n=35)   **Clinical N stage**   - cN0/x: 98.9% (n=10,663) - cN1: 1.1% (n=119)   **Clinical M stage**   - cM0/x: 99.3% (n=10,645) - cM1: 0.7% (n=78)   **Other**:   - partnered (90%) - German speaking (92%) - A-level education (36%) - public health insurance (74%) - middle income bracket (57%). - Time since diagnosis (median 4 wks; range 3-7 wks) | **Intervention name**: Entscheidungshilfe Prostatakrebs    **Supportive care type**: DA offering guideline-based content, utilising 17 educational videos with an attending physician (actor), & tool which collects health-related data to provide personalised information.  **Delivery mode**: Online platform with educational videos & personalised information.  **Duration**: N/A | **Preference measures**:   - Satisfaction   **Preference tools:**   - Acceptability E-scale - IIEF - Lee Comorbidity Index - IPSS   **QoL tools:**   - Patient Health Questionnaire (PHQ-4) | - 87.7% of users - reported high satisfaction (≥80% of maximum score). - DA satisfaction positively associated with treatment satisfaction, &: younger age (≤ 60), less distress, high knowledge, higher income, lower oncological risk, & normal erectile function.   59% of patients used the tool together   - with their partner, 33.1% alone, 5.1% with their children, & 2.4% with other relatives or friends. | **Sample Size:** n=11,290 (PCa only)    **Refusal Rate: N/A**    **Participation: N/A**. | **QoL**: N/A.  **Positive effects**: *(Urologist reported advantages of using DA*)   - Better-informed patients (85%) - Higher patient satisfaction (76%) - Better involvement of relatives (63%) - Benefits in terms of documentation (45%)   **Negative effect**: N/A.  **Cost-effectiveness**: Half of urologists  reported time savings (49%), 31%  reported no change, & 20% reported more time  required | **Setting:**   - Urology clinics - DA introduced into routine clinical pathways during diagnosis/ treatment consultations   **Provider/staff:**   - Urologists distributed DA access cards and entered basic patient data to personalise tool - 47% of urologists implemented DA in practice. High satisfaction & recommendation (92%). - Minimal additional time burden | **Cost**: Previous testing of tool development (reported elsewhere).  **Enablers**:   - DA content personalised according to information provided - German urologists can order access cards for their patients free of charge.   **Barriers**: N/A | N/A | - Positive effects & cost-effectiveness only asked of urologists, not PCa patients. - features of German healthcare system limit generalisability | - Patients & urologists report high levels of acceptance & satisfaction with the online DA. - The DA supports shared decision-making & may improve the adoption of active surveillance & watchful waiting. |
| Hughes et al. 2020  **Country**: UK  **Design**:  Mixed-method randomised feasibility trial  **Aim**:  Determine practicality of delivering cancer support intervention for active surveillance | **Population**: Low or intermediate risk PCa patients on active surveillance for <12 months.  **Sample**: N=13  Intervention (n=10)  TAU (n=3)  **Age**:  *M=*66.4 yrs (range 52-76 yrs)  **Treatment**: N/A.  **Stage**: N/A   - Other: - ≥College (46%) - retired (77%) - White(85%) - Married (69%). - Time since treatment *M=*3.7 months (range: 1-11). | **Intervention name**:   - PROACTIVE, vs - TAU   **Supportive care type**: psycho-educational support intervention for PCa patients managed on Active Surveillance, composed of two components: group workshops (Overview of active surveillance, stress management & breathing-based relaxation techniques, &, wives & partners) & internet-delivered information modules (6 modules: Active Surveillance & physical activity, food & stress, relaxation & resilience, talking to others, thoughts & feelings, daily life, money & work)  **Delivery mode:**   - Online modules - FtF workshop   **Duration**: 6 weeks (modules weekly; 3x2 hour group workshops) | - **Preference measures**: Attendance - Acceptability   **Preference tools:**   - Questionnaires - Semi-structured interviews   **QoL measures:**   - HADS - Warwickshire/Edinburgh Mental Wellbeing Scale (WEMWBS) - MAX-PC | - Workshops highly attended & acceptable (92%); 4–6 participants ideal; QoL workshop especially positive. - Online modules had lower engagement, used mainly as support; barriers included mild symptoms, limited time, & low internet use. - Goal setting unpopular; many didn’t see a need/disliked concept. - Participation often driven by desire to “give back” due to feeling ineligible for treatment. - Participants appreciated access to resources for future use, even if currently not needed. - Outcome measures felt irrelevant due to focus on more severe symptoms than participants experienced. | **Sample Size:** N=13    **Refusal Rate:**   - 45% (58/128) declined to participate. - Only 16% of eligible participants (13/80) agreed to participate.   **Reasons for Refusal:**   - Lack of time (n=12) - too busy (n=8) - no value in participating (n=6) - carer responsibilities (n=4).   **Participation:**   - 100% of randomised participants (N=13/13) received allocated condition | **QoL**: no significant positive impact on diet or lifestyle behaviours, nor anxiety or depression.  **Positive effects**:   - Reassurance from social connection & hearing others’ stories - Received clarity on previously contradictory advice & recommendations (i.e. feeling better informed), leading to greater feeling of security being on active surveillance.   **Negative effect**: N/A  **Cost-effectiveness**: N/A | **Setting:**   - PCa centres (n=2) - intervention offered alongside usual care; not embedded into routine services.   **Provider/Staff:**   - Two Clinical Nurse Specialists (CNS) facilitated in-person workshops - Research staff provided logistical support, including recruitment & study monitoring. - No substantial role changes for CNS facilitators | **Cost**: N/A.  **Enablers**: Expertise of Clinical Nurse Specialists.  **Barriers**: Low perceived need for intervention, which made people feel like ‘imposters’ in the intervention, despite 62% of participants having clinical levels of depression. | N/A | - Low recruitment rate. - Participants' self-perceived low levels of anxiety. - Inadequate participation to collect quantitative data or draw significant conclusions. | - Interest in this intervention, & engagement with internet content was low, due to low perceived need. - Perception that their cancer was non-severe led to low engagement in intervention content, & made people feel like ‘imposters’ in the intervention, despite 62% of participants having clinical levels of depression. - Implies that many men on active surveillance may not be aware of the need for supportive care, even when mood symptoms indicate need for it. |
| Ilie 2020  **Country**: Canada  **Design**: Feasibility study  **Aim**: To evaluate the implementation of a Prostate Cancer Patient Empowerment Program. | **Population:** Non-metastatic PCa survivors  **Sample Size**: n=30  **Age**: M = 68.9 yrs (range: 56-83 yrs)  **Stage**:  **Treatment**:   - Active surveillance (13%) - RP (33%) - RT (3%) - ADT (7%) - RT+HT (17%) - RP+HT (13%) - RP+RT+HT (13%)   **Other:**   - White (93%) - University educated (67%) - Married (90%) - Retired (67%) - >25 months since diagnosis (73%) - Income >$100,000 CAD (40%) | **Intervention name**: Prostate  Cancer–Patient Empowerment Program (PC-PEP)  **Supportive Care Type:** Mindfulness-based stress reduction, intimacy and connection training, a buddy system where participants were encouraged to call two participant buddies each week to check in on them, individually tailored aerobic exercise and strength program, pelvic floor training.  **Mode of delivery:** Online (daily 2-min videos delivered via email), phone (buddy phone calls), videos (pelvic floor training and reminder)    **Duration:** 28 days | **Preference measures:**   - Interest in overall program - Perceived benefit and important of program (if provided at diagnosis) - Personal usefulness - Recommendation likelihood - Adherence (Weekly compliance with each program component)   **Preference Tools:**   - Weekly surveys - End of program survey (1-10 Likert scale) - Exit focus groups   **QoL measures:**   - Kessler Psychological Distress scale (K10) | - Overall program evaluation (9.61/10) and interest in program (8.89/10) were very high. - Components *rated extremely useful:* science behind PC-PEP, pelvic floor, and physical activity training. Daily video messaging reminders (9.61/10). - *Components rated very useful:* meditation (7.46/10) and intimacy/connection training. - Compliance was consistent over intervention. - Likelihood to recommend the program to someone who had a PCa diagnosis was extremely high (9.79/10). - Very high exercise compliance (6.9 days a week, average 280 min per week. - High pelvic floor exercise compliance (68% met target; average of 5.3 days or 115 min a week) - Medium mindfulness compliance (average 5 days weekly of daily target; 95% met weekly 70min target) | **Sample size**: N=30  **Refusal rate: N/R**  **Participation**: 100% (no dropout)  **Dropout reasons:**   - N/A | **QoL:** Significant improvements in mild to severe psychological distress from baseline (27%) to post-test (7%).  **Positive effects**: Increased social engagement through buddy system.  **Negative effect**: N/A  **Cost-effectiveness**: N/A | **Setting:**   - A university and local support groups - Successfully piloted   **Provider/Staff:**   - Psychologist (mindfulness training); psychology professor (intimacy coaching); physiologist (fitness assessments, exercise guidance); physiotherapist (pelvic floor muscle training - HCP involved in delivery were highly trained and specialized. | **Cost:** N/A  **Enablers:** High perceived competence of Clinical. Daily video messages & reminders. Multidimensional intervention with comprehensive support.  **Barriers:** N/A | N/A |  | This program was feasible, with overall high evaluation ratings. |
| Ilie et al. 2023  **Country**:  Canada  **Design**: Crossover RCT  **Aim**: Examine effects of online home-based  physical, mental, & social support intervention  on preventing psychological distress among men  undergoing curative PCa treatment | **Population**: men  scheduled for curative PCa surgery or RT ± HT  **Sample**: N=128   - Intervention (n=66) - Control (n=62).   **Age**: median= 66 yrs (range 60-70 yrs).  **Treatment**:   - ADT (41%) - RP (44%) - Radiation (41%) - RT (salvage) (15%)   **Stage**: N/A  Other:   - White (91%) - Partnered (89%) - ≥University (47%) | **Intervention name**: PC-PEP  **Supportive care type**: comprehensive physical, mental, & social support intervention providing education, patient activation, & empowerment on healthy living including physical & mental health, dietary recommendations, social support,  physical & pelvic floor fitness, stress reduction using a biofeedback device, social  connection & intimacy, & optional social support. Included daily emails with 3-5 min video resources and option of calling two coparticipants weekly & joining a live monthly Zoom videoconference  of all participants in the intervention.  **Delivery mode**:   - Online - Phone (peer support calls)   **Duration**: 6 months. | **Preference measure(s)**:   - Participation - Interest in intervention - Recommend ability - Perceived usefulness of program components   **Preference Tools:**   - Quantitative Exit survey - Semi-structured interview - Weekly compliance surveys   **QoL Tools:**   - Kessler Psychological Distress Scale (K10) | - High interest in PC-PEP both at recruitment (8.6/10) and after the study (8.8/10). - Perceived usefulness rated highly at baseline (8.9/10) and overall (8.5/10).   *Most useful components:*   - PVT videos (8.2) - Exercise materials (7.9) - Dietary advice (7.8) - Educational content (7.8) - Daily educational/empowerment videos (7.6)   *Moderately useful:*   - Intimacy resources & text reminders (7.2) - Monthly Zoom sessions (7.0) - Website (6.7) - Stress reduction device (6.5)   *Less useful:*   - Meditation videos (5.9) - Buddy system (5.3) - Mentor system (4.9) | **Sample Size:** N=128    **Refusal Rate:** 16% (28/171) declined to participate.  **Reasons for Refusal:**  N/A  **Participation**:   - Overall, 99% (N=139/140) of randomised participants completed allocate condition - **Dropout reasons:** N/A | **QoL**: Significant reduction in psychological distress, depression, & anxiety in intervention group compared to control  **Positive effects**: Improved mental health, reducing need for clinical psychological treatment.  **Negative effect**: N/A  **Cost-effectiveness**: cost-effective, with an estimated cost of 200 CAD per patient | **Setting:**   - University tertiary care centre - Minimal impact on workloads   **Provider/Staff:**   - specialist virtual care nurse. - PC-PEP did not appear to burden clinicians or nurses | **Cost**: 200 CAD per patient.  **Enablers**: High perceived competence of Clinical team. Daily video messages & reminders. Comprehensive support.  **Barriers**: Time & effort-intensive intervention | **Organisational level:** Program being tested in phase 4 implementation trial throughout Canada & New Zealand, but maintenance N/R |  | The PC-PEP intervention had high success, with high completion rate, positive impacts on outcomes, & high ratings of user experience across its various components. |
| Ilie et al. 2023  **Country**: Canada  **Design**: Qualitative  **Aim**:  Explore perceptions of patients regarding online  patient empowerment program | **Population**: Men with history of non-metastatic PCa  **Sample**: N=30  **Age**: *M* = 68.9 yrs (range 47–88 yrs)  **Treatment**:   - Active surveillance (13%) - RP (47%) - RT ± HT (20%) - RP+RT+HT (13%) - ADT (7%)   **Stage**: N/A  **Other**:   - White (93%) - University educated (67%) - Partnered (100%) - Retired (70%) - >25 months since diagnosis (73%) - Income >$80,000 CAD (73%) | **Intervention name**: PCa-Patient Empowerment Program (PC-PEP)  **Supportive care type**:  Patient education & empowerment videos, prescribed physical activities (including PF exercises), a mostly plant-based diet, stress reduction techniques, intimacy  education, social connection. To foster social support, participants can opt to be paired with two “buddies” undergoing  similar treatment for their diagnosis), & support (activities encompass sleep, stress reduction techniques such as meditation, deep breathing, & recalling positive emotions, & daily exercise routines.  **Delivery mode**:  Online  **Duration**: 28 days | **Preference measure(s)**:   - Feedback - Perceived usefulness   **Preference Tools:**   - semi-structured exit interviews - Quantitative exit surveys   **QoL Tools:**   - Optional social support network (‘buddy system’) | *Usefulness*   - Overall Usefulness: Rated 9.0/10 - Suggested integration from diagnosis stage (9.6/10)   Experience:   - Universally found valuable and beneficial - Buddy system praised, especially for remote/rural participants - Intimacy content resonated with younger patients - Expert support highly valued during treatment   *Timing & Duration*   - Preferred Start: From time of diagnosis - Program Length: 28 days acceptable; many suggested follow-ups or extension to 3–6 months   *Component Ratings*   - (Pre/Post Range: 8.55–9.73 / 7.46–9.89) - Recommendation to Others: 9.8 - Post-Completion Interest: 8.9 - Early Access Importance: 8.5–9.6 - Daily Videos & Emails: 9.2 - Pelvic Floor & Physical Activity: 8.8 - Intimacy & Connection: 8.0 - Meditation: 7.5 | N=30  **Refusal & reasons**: N/A  **Participation**: N/A | **QoL**:   - Improved continence. - Improved mental & physical health, reduced psychological distress, enhanced quality of life   **Positive effects**:   - Contact with other participants reduced loneliness and provided support, especially for those grieving sexual function loss after RP. - program boosted confidence, self-efficacy, and helped bridge the gap between hospital care and long-term psychosocial needs from diagnosis onward.   **Negative effect**: N/A.  **Cost-effectiveness**: N/A | **Setting:**  Home-based intervention.  No changes to EHR systems  **Provider/Staff:**   - Program’s daily videos led & delivered by a scientist in PCa QoL research and a radiation oncologist. - Staff adoption not reported | **Cost**: N/A.  **Enablers:**   - Online, home-based approach aligned with patient lifestyles and routines. - Weekly surveys increased accountability. - Buddy system enhanced connections and adherence. - Daily encouragement and achievable goals - Clear Kegel instructions (improved incontinence - Competent team   **Barriers**:   - Work, weather, and personal circumstances hindered participation, especially affecting buddy system. - Sharing personal experiences uncomfortable for some participants. | **N/A** | - Potential self-selection bias - Interviews conducted by program leads, which might have led to positive bias | - Patients highly valued the PC-PEP program & endorsed its integration into standard care from day one of diagnosis. - The program effectively addressed various survivorship needs, improved mental & physical health, & enhanced quality of life. - Further research is needed to explore the long-term impact & potential for broader implementation. |
| Inman et al. 2011  **Country**: USA  **Design**: RCT  **Aim**: Determine if additional phone  follow-up educational intervention  after surgery increases  understanding of managing  care at home & utilisation of fewer healthcare resources vs follow up | **Population**: Post-operative men who underwent RP for PCa  **Sample**: N=60   - Control (n=30) - Intervention (n=30)   **Age**:   - Intervention *M=*62.3yrs (range 48-75 yrs) - Control *M=*60.2 yrs (range 48-73)   **Treatment**: RP  **Stage**: N/A  **Other**: White (≥90%). | **Intervention name**: N/A  **Supportive care type**: Individualized reinforcement of  education via a phone call 3-5 days post hospital discharge.  **Delivery mode**: Phone  **Duration**: 1 phone call, *M* time <10 min. | **Preference measure(s)**:   - Helpfulness of receiving a phone call - Increased understanding   **Preference Tools:**   - Care Transitions Measure (CTM-3) - Individual reinforcement of education via telephone call 3-5 days post hospital discharge   **QoL Tools:**  N/A | 97% in the intervention group found phone call helpful | **Sample Size:** N=60    **Refusal Rate:** N/A.  **Reasons for Refusal: N/A**.  **Participation:** N/A | **QoL**   - Improved understanding of post-operative care (intervention group).   **Positive Effects:**   - Intervention used fewer unplanned resources (47%) vs. control (60%): clinic visits, physician calls, ER visits, re-hospitalizations.   **Negative Effect:**   - No difference in self-rated understanding of health management. - **Cost-effectiveness:** Reduced unplanned resource use, potential cost savings. | **Setting:**   - Academic medical centre - Successfully implemented parallel to usual care.   **Provider/Staff:**   - Experienced urology nurses conducted phone calls. - Intervention considered highly feasible with minimal impact on workload given reduced unplanned follow-up calls/visits | **Cost**: Less than 10 min per call/patient.  **Enablers**:   - Timing received well - individualized reinforcement of education - experienced nurse   **Barriers**: N/A. | N/A | - Potential influence of patient characteristics not examined - Differences in surgical approach (robotic assisted vs. open procedures) not fully accounted for | Timely follow-up conversation with patients increased recall & may be an effective method to assist patients in recalling information. The telephone call intervention strategy was reported by patients as helpful & reduced the need to utilize other resources. |
| Jensen et al. 2011  **Country**: Denmark  **Design**: Prospective RCT  **Aim**:  Investigate  if nurse-led phone consultations optimise resources & secure rehabilitation & patient  satisfaction post-surgery | **Population**: Men with PCa referred for RP by either RRP or RALP.  **Sample**: N=95   - Intervention (n=46) - Control (n=49)   **Age**:   - Intervention *M=* 64.1 yrs - Control *M=*62.5 yrs   **Treatment**: RP  **Stage**: N/A.  **Other**: Reside with spouse (intervention 97.6%; control 88.5%) | **Intervention name**: N/A  **Supportive care type**: Additional phone consultation 3 days post-op by a CNS  **Delivery mode**: Phone  **Duration**: **standardstandard**1 phone call, ~15 min (prolonged in special need cases) | **Preference measure(s)**:   - Patient satisfaction - Sense of security   **Preference Tools:**   - Questionnaires   **QoL Tools:**   - Visual Analogue Scale (VAS) | No significant difference in overall satisfaction between the intervention & control | **Sample Size:** N=95    **Refusal Rate:** 33% (n=47/142) of individuals declined to enrol or were not eligible.  **Reasons for Refusal:** NR  **Participation**: 100% (N=95/95) of individuals who were allocated to conditions completed it. | **QoL**: Some benefits in some symptoms (e.g. bowel function), however this was minimal across measures.  **Positive effects**: N/A.  **Negative effect**:   - No difference in overall efficacy of patient satisfaction, sense of security & post-operative discomfort. - no difference in need of post-operative contact to other health care affiliates.   **Cost-effectiveness**: N/A. | **Setting:**   - Urology medical centre. - Intervention tested as quality improvement but not adopted into routine care   **Provider/Staff:**   - Two CNS - feasible within existing staff workloads without requiring new hires/ dedicated personnel. | N/A. | N/A |  | Patients expressed high satisfaction & sense of security regardless of TC. TCs will be considered an additional service rather than a **standard** procedure. |
| Johnson et al. 2016  **Country**: USA  **Design**:  Pilot cohort  **Aim**:  Evaluate benefit of novel decision-making application for men with newly diagnosed localized PCa & investigate associations between patient preferences & treatment choice | **Population**: Men with newly diagnosed PCa  **Sample**: N=109  **Age**: *M=* 62.5yrs (range 44-78yrs)  **Treatment**: N/A  **Stage**: N/A  **Other**:   - White (92%) - Married/partnered (>81%) - ≥College (67%) - Income ≥$100,000 (43%) | **Intervention name** WiserCare  **Supportive care type**:  Application providing education, preference measurement & personalized decision analysis, used before initial PCa consultation to form basis of shared decision making.  **Delivery mode**:  Online.  **Duration**: N/A. | **Preference measure(s)**:   - Satisfaction   **Preference Tools**:   - DCS - Shared Decision making (SDM) survey - WiserCare Preference Assessment   **QoL Tools:** N/A | - Overall satisfaction high (91.2%) - no significant differences in SCC scores between intervention & control | **Sample Size:** N=109  **Refusal Rate:** 13% (n=18/143) of invited participants declined to enrol.  **Reasons for Refusal:** N/A (self-selection)  **Participation:** 87% (109/125) of those opening the link to survey completed the module.  **Dropout reasons:** N/A | **QoL**: N/A  **Positive effects**:   - Significant improvements in all DCS subdomains (informed, values clarity, support, uncertainty, effective decision making). - More intervention participants felt included in & responsible for treatment decisions compared to usual care participants   **Negative effect**: N/A.  **Cost-effectiveness**: N/A. | **Setting:**   - Urology centre - implemented as part of quality improvement initiative. Integration was practical and feasible, as the tool did not require changes to existing clinical workflows or IT systems.   **Provider/Staff**:   - Urological oncologists (n=5) participated in implementation - HCP voluntarily participated in implementation of pilot and were educated about report | **Cost**: N/A.  **Enablers**: feasible web-based intervention with personalized decision analysis  **Barriers**:   - Potential survey fatigue - clinical flow issues   **Adaptations**: non-specific adaptations to improve clinical flow of post-consultation survey engagement.  **Other**: N/A | N/A | - Low participation in post-consult survey (30%) - Predominance of Caucasian, affluent, married patients | - Implementation of the WiserCare application was associated with decreased decisional conflict & enhanced shared decision making for men with newly diagnosed LPCa. - There was no difference in overall satisfaction with cancer care between control & intervention groups. |
| Kenfield et al. 2019  **Country**:  USA  **Design**:  Pilot RCT  **Aim**:  Determine feasibility & acceptability of digital lifestyle intervention  among men with PCa | **Population**: Men with clinical stage T1–T3a PCa within 5 yrs of diagnosis & > 3 months post-treatment  **Sample**: N=76   - Intervention (n=37) - Control (standard care; n=39)   **Age**: *M=*65 yrs (range 51–79 yrs)   - Intervention median 66 yrs - Control median 65 yrs   *Note: intervention group reported below*  Treatment:   - RP (43%) - Active surveillance (57%)   **Stage**:   - T1 (57%) - T2 (38%) - T3a (5%)   **Other**:   - White (91%) - Graduate school (43%) - Married (78%). | **Intervention name:** Prostate 8  **Supportive care type**: Digital lifestyle intervention of physical activity, healthy eating, smoking cessation, & support based on personalised recommendations, emphasized goal setting/planning, securing social support, enhancing self-efficacy, making healthy  behaviours enjoyable, staying motivated, harms/benefits, & reinforcing adoption  of the recommendation.  **Delivery mode**:   - Website ( lifestyle report & community member support) - Fitbit One - Phone (4-5 text messages weekly - Email (Fortnightly, lifestyle topics & recipes.   **Duration**: 12 weeks. | **Preference measure(s)**:   - Satisfaction - Feasibility - Acceptability   **Preference Tools:**   - Online questionnaires - Fitbit One Activity Tracker   **QoL Tools:**   - Health-related quality of life assessment (no specific questionnaire used) | *Satisfaction:*   - Website quality: 60.7% high/very high - Fitbit ratings: 87.1% good/excellent - Text messaging: 68.8% good/excellent - Baseline personalized report: 78.1% good/excellent - Website: 60% - Fitbits: 90.6% - Text messaging: 73.3% - Recipes key feature influencing continued website use   *Website*   - Median of 3 visits over 12 weeks - Homepage blog: unnoticed by 46% - Recipe of the week: unnoticed by 25% - Shopping guides: unnoticed by 50% - Workouts (weight room/home-based): unnoticed by 44% | **Sample Size:** N=76.    **Refusal Rate**: 22% (50/228) declined to participate.  **Refusal Reasons**: Unwilling to travel.  **Participation**: Overall: 84% (64/76) of enrolled participants completed study;  Intervention: 86% (32/37) completed  Control: 82% (32/39) completed | **QoL**: no difference in change in QOL measures between intervention or control groups for nearly all measures  **Positive effects**:   - Increased intake: cooked tomatoes, cruciferous vegetables, fish; reduced intake processed meat - Increased steps and general activity (not significant in vigorous activity) - No significant changes in anthropometric measurements or biomarkers within 12 weeks   **Negative effect**: physical activity-related adverse events (joint pain, muscle pain) in intervention group  **Cost-effectiveness**: N/A. | **Setting:**   - Academic medical centre - Implemented as pilot with potential for integration into survivorship programs. No change to standard care pathways/ IT infrastructure.   **Provider/Staff:**   - Research staff onboarded participants, distributed Fitbits, assisted with technical issues, & collected outcome data. - No direct involvement of clinicians in intervention delivery - Research staff managed delivery; minimal additional workload since most intervention components were automated. | **Cost**: N/A.  **Enablers**:   - Personalized recommendations - use of technology.   **Barriers**: Participants requested clinical support for treatment management, online patient forums, and personalized exercise recommendations. | **Organisational level:** N/A.  **Individual level:** Behavioural effects largely persisted at 17-month follow-up for intervention arm (*suppl data*). | - Low website usage - Lack of diversity in sample | The digital health lifestyle intervention demonstrated high levels of participant satisfaction with specific components, particularly the Fitbits & text messaging. Despite low overall website usage, the personalized recommendation report & recipes were well-received, indicating that targeted, user-friendly features can enhance engagement & support the adoption of healthy behaviours among men with PCa. |
| Kenfield et al. 2021  **Country**: USA  **Design**: Three-arm pilot RCT  **Aim**:  Determine feasibility, safety, & acceptability of remotely  monitored exercise in men with PCa | **Population**: Men with metastatic  castrate-resistant  PCa  **Sample:** N=25.   - Aerobic intervention (n=8) - Resistance (n=7) - Control (n=10)   **Age**: Median 71 yrs (range 51-84 yrs)  **Treatment**:   - RP (52%) - RT (80%) - LHRH analogue/antagonist (100%) - Abiraterone (56%) - Enzalutamide (28%) - Other anti-androgen (92%) - CT (20%) - Sipuleucel-T (48%) - Other immunotherapy (4%) - Radium-223 (8%)   **Stage**: N/A  **Other**:   - Racial/ethnic minority (20%) - ≥University (84%) - Married/partnered (84%) - Median time diagnosis to enrolment 10.5 yrs (range 0.9-26.3yrs) | **Intervention name**:   - CHAMP: A Clinical trial of High-intensity Aerobic & resistance exercise for Metastatic Prostate cancer, vs TAU.   **Supportive care type**: Remotely monitored physical activity (high intensity aerobic and resistance exercise)  **Delivery mode**:   - HR monitor - Email - Phone   **Duration**:12 weeks. | **Preference measure(s)**:   - Attendance - Adherence - Tolerance - Acceptability   **Preference Tools:**   - Adherence Surveys - Tolerance Surveys - Adverse Events (AEs) Reports   **QoL Tools:**   - Surveys on lifestyle, diet and QoL | *Satisfaction*   - Satisfaction (very satisfied/satisfied): 90% - Program very good/excellent: 90% - Recommend to others: 90% - Overall rating: Excellent: 50%, Very good: 40%, Fair: 10%   *Adherence & Tolerance*   - Workouts attempted: 93% - Completed as prescribed or better (e.g., more reps): 87% - Median tolerance: Overall 6/10 (Resistance 7/10, Aerobic 5/10) - Attendance ≥70% of sessions: Resistance 100%, Aerobic 88% - Completed ≥70% as prescribed: Resistance 100%, Aerobic 63% - Completed ≥70% sessions (tolerance ≥5): Resistance 86%, Aerobic 75%   *Acceptability*   - Orientation and weekly calls rated 5/5 - 100% used heart rate monitor; usefulness 4/5 - 20% faced setup issues (e.g. HR monitor, facility access) - Local gym rated 4/5; 10% had attendance obstacles - Exercise ease rated low (2/5); high motivation and recall (5/5) - Remote session attendance rated 4/5 - Would attend onsite if remote unavailable | **Sample Size:** N=28; n=25 for remote intervention.    **Refusal Rate**:  6% (29/482) declined participation;, 95% (482/510) of those assessed for eligibility were not randomised.  **Reasons for Refusal:** N/A  **Participation**:   - Overall, 82% (23/28) of randomized participants completed the 12-week study. - Aerobic Group: 80% (n=8/10) completed the intervention & 12-w follow-up. - Resistance Group: 88% (n=7/8) completed the intervention & 12-w follow-up. - Control Group: 80% (n=8/10) completed the intervention & 12-w follow-up.   **Dropout reasons:**   - noncompliance - moved away - medical reasons - disliked testing | **QoL**: no meaningful changes in  predicted 24, 36, 48-month survival probabilities across arms.  **Positive effects**: Resting HR modestly reduced in resistance & aerobic  arms & modestly increased in the control arm.  **Negative effect**:   - Men reported no bone pain and low fatigue during exercise (aerobic median=4, resistance median=3). - No safety concerns identified. - Of 14 AEs, 8 occurred in resistance arm, mostly joint/bone pain consistent with disease; three were possibly study-related. - One aerobic participant reported hip/lower back pain; two resistance participants reported pain.   **Cost-effectiveness**: N/A. | **Setting:**   - Recruited from cancer centres (n=5) - content and coaching delivered remotely, using existing web platforms and wearable (Fitbit).   **Provider/Staff:**   - Exercise physiologists for remote monitoring & weekly check-ins - Health coaches adapted existing motivational interviewing skills to support PCa survivors specifically. | **Cost**: N/A  **Enablers**:   - Provided concierge service to locate local gyms; covered 3-month memberships; issued Polar H10 heart rate monitors for remote tracking; conducted short, helpful weekly check-ins. - 90% would not have participated if program was onsite only.   **Barriers**: Competing priorities (travel, life events, symptom progression, cancer recurrence) increased perceived difficulty. | N/A | Technology literacy in sample was high | Exercise completed with remote supervision by an EP was feasible, safe, & acceptable |
| Lambert et al. 2020  **Country**:  Canada  **Design**: Longitudinal qualitative  **Aim**:  Report on acceptability & usability of online psychosocial & physical activity self-management program to patients, caregivers, & HCP | **Population**: Men diagnosed with PCa in last 2 yrs, caregivers, & HCPs  **Sample**:   - PCa (n=19) - Caregivers (n=18) - HCPs (n=6).   *Note: PCa reported below*  **Age:** *M=*64.7 yrs  **Treatment**:   - Surgery (63.2%) - Chemotherapy (21.0%) - RT (52.6%) - HT (36.8%) - Brachytherapy (10.5%)   Watchful waiting (10.5%)  Other (5.3%)   - Stage: - Early (58%) - Advanced (32%) - Unknown (11%)   **Other**:   - English speaking (84%) - Married (89%) - Secondary school (37%) - University (32%) - <1 yr post-diagnosis (21%) - 1-3 yrs post diagnosis (52%) - 3-5 yrs post-diagnosis (26%) | **Intervention name**: TEMPO: a dyadic, Tailored, wEb-based, psychosocial & physical activity (PA) self-Management PrOgram.  **Supportive care type**: Psychosocial & PA self-management program for dyads with modules including: getting started (needs assessment); setting goals & making action plans; tracking progress; identifying & strengthening support systems; & (5) self-managing after TEMPO. In addition, included a health library incorporating 45 factsheets based on the most up-to-date evidence on self-management & PA.  **Delivery mode**:  Website  **Duration**:   - 10 weeks. - Modules released fortnightly | **Preference measure(s)**:   - Themes identified in thematic analysis   **Preference Tools:**   - Semi-structured qualitative interviews - User-tracking information - Physical Activity Readiness Questionnaire for Everyone (PAR-Q+)   QoL Tools:  N/A | - Overall experience of TEMPO was positive, with patients reporting it being easy, glad to be doing it. - TEMPO experienced as well done (i.e. well organised). - holistic aspect of TEMPO was praised. | **Sample Size:** n=19 patients    **Refusal Rate:** 51% (76/182) of dyads referred to study refused participation.  **Reasons for Refusal:** commitment (30%)  lost to follow-up (17%)  not interested (15%), overwhelmed (11%)  **Participation:** 70% (19/27) of consenting dyads completed study.  **Dropout reasons**: N/A | **QoL**:   - Reduced anxiety & better at managing emotions. - Increased or consolidated knowledge about self-management skills. - **Positive effects**: Improved discussions & communications with partners - Increased physical activity - Supported goal fulfilment   **Negative effect**: N/A.  **Cost-effectiveness**: N/A.  **Other**: Self-directed format was overall endorsed (reported in dyads only) | **Setting:**   - Recruited from cancer centres (n-4) and community-based channels. - Remote delivery; no integration into hospital systems or EHR.   **Provider/Staff:**   - No clinical staff required for delivery; research team and assistants managed all participant interactions - HCP expressed interest in recommending the program but noted it was not integrated into routine care. | **Cost**: N/A.  **Enablers**:   - Dyads set two SMARTT goals (≥1 physical activity-related goal) - Provided resistance bands, pedometers, and health library with 45 evidence-based factsheets. - Dyadic approach supported mutual coaching. Logical module sequencing.   **Barriers**:   - TEMPO was generic, lacked personalization - Common challenges included cancer-related issues, low motivation/energy, physical limitations, conflicting demands, weather, and limited time   **Adaptations**:  **Other**: Suggested improvements (across dyads): Reduce repetition, condense modules, tailor content to dyad needs, offer personalized exercise options, and include printable module materials. | N/A | - Required participation of a primary caregiver. - Much of the data were reported in dyads, only data by patients have been included - HCPs noted TEMPO limitations for subgroups: low health literacy, non-English speakers, elderly, low computer literacy, severely ill, or homeless. | program was acceptable & useful for PCa patients, providing benefits such as increased physical activity & enhanced communication. |
| Lambert et al. 2022  **Country**: Canada  **Design**: Parallel, two-group pilot RCT  **Aim**: Pilot online psychosocial & physical activity self-management program for men with PCa & caregivers | **Population**: Men with PCa who received treatment in past 2 yrs, with or without their caregivers  **Sample**:   - patient-caregiver dyads (n=33) - PCa patients (n=16) - Intervention (n=26) - Control Group (n = 23)   *Note: Men with PCa reported below*  **Intervention**  **Age**:   - Intervention (≤60 yrs 23.1%; ≥61 yrs 76.9%) - Control (≤60 yrs 26.1%; ≥61 yrs 73.9%)   **Treatment**:  *Intervention*   - Surgery (65.4%) - Chemotherapy (7.7%) - RT (50.0%) - HT (50.0%) - Brachytherapy (19.2%) - Other (19.2%)   *Control*   - Surgery (69.6%) - Chemotherapy (4.3%) - RT (21.7%) - HT (39.1%) - Brachytherapy (13.0%) - Other (13.0%)   **Stage**:  *Intervention*   - Early (61.5%) - Advanced (30.8%) - Unknown (7.7%)   Control   - Early (60.9%) - Advanced (39.1%)   **Other**:  *Intervention*   - English speaking (88.5%) - Married (84.6%) - Retired (57.7%) - 12–24 months post-diagnosis (61.5%)   *Control*   - English speaking (91.3%) - Married (95.7%) - Post-secondary school (43.5%) - Retired (60.9%) - 12–24 months post diagnosis (43.5%) | **Intervention name**: TEMPO: a dyadic, Tailored, wEb-based, psychosocial & physical activity (PA) self-Management PrOgram.  **Supportive care type**: Program to support learning self-management skills based on priority needs, goalsetting, build confidence to address & manage their need, & encourage PA.  **Delivery mode**: Website  **Duration**: 10 weeks. | **Preference measure(s)**:   - satisfaction   **Preference Tool**:   - SUS - Health Education Impact Questionnaire (heiQ v3.0) - International Physical Activity Questionnaire-Short Form (IPAQ-SF) - Multidimensional Self-Efficacy for Exercise Scale (MSES) - TEMPO Feedback Survey   **QoL Tools:**   - HADS - SF-12 - PSS - Dyadic Coping Inventory (DCI) | - Satisfaction Above average (75/100) - Behaviour change modules particularly useful. - Login frequency: 70% logged in ≥3 times; average duration 104 min (SD=129). - Module completion: Average 1.4/5 modules completed; adherence rate 45%. Most completed modules: #1 (60%), #2 (40%) - Worksheets most completed: unmet needs assessment (85%) & goal setting (60%) - Dyadic participation had larger mental health effect sizes compared to individual participation among men | **Sample Size:** N=52 men (n=33 in dyads, & n=16 individual men).    **Refusal Rate:** 26% (97/369) of individuals contacted  declined participation, & 46% (168/369) did not respond.  **Refusal reasons**   - No perceived need (27%) - no time (11%) - computer fatigue/no online interest (9%) addition to others   **Participation:** Overall, 90% (44/49) who were randomised completed intervention & 3-mo follow-up.  TEMPO: 81% (21/26).  Control: 100% (23/23).  **Dropout reasons**: N/A  . | **QoL**:   - Clinically significant anxiety reduction. - Mental QoL improved in 33% intervention vs. 26% control; no difference in physical QoL.   **Positive effects**: Improved knowledge, confidence, relationship strengthening, and increased physical activity.  **Negative effect**: N/A.  **Cost-effectiveness**: N/A. | **Setting:**   - Multi-centre implementation across cancer centres (n=6) - Offered in addition to standard clinical care pathways   **Staff:**   - Research assistants recruited & oversaw participants; Behavioural Scientists & Clinical Researchers developed content & managed program administration; HCP involved in content development - No clinical staff involved in delivery; participants self-guided through program | **Cost**: N/A  **Enablers**: User-friendly design, dyadic format, supportive RAs.  **Barriers**: lack of tailored content. | N/A |  | TEMPO found to be feasible & acceptable, with positive feedback on usability & engagement. The dyadic format was particularly beneficial for caregivers. |
| Lange et al. 2017  **Country**: Germany  **Design**: Quasi-experimental  **Aim**: Evaluate effectiveness, satisfaction, & acceptance of guided chat groups in psychosocial  aftercare for outpatients with PCa | **Population**: PCa patients  **Sample**: N=44   - intervention (n=18) - TAU (n=26)   **Age**   - Intervention *M*=60.5 yrs - Control *M*=62.8 yrs.   **Treatment**: Prostatectomy  **Stage**: N/A  **Other:**  *Intervention*   - Partnered (83%) - Grammar school (56%) - Employed (56%) - Residing in city or village (28% respectively) - Utilised psychosocial support outside of clinic (78%) - Time since prostatectomy to intervention M=5.3 months   *Control*  • Partnered (88%) • Grammar school (62%) • Employed (58%) • Residing in city (42%) • Utilised psychosocial support outside of clinic (35%) | **Intervention name**: N/A  **Supportive care type**: Five web-based chat-group sessions with psychotherapists and other intervention users vs TAU. Topics of conversation that were proposed by the therapist  were incontinence, FoP, partnership & sexuality after prostatectomy,  doctor-patient communication, occupational reintegration, resource orientation  & coping.  **Delivery mode**: Online/virtual chat room,  **Duration**:   - ~60-90 minute chats/week (total number unclear) | **Preference measure(s)**:   - Recommendation - Participate again   **Preference Tools:**   - Self-Developed Satisfaction and Acceptance Items - MAX-PC - Cancer Coping Questionnaire (CCQ)   **QoL Tools:**   - The Emotion Thermometers (ET-5) - SF-8 Health Survey | - one session/ week & 60 min/chat sufficient - Would recommend program: Agree (77%), Disagree (11%), Undecided (11%)   *Would participate again:*   - Agree: 65% - Disagree: 29% - Undecided: 6%   *Reasons for participating:*   - Contact with other patients (67%) - New information about PCa (61%) - Anonymous discussion of problems (50%) - Contact with therapist (39%) - Learning about chat/internet (17%) - Other reasons (11%) | **Sample Size:** N=44    **Refusal Rate:** 63% (241/384) refused or dropped out before participating in baseline.  **Reasons for Refusal:**   - Disinterested in the study: 33% (50/151) - Unable to use a computer: 21% (31/151) - Unable to use the internet: 25% (37/151) - Mental burden too severe: 9% (14/151) - Physical burden too severe: 4% (6/151) - other : 9% (13/151).   **Participation:**  31% (44/143) of those who commenced the study completed the study.  **Reasons for refusing intervention & choosing control arm:**   - Doubt intervention helpfulness (31%, 8/26) - not interested (31%, 8/26) - no time (19%, 5/26) - chat group impersonal (15%, 4/26) - privacy (12%, (3/26) - technical problems (8%, 2/26) - Other (19%, 5/26).   **Dropout reasons:**   - Followed five offered sessions: 33% (6/18) - Achieved goals: 17% (3/18) - Not helpful: 11% (2/18) - Number of sessions was limited: 11% (2/18) - No time: 6% (1/18) - Did not have serious problems: 6% (1/18) - Control their own problems: 6% (1/18). | **QoL**: No significant improvement for QoL measured.  **Positive effects**: Participants reported intervention worked as bridge between inpatient treatment & daily life.  **Negative effect**:   - Higher scores for anger in intervention group. - Intervention participants reported poorer results for primary & secondary outcomes in comparison to control group follow up. - All differences with at least a medium effect size favoured control   **Cost-effectiveness**:  22% (4/18) would pay for the chat program | **Setting:**   - urology outpatient clinics and cancer centres. - Setting adoption N/R   **Provider/Staff:**   - certified psychological psychotherapists - Minimal adaptation: HCP introduced & encouraged DA but were not required to facilitate its content | **Cost**: N/A.  **Enablers**:   - Personal contact between psychotherapist & intervention participants - technical assistance available.   **Barriers**: High need for cancer specific information.  **Adaptations**: N/A | N/A | - Participants could opt to be part of intervention or control group. - Differences in baseline scores & sociodemographic characteristics between intervention & control groups - Sample size lacked statistical power | - The study indicates that web-based chat groups may not be effective in decreasing perceived distress in PCa patients, despite participants' acceptance of the intervention. - Participants provided constructive feedback for improving the intervention, suggesting smaller groups & the inclusion of a physician. |
| Langius-Eklöf et al. 2017  **Country**: Sweden  **Design**:  Mixed method  **Aim**:  Investigate user behaviour, adherence to reporting, & patients’ experiences of using  Symptom management app during RT for localized advanced PCa | **Population**: men with locally advanced PCa planned for RT  **Sample**: N=66  **Age**: *M*=69 yrs (range 53-82yrs)  **Treatment**:   - Adjuvant Hormonal Therapy (76%) - EBRT (30%) - Brachytherapy + EBRT (70%)   **Stage**:   - Stage I (24%) - Stage II (44%) - Stage III (26%) - Missing (6%)   **Other**:   - Married/partnered (86%) - ≥University (50%) - Working or retired (89%) | **Intervention name**: N/A  **Supportive care type**:  Real time symptoms management including patients’ monitoring & reporting of symptoms, text alerts to HCP, & evidence-based self-care information & advice.  **Delivery mode**: Smartphone app  **Duration**:   - Daily reporting 5-8 weeks & for 3 weeks after - Reporting time ~5 min | **Preference measure(s)**:   - Adherence - Experience with app   **Preference Tools:**   - Qualitative interview - Logged symptom data (to measure adherence) - **QoL Tools:** N/A | - High adherence to daily symptoms reporting (87%). - Daily symptom reporting largely appreciated & gave a sense of safety & security by being seen, monitored, & prioritized by HCPs. | **Sample Size:** N=66    **Refusal Rate:**   - 32% (34/107) declined participation or could not be reached - 62% (66/107) who were eligible enrolled.   **Reasons for Refusal:** N/A  **Participation**: 95% (63/66) completed logged data throughout the study period.  **Dropout reasons**: N/A | **QoL**: not specified   - **Positive effects**: Increased sense of security - symptom reporting facilitated reflection on well-being - supportive for self-management of symptoms - self-care advice beneficial for information/ reducing symptom burden - Historical symptom graphs helped promote discussion with loved ones.   **Negative effect**:   - minority disliked being contacted by nurse in case of symptom alerts & some adjusted response to avoid contact - No significant technological issues reported however were most common at the start of reporting period requiring technical support.   **Cost-effectiveness**: N/A | **Setting:**   - university hospitals (n=2, one urban and one rural) - High patient engagement suggested feasibility for incorporation into standard care.   **Provider/Staff:**   - Registered nurses at oncology clinics monitored symptom alerts and responded to patient needs. - Staff adoption N/R | **Cost**: N/A.  **Enablers**:   - Patients were provided with a smartphone. - instructions how to use app & written checklist including a phone number for technical support. - Reminder notifications were sent at 3 PM if no report made. - Graphs to track symptoms were beneficial. - Symptom alerts helped manage care.   **Barriers**:   - Few technological problems - need for PIN login. | :  N/A |  | Patients with locally advanced PCa adhered to, appreciated, & faced few obstacles using an app for reporting & managing symptoms on a daily basis during radiotherapy. The app increased patients' reflections on their well-being & provided a sense of security, & thereby  served as a supportive tool for the self-management of symptoms during treatment of PC. |
| Lawen et al. 2024  **Country**:  Canada  **Design**: Crossover RCT  **Aim**:  Evaluate effectiveness of home-based patient empowerment program on urinary, bowel, sexual, & hormonal function in men PCa vs usual care | **Population**: Men  scheduled for/receiving curative PCa treatment  **Sample**: N=128   - Intervention (n=66) - Control (n=62)   Age   - Intervention median=66 yrs - Control median=68 yrs   Stage:  Intervention   - Stage I (1.5%) - Stage II (75%) - Stage III (23%)   Control   - Stage I (3.2%) - Stage II (67%) - Stage III (30%)   Treatment:  Intervention   - RP (44%) - RT (41%) - Salvage RT (15%) - ADT (41%)   Control   - RP (53%) - RT (44%) - Salvage RT (3.2%) - ADT (34%)   **Other:**  *Intervention*   - White (91%) - ≥University (47%) - Employed) (33%) - Income >$30,000 CAD (82%)   Control   - White (98%) - ≥University (60%) - Employed (37%) - Income >$30,000 CAD (84%) | **Intervention name**:  The Prostate Cancer-Patient Empowerment Program (PC-PEP)  **Supportive care type**: Home-based comprehensive and interactive online program, comprising physical activities, dietary education/ recommendations, stress management, & social support. Included emails with video, an initial PFMT nurse consultation, and optional text alerts.  **Delivery mode**:   - Online - SMS - Video   **Duration**: Daily offerings over 6-months | **Preference measure(s)**:   - Adherence to PF exercise regimen   **Preference Tools:**   - Pelvic Floor Muscle Training (PFMT) Compliance Surveys - IPSS - EPIC   **QoL Tools:**   - EPIC QoL section | - 78% (85% early, 71% late/control) preferred daily text alerts for PFMT reminders. - High compliance, with 70% adhering to regimen, averaging 20 min/day (76% of prescribed regimen). - Weekly adherence averaged 4.9–5.3 days/week over 26 weeks. | **Sample Size:** N=128    **Refusal Rate:** 16% (28/171) declined participation.  **Reasons for Refusal:** N/A  **Participation**: 91% (128/140) completed study, however only 1% (1/140) dropped out, others did not receive PCa treatment as intended.  **Dropout reasons:** N/A | **QoL**:   - No significant impacts on bowel or hormone-related   self-reported function   - improved sexual function in the surgery group   **Positive effects**: N/A.  **Negative effect**: None  **Cost-effectiveness**: N/A | **Setting:**   - health authority and university (n=1 each). - Delivered as standalone program, separate from standard clinical pathways   **Provider/Staff:**   - PFMT videos delivered by co-author & informed by advice from pelvic floor physiotherapists. Urology pelvic floor nurse delivered initial in-person training; followed by a 20-minute educational video by nurse PFMT specialists. - Staff adoption N/R | **Cost**: $200CAN per patient.  **Enablers**:   - program tailored to patient fitness & included diet, sleep hygiene, vitamin D, intimacy, sexuality, erectile dysfunction, and communication. - PFMT began with 20–30-min in-person session led by a urology nurse, followed by 20-min educational video. Participants received weekly 7–9-minvideos outlining PFMT routines (performed 3 times daily).   **Barriers**: Only 32% of 62 RP patients attended standard 3–4-week post-surgery PFMT nurse appointment (5 in PC-PEP, 15 in control). | **Organisational level:**: N/A.  **Individual level:** decreased trend in daily PFMT compliance over 6-mo, but still relatively high | Potential bias in self-reported urinary symptoms due to improved mental health in the intervention group. | The intervention significantly improved lower urinary tract symptoms, affirming its suitability for clinical integration alongside established mental health benefits in men with curative PC. |
| MacNevin et al. 2024  **Country**:  Canada  **Design**:  RCT  **Aim**:  Assess whether home-based patient empowerment program results in weight loss in men with PCa vs usual care | **Population**: men undergoing curative PCa treatment, eligible for robotic-assisted laparoscopic surgery or primary/salvage RT  **Sample**: N=128   - Intervention (n=66) - Control (n=62)   **Age:**   - Intervention median 65 yrs (range 50–78 yrs) - Control median 67 yrs (range 51–82 yrs)   **Stage**  *Intervention*   - Stage I (2%) - Stage II (75%) - Stage III (23%)   *Control*   - Stage I (3%) - Stage II (67%) - Stage III (30%)   **Treatment**  *Intervention*   - RP (44%) - RT (41%) - Salvage RT (15%)   *Control*   - RP (53%) - RT (44%) - Salvage RT (3.2%)   **Other**  Intervention   - White (91%) - Married/partnered (89%) - ≥University (47%) - Employed (33%)   Control   - White (98%) - Married/partnered (98%) - ≥University (60%) - Employed (37%) | **Intervention name**:  Prostate Cancer—Patient Empowerment Program (PC-PEP)  **Supportive care type:** Home-based program incorporating physical, mental, & social health activities including exercise & dietary education, & live online events.  **Delivery mode:**  Online  **Duration**: Daily offerings over 6 months. | **Preference measure(s)**:   - Adherence   **Preference Tools:**   - Weekly Online Adherence Surveys - Self-Reported Online Surveys - In-Person Assessments + Medical Chart Reviews   **QoL Tools:**   - In person assessments | - High adherence to exercise and diet recommendations. - Aerobic exercise: Avg. 4.3 days/week (86%) early-PCa group; 4.5 days/week (90%) late-PCa group. - Strength exercise: Avg. 2.1 days/week (105%) early; 2.4 days/week (120%) late. - Aerobic duration: Avg. 45 min/day (150%) early; 44 min/day (147%) late. - Strength duration: Avg. 28 min/day (93%) early; 30 min/day (100%) late. | **Sample Size:** N=128 *(secondary analysis of existing dataset)*    **Refusal Rate:** N/A  **Reasons for Refusal: N/A**  **Participation:** N/A | **QoL**: N/A  **Positive effects**: Significant weight loss (avg.2.7 kg) in early PC-PEP vs control.  **Negative effect**: None  **Cost-effectiveness**: N/A | **Setting:**   - health authority and university (n=1 each). - Intervention delivered as pilot study; not embedded into standard survivorship care pathways.     **Provider/Staff:**   - Certified Health Coaches provided personalized coaching sessions - Minimal impact on clinical staff; no direct involvement from oncology teams. | **Cost**: N/A  **Enablers**:   - Individualized intervention - daily video messages - web materials - Fitbits.   **Barriers**: N/A | **Organisational level**: Monthly live video conferences ongoing indefinitely (Phase 4 Pan-Canadian & International trial).  **Individual level:** Minimal group differences at 52 weeks; weight/BMI stabilized from 6–12 months in early PC-PEP group. | COVID-19 restrictions impacted the ability to conduct in-person assessments.  Secondary analysis of trial data, which may limit the findings’ scope & applicability. | The intervention had high adherence rates to dietary & exercise guidelines, &  led to significant weight loss in men undergoing curative PC  treatment compared to standard care, demonstrating efficacy across both early & later stages of treatment. |
| McGowan et al. 2013  **Country**:  Canada  **Design**: Prospective  three-armed RCT  **Aim**:  Determine effects of an  implementation intention intervention on physical activity  & QoL in PCa survivors | **Population**: PCa survivors  **Sample**: N=423 (n=141 in each arm)  **Age**: *M* = 68.4 yrs (range 47-89)  **Stage**:   - Local (58.6%) Locally advanced (20.6%) Metastatic (1.9%) Unsure (18.9%)   **Treatment**:   - Watchful waiting (27.7%) - Surgery (48.5%) - RT (44.9%) - Chemotherapy (2.8%) - HT (32.9%)   **Other:**   - White (94.1%) - Married (78.5%) - ≥University (38.3%) - Income >$80,000 (35.4%) - Full-time employed (25.3%) - Disease-free (49.2%) - Completed treatment (68.8%) | **Intervention name**: N/A  **Supportive care type**:   - self-administered group: physical activity guidelines + implementation intention intervention to complete at their own pace (including SMART goal setting) - phone-assisted group: self-administered group offerings + phone call to assist with goal setting & planning   **Control Group:**   - Standard physical activity recommendation: 2-page summary fact sheet of 2008 physical activity Guidelines for Americans   **Delivery mode**: Phone (phone-assisted group)  **Duration**: 3 months | **Preference measure(s)**:   - Engagement with material - Helpfulness of phone call (for phone group)   **Preference Tools:**   - Godin Leisure Time Exercise Questionnaire - Adherence Surveys (7-point Likert scale)   **QoL Tools:**   - shortform-36 health survey - Fatigue Scale | - 99% (320/322) read the PA guidelines and fact sheet by month 1. - In the telephone-assisted group: - 65% completed counselling. - 65% found it helpful for completing intervention. - 36% did not find it helpful for increasing activity. - 35% did not find it useful for planning. - Telephone group read materials in more detail than other groups. - In both goal-setting groups, 46% didn’t fully complete the activity; only 14% completed all of it. - Goal-setting activity took ~24 mins in self-administered and phone groups | N=423.  **Refusal**: 11% (~158/1500) actively declined participating, & 56% (836/1500) did not respond. In total, 28% of individuals contacted were enrolled in the study.  **Refusal reasons:**   - Disinterest - N/A   **Participation**: Overall, 87% (370/423) of randomized participants received the intervention: 98% (138/141) of PA only group, 99% (140/141) of intention group, & 65% (92/141) of telephone-intention group.  **Dropout reasons:** N/A | **QoL**: No significant changes reported  **Positive effects**:  PA increased in all groups, with greatest increase in intention group, followed by phone-intention, & standard PA groups.  **Negative effect**: N/A  **Cost-effectiveness**:  Self-administered, low-cost, time efficient planning & goal setting intervention may help PCa survivors initially increase their physical activity levels. | **Setting:**   - University - Implemented as a research trial; not integrated into standard survivorship care pathways.   **Provider/Staff:**   - Lead Researcher (PhD-level behavioural scientist) delivered phone-counselling interventions using semi-structured script. - No clinical staff involved. Research team responsible for intervention | **Cost**: stated/R  **Enablers**: Detailed examples & instructions in intervention materials.  **Barriers**: Being too busy with daily activities to engage in leisure activity | N/A | Modest completion rates of the goal setting & planning activity. | - A self-administered implementation intention intervention may have beneficial short-term effects on physical activity in PCa survivors. However, additional behavioural support strategies & more frequent intervention delivery are needed for longer-term behaviour change. - Phone group read materials in more detail than other groups. In both groups, few (14%) completed the goal setting & planning activity. Two-thirds in the telephone group found it helpful. - All groups including the PA recommendation control group resulted in a meaningful short-term increase in physical activity, but it did not maintain at 3 months or improve QoL. |
| Miah et al. 2022  **Country**:  UK  **Design**: Prospective cohort  **Aim**:  Evaluate feasibility, utilisation, & satisfaction of providing PCa patient information leaflets on electronic tablet | **Population**: Newly diagnosed PCa patients suitable for >1 curative treatment  **Sample**: N=112 (n=56 in each arm)  **Age**:   - Intervention median 66 yrs - Control median 70 yrs   **Treatment**: N/A  **Stage**: N/A  **Other**: N/A | **Intervention name**: N/A  **Supportive care type**:  Patient information leaflets (PILs)  **Delivery mode:**   - Control group: paper PILs. - Tablet group: paper PILs + electronic tablet with application containing electronic PILs & supplementary videos.   **Duration**: Tablet loaned for maximum 3 months. | **Preference measure(s)**:   - Satisfaction - Usefulness - Utilisation   **Preference Tools:**   - Telemedicine, Satisfaction and Usefulness Questionnaire - Patient‑related outcome measurements (PROMs)   **QoL Tools:** N/A | - No significant differences between groups. - High satisfaction with care in both groups (≥91% satisfied). - High satisfaction with the information received (≥80% satisfied). - (89%) of tablet group reported its utilization; of which (92%) considered tablet easy to use & 32% reported a preference for the paper format | N=112  **Refusal**: N/A  **Participation**:  Response rate 70% (78/112), & group response rates were 82% (46/56) for the tablet & 57% (32/56) for the control groups  **Dropout reasons:** N/A | **QoL**: N/A  **Positive effects**: N/A  **Negative effect**: N/A  **Cost-effectiveness**: N/A | **Setting:**   - healthcare institutions - Setting adoption N/R   **Provider/Staff:**   - Urology Clinical Teams created supplementary videos - Staff adoption N/R | **Cost**: N/A  **Enablers: N/A**  **Barriers**:   - Some participants preferred the paper format - Required tablet access | N/A | - Prospective cohort design rather than a RCT - Intervention group younger - Intervention group also had access to paper PILs. | The provision of electronic PILs in PCa treatment is a novel & potentially advantageous method of providing patient care, with high satisfaction & usability reported by patients. These should not replace paper PILS, which were preferred by a minority, and perceived as satisfactory overall. |
| Nanton et al. 2022  **Country**: UK  **Design**: Qualitative.  **Aim**: Describe context & rationale of Composite Holistic Needs Assessment Adaptive Tool – Prostate (CHAT-P). | **Population**: Men with PCa receiving treatment, monitoring, & follow up  **Sample**: N=16  **Age**: 50-85 yrs  **Treatment**:   - Unknown (19%) - Radiotherapy (RT) & hormone injections (HT) (12%) - RP (19%) - Active surveillance (50%)   **Stage**: N/A  **Other**:   - High self-reported computer literacy. - Time since start of treatment: - 0 -1 yr (12.5%) - 2-10 yrs (19%) - >10 yrs (12.5%) - Unspecified (56%) | **Intervention name:** Composite Holistic Needs Assessment Adaptive Tool-Prostate (CHAT-P)  **Supportive care type:**   - Adaptive needs assessment used before consultation. - assessing 11 domains: physical symptoms, emotional & psychological, religious/spiritual, information communication & relationships, independence & activity, rehabilitation & recreation, illness treatment & care, occupational, finance, legal. Included ‘red flag’ items (i.e., flagging clinician follow up if symptoms were urgent)   **Delivery mode**: Online via electronic devices.  **Duration**: N/A | **Preference measures**:   - Experience - Usability - Acceptability   **Preference tool:** FtF think-aloud assessment during use of CHAT-P. | - Overall positive regard for CHAT-P: comprehensive, user friendly & clear design - Improved access to online information - Helped identify needs & prepare for consultation. - Mixed feedback around disclosure, online vs FtF, & comorbidity questions - Time saving for clinician. - ‘Red flag’ feature important but only if confident clinician will address it. | N=16  **Refusal**: N/A.  **Participation**: 100% (n=16/16) of individuals enrolled in intervention completed it.  **Dropout reasons:** N/A | **QoL:** N/A  **Positive effects**:   - Identified & articulated patients' concerns, leading to more structured consultations. - Participants reported feeling more confident when communicating with clinicians. - system captured urgent symptoms (e.g., blood in urine, distress), prompting follow-up. - Links to trusted resources helped patients manage concerns.   **Negative effect**: Concerns about care team's response to identified issues.  **Cost-effectiveness**: N/A | **Setting:**   - Participants recruited from urology outpatient clinics (n=2) - Setting adoption N/R   **Provider/Staff:**   - Clinicians (urologists, oncologists, clinical nurse specialists, GPs) involved in developing care plan. - Clinicians valued concise, prioritised care plan summaries generated by CHAT-P. | **Cost**: N/A  **Enablers**:   - Coproduction with experts & PCa patients, iterative user testing.   **Barriers**:   - ensuring content was both holistic & specific for heterogenous group. - Secure design & data hosting (e.g., governance & agreements for software integration on clinician-facing websites, - Digital literacy & electronic device possession   **Adaptations**: Developed on flexible digital platform allowing adaptations & integration with electronic health records.  **Other:** Tested in two outpatient clinics & included in a feasibility study for primary care integration. Tool aligns with UK PCa guidelines.  . | N/A | - Most participants digitally literate. - Predominately white sample. - Unclear number of refusers - Small sample | - Tool allowed men to identify needs & communicate concerns to clinicians before consultation.   Unless tool is used during clinician consultations, it could increase unmet needs of patients. |
| Nilsson et al. 2020  **Country**:  Sweden  **Design**: Qualitative  **Aim**:  Identify usability, design & functionality issues in using e-Health  application  to support self-care  after PCa surgery | **Population**: Men who had undergone RP for PCa  **Sample**: N=15  **Age**: Range 56-72 yrs  **Treatment**: = RP  **Stage**: N/A  **Other**: N/A | **Intervention name**:  ePATH: an interactive e-HEALTH application  **Supportive care type**:  Tailored information & self-care activities, symptom monitoring, & communication with HCP.  **Delivery mode**:  An interactive e-Health application accessible through computer, smartphone, or tablet devices using secure authentication.  **Duration**: 4 weeks post-surgery. | **Preference measure(s)**:   - Usability   **Preference Tools:**   - Testing with think-aloud technique - Semi-structured interviews - Modified SUS   **QoL Tools:**   - ePATH access to tailored self-care activities | Digital was a good paperless alternative.   - **Usability:** User-friendly and helpful for self-management. - **Information Access:** Easy access to trusted rehabilitation info was highly valued. - **Functionality:** Clear structure and interface appreciated; some functions needed clarification. Mixed views on frequent symptom tracking. - **Engagement:** Motivation varied daily; psychoeducation on purpose of activities was key. - **Personalization:** Individualized content and app customization were important. - **Messaging:** Simplified contact with healthcare providers. | N=15  **Refusal**: 0% (0/15).  **Participation**: 100% (15/15). | **QoL**: N/A.   - **Positive effects**: Improved communication with HCP - support for self-management of self-care activities & symptoms.   **Negative effect**: Programming bugs lead to self-questioning digital literacy & reduced motivation.  **Cost-effectiveness**: N/A. | **Setting:**   - surgical department in a medium-sized hospital - Implemented as a usability study and not integrated into standard postoperative care pathways.   **Provider/Staff:**   - Contact Nurses introduced ePATH to patients, managed patient messages, and supported remote symptom monitoring - Nurses took on expanded role in supporting virtual rehabilitation, including providing remote education & responding to patient messages. | **Cost**: N/A.  **Enablers**: Clear & logical structure  reliable information  messaging function.  **Barriers**:   - Unclear functions - discomfort or lack of confidence in the technology - need for more personalization & customization. | **Organisational level**: Web platform available up to 12 months after treatment  **I Individual level**:  N/A | - Small sample size & homogeneous group. - Lack of log data on the frequency of actual use of different features. | Patients experienced ePATH as satisfactorily  user-friendly  & useful as a complementary self-management  support after PCa surgery,  especially when the information & tasks were tailored to their preferences & the system design features supported individual autonomy. |
| O’Connor et al. 2021  **Country**: UK  **Design**:  Single-arm pilot  **Aim**:  Examine efficacy, usability & acceptability of web-based self-management intervention to promote  sexual wellbeing in men with PCa | **Population**: Men receiving supportive care for PCa (active surveillance excluded).  **Sample**: N=109  **Age**: Range 50-69 yrs (74%)  **Treatment**:   - Surgery (6%) - RT (10%) - HT only (8%) - Combined therapy (22%)   **Stage**: N/A  **Other**:   - White (93%) - Received previous sexual care/ support (65%) - <6 months post- treatment (42%) - Partnered (91%) - Female partner (91%) | **Intervention name**:  N/A  **Supportive care type**:   - 5-step program designed to support sexual well-being: (1) sexual well-being & PCa, (2) changes & coping, (3) maintaining & improving sex life, (4) exploring sexual pleasure, & (5) facing the future. - Steps featured text-based information, infographics, videos highlighting patient experiences, & instructional videos delivered by HCP. - user toolkit containing series of quick guides included, as well as important behaviour change components & techniques, including use of social support, information about health consequences, instruction on how to perform a behaviour, demonstration or modelling of behaviour, & use of prompts, reminders, & cues.   **Delivery mode**:  Website  **Duration**: 3 months | **Preference measure(s)**:   - Usability - Acceptability   **Preference Tools:**   - Post intervention survey - System usability scale (modified) - Self-reported web-based survey   **QoL Tools**: N/A | *Usage:*   Median of 3 sessions completed (range: 1–11); median session duration: 22 min (range: 8–77); total use: 78 min (range: 18–284).   51% (45/89) completed ≥4 of 5 steps (classified as completers).   Session frequency and duration declined monthly but usage remained high in month 2 (85%) and month 3 (65%).  *Acceptability*   - Usability score: 19.68/24. - Highest agreement: trust in program (3.4/4) and usefulness of information (3.8/4). - Lowest agreement: visual appeal (2.9/4) and ease of use (3.0/4). | N=109  **Refusal**: 0% (0/125) of referred individuals declined to participate; 13% (16/125) were not eligible, & 87% (109/125) enrolled.  **Refusal reasons:** N/A  **Participation**: 82% (89/109) of enrolled completed the 3-month follow-up.  **Dropout reasons:** N/A | **QoL**: Improvements at 3 months in having a good understanding of managing PCa treatment on sex life & having a satisfying sex life.  **Positive effects**: N/A  **Negative effect**: N/A  **Cost-effectiveness**: N/A | **Setting:**   - Hospitals (n=2) - Successfully implemented across sites and online via a national charity platform.   **Provider/Staff:**   - HCP supported initial recruitment but had limited involvement during intervention | **Cost**: specified/R   - **Enablers**: Recommended to complete steps in order but offered flexibly - Visual progress indicators (tick marks) indicated completion.   **Barriers**: Usability issues & variation in engagement. | N/A | Active surveillance was an exclusion criterion | The tailored web-based intervention had good overall usability & acceptability, & improved sexual well-being  in men living with PC. The intervention may improve one’s self-perceived knowledge &  understanding of how to manage sexual issues & increase self-efficacy or the belief that a satisfactory sex life could be achieved  following treatment. |
| O’Connor et al. 2022  **Country**: UK  **Design**: Mixed methods  **Aim**: Assess acceptability & usability of a patient portal  developed to facilitate a remote monitoring & self-management  for men with PCa | **Population**: Men with PCa at low risk of recurrence, in follow-up care  **Sample**: 2,599  **Age**: M=72 yrs (range 44-96)  **Treatment**: N/A  **Stage**: N/A.  **Other**: N/A | **Intervention name**: N/A  **Supportive care type**:  Patient portal providing access to PSA results, messaging between patient & clinician, holistic needs assessment, & patient information.  **Delivery mode**:  Online patient portal  **Duration**: 12 months | **Preference measure(s)**:   - Portal utilisation - User experience - Satisfaction - Acceptability - Usability   **Preference Tools:**   - Structured phone interviews - Online Survey - Net Promoter Score (NPS)   **QoL Tools:** N/A | - 60% of eligible patients registered to use the portal. Of these, 37% logged in at least once over 6 months, & 52% over 12 months. - Most accessed portal when expecting a test result (41%), & only 13% accessed it once a month or more. - Patient information & Health MOT pages were viewed the least (14.5% & 14.3% of views respectively). - high satisfaction among users; > 70% found portal easy or very easy to use & 64% were classified as ‘promoters’. - Finding patient information, messaging clinical team, checking PSA results & completing Health MOT were each rated easy to use by>70% - Portal functions were rated helpful or very helpful by 78%–91% as helpful or very helpful in managing their condition. - most helpful functionality: access to PSA results (91% of respondents), followed by messaging the clinical team (87%). - small number of users preferred FtF clinical contact. | N=2,599  **Refusal**: 40% (1043/2599) of individuals did not register to use the portal. Across all recruitment pathways, a total of 21% (547/2599) did not participate in the study.   - **Refusal reasons**: lack of computer access (n = 10) - unwilling use - computers for healthcare (n = 8) - disliking computers   (n = 5)   - No internet access(n = 2).   **Participation**: Among all participants registered to use the portal, 34% (528/1556) participated in survey or interview.  **Dropout reasons: N/A** | **QoL**: N/A  **Positive effects**:   - Fewer hospital visits (72%) - Access to medical records (68%) - Information about condition (68%) - Messaging clinical team (73%) - Peace of mind via PSA access / messaging   **Negative effect**: Challenges with registration, login, and password management  **Cost-effectiveness**: N/A | **Setting:**   - NHS Trusts sites (n=5)   **Adoption at setting level**: Successfully adopted across sites. Participation ranged across sites from 42-73%.  **Staff:**   - Clinical Nurse Specialists oversaw patient management & supervision; Cancer Support Workers managed daily operations of program; General Practitioners & Hospital Phlebotomy Services conducted PSA tests; IT Specialists, Researchers, and Academics involved in program design and evaluation. - Clinical teams appeared to support the use of the portal. | **Cost**: not specifically reported.  **Enablers**:   - Easy to access results, messaging & patient info - Digital confidence   **Barriers**:   - Lower registration in older adults (drop after age mid 70s (<50%). Difficulty with technical tasks (e.g., registering, logging in & changing password) - Limited digital access/skills in some men linked to non-use of portal | **Organisational level**: Program has become normal practice at all initial five sites.  **Individual level**: 37% (575/1556) of those registered had logged in at least once over a 6-month period & 52% (810/1556) over a 12-month period | - The study did not relate patient outcomes to portal use. - Variation in uptake by cancer centre, possibly due to differing levels of encouragement & support from clinical teams. - Lack of detailed demographic data. | A large proportion of participants found the patient portal acceptable & easy to use. |
| Osei et al. 2013  **Country**:  USA  **Design**: Longitudinal RCT  **Aim**:  Explore effect  an online support system has on  QOL among men  with PCa | **Population**: Men diagnosed with PCa in previous five yrs  **Sample**: N=40 (*n*=20 in each arm)  **Age**: M= 67 yrs (range 53-87 yrs)  **Stage**: N/A  **Treatment**: N/A  **Other**  *Overall*   - White (85%) - Some college (30%) - College grad (30%) - Graduate school (30%)   Intervention   - White (95%) - Some college (35%) - College grad (35%)   Control   - White (75%) - Grad school (35%) | **Intervention name**: Us TOO website  **Supportive care type**:  Support group, with resource kits (pamphlets on treatment options, potential side effects, & approaches to dealing with side effects).  **Delivery mode**: Online  **Duration**: ≥3 times/week for 6 weeks | **Preference measures**:   - satisfaction - Quality of Life   **Preference tools:**   - Program Satisfaction Questionnaire – Us TOO Web Site   **QoL measures:**   - SF-12 version 2 - EPIC-26 - Satisfaction with Life Scale - Relationship Satisfaction Questionnaire | - 21% indicated all needs were met;37% reported that most of their needs were met. - satisfaction scale had *M* score of 3.0/4.0. - quality of services provided by online support group rated as good or excellent by 75% of intervention group - 89% of participants reported they received kind of service they wanted. | N=40  **Refusal**: 95% (949/1000 who were sent participation invites did not respond. Of the 51 who responded, 22% (11/51) chose not to enrol in study.   - **Refusal reasons:** - No internet access - language barriers - inappropriate timing   **Participation**: N/A | **QoL**: Improvements in urinary irritation & obstruction, sexual health, & hormonal health in the intervention.  **Positive effects**: N/A  **Negative effect**: N/A  **Cost-effectiveness**: N/A | **Setting:**   - Online intervention   Implemented within the context of a university-led research project rather than as a standard part of clinical care.  **Provider/Staff:**  No direct clinical staff involvement; support was peer-led via online platform. | **Cost**: N/A  **Enablers**: Online format providing anonymity & convenience.  **Barriers**: potential technological or emotional barriers. | **N/A** | - Lack of population diversity in ethnicity & educational background. - Short study duration (8 weeks). - Unknown resources accessed by the control group independently. - Low response rate (51/1000). - Demographic details including relevant history not provided | The online support group was effective in improving certain aspects of quality of life for participants in the short term, particularly in areas such as urinary irritation & obstruction health, sexual health, & hormonal health. However, these improvements returned to baseline by the end of the eight-week study period. |
| Pai et al. 2013  **Country**:  Canada  **Design**: Observational  **Aim**: Determine  experience & feedback regarding personal health record use while receiving care | **Population**: PCa patients  **Sample**: N=22  **Age**: Median 64 yrs (range 51-76 yrs)  **Treatment**:   - Not yet commenced treatment (18.2%) - active treatment (45.5%) - Completed treatment (9.1%) - Recurrence (27.3%)   **Stage**: N/A  **Other**:   - Married (71%) - White (95%) - Postsecondary school (75%) | **Intervention name:** Prostate Cancer Internet-based Delivery System of Electronic Records (PROVIDER)  **Supportive care type**: Personal health record (PHR) system  **Delivery mode**: Online  **Duration**: 6 months | **Preference measures**:   - Use - Satisfaction - Attendance   **Preference tools:**   - End-of-Study Survey - Usage Patterns   **QoL measures: N/A** | *Use:*   - Registered logins (6 months): 0–47; mean: 3.4/month; peak in first month. - Most accessed features: Medical records (94%); appointment viewer (82%); PSA monitoring (82%) - Most-used: Medical records - Record access (6 mo): median 19 times (range 1-39); records viewed median 45 (range 0-111); most reviewed: lab reports & doctors notes   *Satisfaction*   - Overall: 47% excellent, 41% very good, 12% good - Satisfied with medical access via PHR: 94% - Willing to continue use: 88%   *Session Attendance*   - CBSM group: attended 9–10 sessions (55%),7–8 (20%)2–6 (15%), 0-1 (11%) - HP group: 9–10 sessions (61%),7–8 (18%)2–6 (14%), 0-1 (7%) | N=22  **Refusal**: Not explicitly reported.  **Participation**: 77% (17/22) completed the study | **QoL**: N/A.  **Positive effects**: Enhanced communication with physicians (65%)  preserved privacy & confidentiality (77%), answered questions (65%)  access to new/ useful information (83%).  **Negative effect**: Dissatisfaction because of errors in PSA monitoring, missing reports poor layout/design, lack of printer-friendly formats, technical issues and privacy concerns.  **Cost-effectiveness**: N/A. | **Setting:**   - Cancer Agency and a University - Pilot successfully implemented using existing institutional resources and offsite hosting for the PHR.   **Staff:**   - Health information service clerks. - Minimal direct involvement from HCP in using platform for clinical care | **Cost**: N/A.  **Enablers**: Access to up-to-date medical records.  **Barriers**:  Hosting location of secure data/medical records.  dissatisfaction related to technical and accessibility aspects, privacy concerns (reported in negative effects)   - . | **N/A** | Predominantly well-educated white men | The intervention provided timely access to medical records, improved communication with healthcare providers, & empowered patients. Patients reported high satisfaction with the PHR system & found it useful for accessing new information & enhancing their understanding of their health condition. |
| Penedo et al. 2020  **Country**: USA  **Design**: RCT  **Aim**: Establish efficacy of tablet-delivered, group-based  psychosocial intervention for improving HRQOL & reducing symptom burden in men with advanced PCa | **Population**: Men with advanced PCa who had undergone ADT  **Sample**: N=192   - Intervention (n=95) - Control (n=97)   **Age**: *M*=68.84 yrs   - Intervention *M*=68.8 yrs - Control *M*=68.9 yrs   **Treatment**:   - ADT - RRP (Intervention 84%; Control 78%)   **Stage**: N/A  **Other**:  Intervention   - White (59%) - Married/partnered (71%) - Income ≥ $35,000 (69.5%) - Employed (33%) - Time post diagnosis to T1 *M*=4.36 yrs - Time post most recent ADT to T1 *M*=1.61 months   *Control*   - White (59%) - Married/partnered (63%) - Income ≥ $35,000 (61%) - Employed (44%) - Time post diagnosis to T1 *M* = 5.05 yrs - Time post most recent ADT to T1: *M* = 1.43 months | **Intervention name**:   - Cognitive-Behavioral Stress Management (CBSM), vs - Control   **Supportive care type**:   - Intervention: CBSM featuring cognitive restructuring, self-management skills, and relaxation skills training. - Control: Didactic material (e.g., session content) & expert videos (e.g., symptoms of ADT discussed by a urologist).   **Delivery mode**:  Web-based platform, with optional weekly contact with group facilitator & PCa survivors via  video conferencing.  **Duration**: 90 min online groups once/week for 10 weeks | **Preference measures**:   - Adherence - Satisfaction   **Preference tools:**   - Adherence measured by percentage of men attending sessions - Group satisfaction assessments - Weekly post-session online assessments   **QoL measures:**   - FACT-G - PROMIS Depression Item Bank computer adaptive test   (CAT)   - Fatigue Symptom   Inventory   - McGill Pain   Questionnaire   - MAX-PC - PSS - Impact of Events Scale-   Revised (IES-R)   - EPIC - Measure of Current Status (MOCS) - Social subscale of the Sickness Impact Profile (SIP) | *Attendance*  CBSM Group:   - 55% attended 9–10 sessions - 20% attended 7–8 sessions - 15% attended 2–6 sessions - 11% attended 0–1 sessions     HP Control Group:   - 61% attended 9–10 sessions - 18% attended 7–8 sessions - 14% attended 2–6 sessions - 7% attended 0–1 sessions | N=192  **Refusal**: 4% (196/4824) of individuals screened individuals declined participation. Most (91%; 4372/4824) ineligible.  **Refusal reasons**: N/A  **Participation**:   - of those enrolled, 89% (170/192) completed the intervention & 6-mo follow-up. Intervention group: 90% (84/95). Control: 90% (86/97). | **QoL**:   - No significant changes on quality of life. - Improvements in anxiety and distress   **Positive effects**: Relaxation increased over time for intervention group  **Negative effect**: Decreased sexual functioning scores in both groups (likely due to ADT).  **Cost-effectiveness**: N/A | **Setting:**   - hospital and medical centres - Successfully implemented across six clinical and academic settings.   **Provider/Staff:**  Group facilitators were master’s- or doctoral-level therapists who completed in-person training. | **Cost**: N/A  **Enablers**:  HIPAA-compliant web platform  study-provided tablet (also see adaptations).  **Barriers**: not specified.  **Adaptations**:   - Participant education on study purpose - Transportation and compensation for travel - On-site assessments - Flexible group scheduling - Device training and phone access for sessions   **Other**: | **Organisational Level/==**: N/A  **Individual Level**:   - 12-mo follow-up completion was 83% (77/95) in intervention, & 83% (75/97) in control. | - High baseline HRQOL may have limited the potential for improvement. - Attrition of participants with more severe symptoms at baseline.   . | Tablet-delivered CBSM & HP were well received by men with APC.  No significant differences in HRQOL & symptom burden between groups, but improvements in intervention targets were observed across conditions. |
| Rosenberg et al. 2016  **Country**: USA  **Design**: Mixed-method  **Aim**: Examine acceptability of wearable fitness tracker & attitudes towards integrating fitness  tracking into clinical care among men with PCa | **Population**: Men with non-metastatic PCa  **Sample**: N=26  **Age**: M=70.5 yrs  **Treatment**: ADT (54%)  **Stage**: N/A  **Other**:   - Non-Hispanic white (81%) - Married (73%) - Retired (69%) | **Intervention name**: N/A  **Supportive care type**: Daily physical activity monitoring  **Delivery mode**: Wearable fitness tracker (Fitbit Zip)  **Duration**: 2 weeks | **Preference measures**:   - Acceptability - Attitudes towards wearable - Ease of use - Usage   **Preference tools:**   - Fitbit Zip tracker - Semi-Structured In-Depth Interviews - Baseline Survey   **QoL measures:**  N/A | Acceptability   - Majority (93%) found Fitbit Zip easy to wear & comfortable. - 54%, reported using Fitbit during optional two-week phase. Of these, 43% had received ADT - Mixed value in device: some reporting it useful for monitoring & encouraging PA; others identifying they were active enough - Step count & distance walked were the two features used most by participants. Few used other features (e.g., calories, active min, challenges). - Many felt comfortable sharing Fitbit data with HCP but some were unsure how it was helpful | N=26  **Refusal**: 85% (174/205 people contacted for participation did not enrol/complete in-person visit).  **Refusal reasons**: N/A  **Participation**: 84% (26/31).  **Dropout reasons:**   - not wanting to wear Fitbit - unable to complete exit interview | **QoL**: N/A   - **Positive effects**: - Average use > over 6,000 steps/day when used. - value in sharing Fitbit data with health care team.   **Negative effect**: N/A  **Cost-effectiveness**: N/A | **Setting**   - health care system - Successfully implemented within research setting across multiple institutions.   **Provider/Staff:**  No direct clinical provider involvement in intervention delivery. | **Cost**: N/A  **Enablers:** Clear & simple instructions, assistance setting up device.  **Barriers**:   - Health limitations impacting physical activity. - Technical difficulties with setup and syncing. - Device did not track non-movement activities like weight training.   **Adaptations**: N/A  **Other**: Many men on ADT, & several with very low step counts (<4,000 steps/day) were able & willing to use fitness tracking during optional use phase.  se. | : N/A | - Small sample size - Limited generalizability to other groups or devices - Technological barriers impacted participants' overall experience | Findings support the use of easy to use & simple fitness trackers among men with PCa & there could be opportunities to integrate fitness tracker data into clinical care. Participants expressed value in sharing Fitbit data with their healthcare providers, though some faced technological barriers & health-related limitations. |
| Ruland et al. 2013  **Country**:  Norway  **Design**:  Single arm analysis RCT  **Aim**:  Describe patients use of a multi-component  eHealth application to support cancer management | **Population**: PCa and BCa patients  *Note: PCa patients reported below*  **Sample**: n=47  **Age**: *M*=65 yrs  **Treatment**:   - Chemotherapy (6%)   **Stage**: Metastatic or recurrence (24%)  **Other**:   - Married/cohabitating (92%) - University/college (43%) - 12-month post diagnosis (53%) - >1 yr post diagnosis (43%) | **Intervention name**:  WebChoice: a multi-component  eHealth application.  **Supportive care type**:  interactive eHealth application that includes  information that is personalized & interactively  tailored to patients’ needs, symptom monitoring, access to nurse specialist, communicate with other patients via a forum, & a diary.  **Delivery mode**:  Online application  **Duration**: 1 yr | **Preference measures**:   - Frequency & patterns of use - Perceived usefulness - Reasons for use of each web section   **Preference tools:**   - User Logs for WebChoice application - Self-constructed questionnaire - Forum and Email Messages   **QoL measures:**  N/A | *Use*   - Median number of logins per participant: 10 (range: 2–593) - Most frequently used sections (median uses): assessments: 5.0; self-management support: 5.0; personal message to nurse: 4.0; discussion forum: 4.0;information section: 3.0; Diary: 2.0   *Reasons for use*   - Self management - General information (about problem, find assurance - )Self-management intervention (n=30: getting help with a problem (73%), prepare communication with HCP (40%). - General information (n=27): find information about a problem (56%), gain assurance relating to disease or treatment (59%), prepare communication with HCP (52%) - Discussion forum (n=25): compare experiences with other patients (64%), information about a problem (56%), assurance relating to disease or treatment (52%) - Message to nurse (n=21): information about a problem (76%), support or assurance (38%)   *Feedback*   - nurse postings in discussion forum reassuring, - 57% wished for increased nurse forum contributions. | N=47 PC  **Refusal**: N/A  **Participation**: N/A for PCa group; 64% overall classified as actively using the site | N/A. | **Setting:**   - Participants recruited via National Cancer Registry, national newspapers, and Cancer Society. - Successfully integrated into large multi-institutional clinical trial.   **Provider/Staff:**  CNS actively monitored and engaged with patients via the platform. | N/A. | N/A | - Self-selected sample of cancer patients - Did not account for other factors influencing patterns of use | - Results suggest that the various sections in WebChoice can meet many patients’ need for support & information - PC patients valued private, personalized communication with clinical nurse specialists. - demonstrated a significant need for decision-making support & preparation for communication with healthcare personnel, likely due to the complexity & variety of treatment options available for PCa. |
| Scherr et al. 2022  **Country**:  USA  **Design**:  RCT  **Aim**:  Develop shared decision-making training video, measure usability & satisfaction, & determine its effect on  preparing patients to participate in clinical appointments | **Population**: Patients with early-stage PC  **Sample:** N=208  **Age**: *M*=61.6 yrs (range 43–84 yrs)   - Intervention *M*=61.2 yrs - Control *M*=61.9 yrs   **Treatment**: N/A (pre-treatment)  **Stage**: N/A  **Other**  *DA only*   - White (86.7%) - College (62.3%) - Married/partnered (79.6%)   *DA+ Video*   - White (89.1%) - College (66.4%) - Married/partnered (85.5% | **Intervention name**: N/A  **Supportive care type**:  Shared decision making (SDM) training video combined with a DA  **Delivery mode**: Video & booklet  **Duration**: Single 26min training video before clinical appointment | **Preference measures:**   - Use & evaluation of study materials - Recommendation o   **Preference tools:**   - Surveys - Control Preferences Scale - Intentions to Seek Other Opinions - Chronic Disease Self-Efficacy Scale (adapted)   **QoL measures:**   - SDM-Related Behavioural Intentions | - 76% of participants spent ≥30 min reading DA - 92% would recommend DA, no significant difference between groups. - DA+SDM group: 91% watched the entire video, & 93% would recommend it. - Participants felt amount of information provided appropriate, no significant difference between groups. - 95% agreed the information balanced. Information was rated as trustworthy across both groups, with higher ratings in DA+SDM | N=208  **Refusal**: N/A  **Participation**: 97% (107/110) of intervention group diagnosed with localized PCa completed pre-appointment survey. 95% (93/98) of control group completed survey. | **QoL**: N/A  **Positive effects**: DA+SDM group:  increased desire to participate in decision, less decision urgency, & improved self-efficacy for communicating with physicians.  **Negative effect**: None.  **Cost-effectiveness**: N/A | **Setting:**   - university-affiliated hospital (n=2 sites) - Successfully integrated into clinic workflow during biopsy appointment phase   **Provider/Staff:**   - No direct involvement of clinical staff in delivering the intervention | **Cost**: N/A.  **Enablers**:   - Plain language & simple graphics in DA - accessible to men with eighth-grade literacy levels   Two versions of the final video: 1 primarily  featuring a black patient actor & 1 a white patient actor. Patients received version featuring an actor that matched self-identified race.  **Barriers**: N/A  **Adaptations**:  **Other**: | N/A | - Unclear whether increases in self-efficacy & intentions will translate into actual empowerment during clinical appointments - Limited racial representation in the training video | The decision-making training video was well received & highly used by patients.  The video combined with a DA was more effective at improving several  measures of shared decision-making outcomes (e.g., increased desire to participate, self-efficacy) compared to  receipt of the DA alone.  Videos may be an ideal low-resource format to deliver shared decision-making training to patients facing  preference-sensitive decisions. |
| Schrijvers et al. 2013  **Country**: Belgium  **Design**: Observational  **Aim**:  Evaluate use of web-based DA in their decision-making process for PCa treatment | **Population**: Patients newly diagnosed with LPCa.  **Sample**: N=74  **Age**: M= 69 yrs (range 65-75)  **Treatment**: N/A.  **Stage**: LPCa  **Other**: = School leaver (40.5%) | **Intervention name**: N/A  **Supportive care type**:  DA (consisting of Patient Testimonials, Scientific Information, Comparative Tables (treatment options, benefits, & harms), Values Clarification Tool, & Medical Glossary) & accompanying website (consisting of 6 informative sections: Introduction, The Prostate, Treatments of PC, Choosing Treatment, Relapsed & advanced PC, & a glossary/ search function/support links).  **Delivery mode**:  Online platform  **Duration**: not specified (unspecified time between baseline & next physician appointment) | **Preference measures**:   - Frequency of page visits - Time spent on each page - Use of technology features   **Preference tools:**   - Web-log data (visited pages) - Patient Statements   **QoL measures:** N/A | - Men spent average 30 min on DA. - most frequently used: comparative tables on treatment options (2.89 times); side effects (2.25 times); values clarification tool (1.51 times). - The values clarification tool and information on PCa pages visited by all but one participant at least once. - least popular section was medical dictionary, search function, links, & contact information. - Participants >70 yrs spent more time on pages related to active surveillance and decision-making support. - Younger participants (65–70yrs) spent more time on surgery and emerging treatment options. | N=74  **Refusal**: N/A  **Participation**: 87% (87/100) who agreed to participate logged on the DA at home.  **Dropout reasons:**   - improper registration - not accessing website - inadequate time on website. | **QoL**: N/A.  **Positive effects**: N/A.  **Negative effect**: N/A.  **Cost-effectiveness**: N/A. | **Setting:**   - Urology department of university hospital - Implemented successfully within hospital’s standard workflow for newly diagnosed PCa patients.   **Staff:**   - Urologists provided initial diagnosis & minimal treatment information; research staff introduced DA, provided instructions over its use, & answered technical questions during initial exposure.   **Provider/staff**   - intervention fit into clinical workflows, requiring minimal additional time from HCP | **Cost**: N/A.  **Enablers**:   - On-site technical support from a researcher. - Flexible navigation: participants could browse content linearly or nonlinearly using table of contents or page arrows.   **Barriers**: Non-use or limited use by some participants (reasons unidentified)  **Adaptations**: N/A.  **Other**: N/A | :  N/A | - Small sample size - Lack of diversity in the patient population - Exclusion of outliers in time spent on the DA, which could indicate thorough reading | The majority of 65+ persons with good internet skills use a web-based DA as well as its incorporated technology  features. |
| Skolarus et al. 2019  **Country**: USA  **Design**: RCT  **Aim**:  Compare a personally tailored, automated phone symptom management intervention to improve self-management among survivors of PCa vs usual care | **Population**: Veteran PCa survivors 1-10 yrs post-treatment  **Sample**: N=556   - intervention (n=278) - Control (n=278)   **Age**: M=67 yrs (range: 49 to 83 yrs)  **Treatment:**   - RT (57%) - Surgery (46%) - ADT (25%) - Other treatments (1%)   **Stage**: N/A  **Other**   - Married (54%) - White (70%) - Retired (62%) - Income <$50,000 (75%) | Intervention name:   - Building Your New Normal, vs - Control   **Supportive care type**:   - Intervention: a personally tailored, automated telephone symptom management intervention with tailored newsletters - Control: UC with non-tailored newsletter describing self-management approaches to PCa symptoms. - **Delivery mode**: Automated phone system - mailed newsletters   **Duration**: 12 months. | **Preference measures**:   - Experience - Satisfaction   **Preference tools:**   - Automated telephone system - Baseline telephone survey - Follow-up surveys   **QoL measures:**   - EPIC-26 - Veterans RAND 12-item health survey | - high intervention satisfaction: 63% of intervention & 67% of control reported being very satisfied - 76% of intervention participants received all 4 newsletters. Most chose one (n=93) or two (n=92) focus areas, followed by three (n=62) & four (n=16). - most common focus areas selected initially: sexual health (chosen by ~75%)urinary, bowel, & general; - nearly 80% of intervention participants never chose bowel health. | N=556  **Refusal**:  60% (932/1562) of approached individuals actively declined participation.  36% (556/1562) consented.  **Refusal reasons**: N/A.  **Participation**: 91% (504/556) completed 5-mo survey, with  88% (244/278) of intervention, & 94% (260/278) of control completed 5-mo survey. | **QoL**:   - Non-significant improvements in QoL in all domain areas at 5 months, higher coping appraisal scores in intervention at 5 months. - at 12 months, higher coping appraisal scores in intervention   **Positive effects**: N/A.  **Negative effect**:   - At 12 months, subjective physical health was lower in intervention than control - no differences in subjective emotional health.   **Cost-effectiveness**: Low costs for automated phone calls ($0.65 for control & $1.40 for intervention). | **Setting:**  Veterans Affairs sites (n=4)  Successfully implemented across sites  **Provider/Staff:**   - Automated intervention - Clinical experts (urologists, nurses, mental health professionals) contributed to development of tailored content but not involved in direct patient support. | - **Cost**: Total call minutes: 25,777 outbound and 8,888 inbound - Total call costs: $531 - Per participant: $0.65 (control) and $1.40 (intervention)   **Enablers**: Tailored content, & automated phone system.  Participants could choose a symptom area and switch monthly for tailored  self-management print materials (urinary, sexual, bowel,  general)  **Barriers**: N/A  **Adaptations**:  **Other**: N/A | **Organisational Level/Setting**: N/A  **Individual Level/FUp**:  5 and 12 month follow up assessments after enrolment for both groups (assessed QoL) | Veterans without telephones or the ability to use automated telephone systems could not enrol. | This intervention was well received among veterans who were long-term survivors of PC. Although overall outcome differences were not observed across groups, the intervention tailored to symptom area of choice may hold promise to improve associated burden |
| Song et al. 2015  **Country**: USA (North Carolina)  **Design**: Mixed method  **Aim**:  Evaluate feasibility & acceptability  of a newly developed web-based, couple-oriented  intervention | **Population**: Patients with LPCa  & their partners.  **Sample**:   - N=26 dyads - N=26 PCa patient - N=26 partners)   *Note: PCa patients reported below*  **Age**: *M*=63 yrs  **Treatment**:   - RT (59%) - Surgery (41%)   **Stage**: N/A  **Other**:   - White (100%) - College (59%) - Income >$75,001 (34.6%) - Distance from home to hospital M=82 miles - time since diagnosis *M*=19 months | **Intervention name:** Prostate Cancer Education & Resources for Couples (PERC).  **Supportive care type**:   - Couple-oriented intervention to manage PCa treatment-related symptoms & improve QoL through 7 educational modules & interactive features. - Includes mandatory modules on teamwork & survivorship issues, & optional modules on managing specific symptoms, audio-enhanced presentations, video clips, & assignments to encourage collaborative symptom management, & also provided PCa Resource Centre with web links to different organizations & online resources.   **Delivery mode**:  Website  **Duration**: 8 weeks (1 module each week for 7 weeks, each module requiring ~ 10–20 min) | **Preference measures**:   - Use - Usability - Satisfaction   **Preference tools:**   - Web activity data tracking - Pre + post-pilot questionnaire + assessments/interviews   **QoL measures:**  FACT-G | *Use*  Average Logins:   - Per patient: 2.7 times - Per couple: 3.64 - Per partner: 2.68   Time Spent:   - Per patient: 42 minutes - Per partner: 44 minutes - Per couple: 57 minutes   Most Visited Sections (Patients):   - Sexual dysfunction: 77% - Fatigue: 77% - Urinary dysfunction: 76% - Assignments and exercises: 94%   Joint vs. Individual Use:   - 8 couples always logged in together - 5 always logged in separately - 9 alternated between joint and individual use   *Satisfaction*   - Program Information: 3.1/5 - Quality of Information: 3.1/5 - Time Allocated Weekly: 2.7/5   *Usability:*   - Ease, attractiveness, interestingness, responsiveness: 4.1–4.6/5 - Clarity, simplicity, quality, relevance of content: 4.1–4.4/5 - Overall usability: 4.2–4.4/5 - Weekly Exercises & Assignments: 3.7/5 - Audio-enhanced slides and videos: Used by 83% to support low literacy | N=26 dyads  **Refusal**: 49% (25/51)  **Reasons**: Disinterested or did not respond.  **Participation**:   - 96% (25/26 dyads) completed the intervention. - 85% completed the postintervention survey.   **Dropout reasons**:   - Family death (n = 1) - Family illness (n = 1) - Lost to follow-up (n= 2). | **QoL**:  Urinary irritability and bowel dysfunction  Physical and social well-being  General symptom reduction  **Positive effects**:  Better symptom management  Improved patient-partner communication  **Negative effect**:   - Decreases in couples’ cancer communication & relationship satisfaction - Worsened sexual dysfunction over time.   **Cost-effectiveness**: N/A. | **Setting:**   - Participants recruited via university oncology outpatient clinics. - Successfully implemented within the outpatient oncology clinic context.   **Provider/Staff:**   - Development team included nurses, physicians, a psychologist, a media specialist, web designers, and programmers; urologic oncologists and nurse practitioners reviewed intervention content; Research staff managed recruitment, consent, & provided technical support. - HCP participated in content development but had no active role during intervention delivery. | **Cost**: N/A.  **Enablers**: Weekly email reminders to encourage completion of program elements.  **Barriers**: Some participants experienced difficulty adjusting to new ways of relating to each other. | N/A | - Participants had relatively mild symptoms & good quality of life preintervention, which may limit generalizability. - Lack of control group & long-term monitoring to identify whether negative relationship & sexual changes were result of intervention, &/or temporary | - PERC was a feasible & acceptable method of reducing the side effects of PCa treatment-related symptoms & improving quality of life for patients & their partners. - intervention provided valuable, high-quality, & relevant content, & participants reported improved knowledge about symptom management & communication as a couple. - negative effects were observed in couple communication & sexual dysfunction. - Introduction of new relationship communication patterns among long-term couples (without a control group) may have contributed to negative relationship & sexual outcomes & should be considered in future dyad-related research. |
| Song et al. 2021  **Country**: USA  **Design**:  Two-group randomized pilot trial  **Aim**:  Examine feasibility & outcomes of enhanced survivorship care plan that integrates web-based patient education resources for couples into  a standardized survivorship care plan | **Population**: Patients with LPCa who had recently completed initial curative intent treatment & partners    **Sample**: N=62 *couples*   - PCa patients (n= 62) - Partners (n=62) - Intervention (ESCP n= 31 couples/62 people) - Control (SCP n= 31 couples/62 people) - (n=32) ESCP, n=31 SCP)   *Note: PCa patients reported below*  **Age**:   - Intervention M=64 yrs - Control M=65 yrs   **Treatment**:  *Intervention*   - RT: 55% - Surgery: 45%   Control   - RT: 52% - Surgery: 48%   **Stage**: N/A.  **Other**:  Intervention   - White (71%) - <College (52%) - Income >$60,000 (65%)   Control   - White (77%) - <College (42%) - Income >$60,000 (61%) | **Intervention name**: Patient Education Resources for Couples (PERC)  **Supportive care type**:  mHealth program with patient education resources for couples Regarding symptom management & healthy behaviours. Also provided social support via online forum & meetings with a health educator.  **Delivery mode**:  Online mHealth  **Duration**: 4-6 months | **Preference measure(s)**:   - Feasibility (recruitment & retention) - Satisfaction - Perceived ease of use   **Preference Tools:**   - Usability Scale - Website tracking - Self-reported data - Post-exit interviews - EPIC-26   **QoL Tools:**   - Cancer Self-Efficacy Scale - Functional Assessment of Chronic Illness Therapy General Scale, FACTG | **Intervention**   - patients reported greater program satisfaction & easier navigation than control - 70% of intervention viewed pages aligned with symptoms. - About one-third of intervention did not access the PERC platform at all. - Participants appreciated convenience of accessing credible information online & ability to choose what information to view. - most frequently visited PERC webpages by dyads were sexual dysfunction, working as a team, working with healthcare team, hormonal, urinary, & sleep disturbance. | N=62 dyads.  **Refusal**: 41% (87/211) of approached individuals refused participation or were lost to follow-up. In total, 29% (62/211) consented to participate.  **Refusal reasons**: N/A  **Participation**: Overall, 85% (53/62) of dyads who consented received the intervention completed the study. Participation for each group was 90% (28/31) of the ESCP group, & 81% (25/31) of the SCP group.  **Dropout reasons**:   - personal issues - unable to contact - ineligibility. | **QoL**: Non-significant trends towards better QoL & urinary symptom scores for intervention  **Positive effects**: numbers of medical visits for intervention patients were 0.2 fewer than  those control patients within 10 months  **Negative effect**: N/A.  **Cost-effectiveness**: N/A. | **Setting:**   - Participants recruited from genitourinary and radiation oncology clinics at comprehensive cancer centres (n=4). - Successfully implemented in cancer centres during routine SCP delivery.   **Provider/Staff**:   - Nurses created & reviewed SCPs with patients & partners, embedding appropriate web links (PERC or NCI); research staff recruited participants, collected data, and managed participant support; Health educators & clinical experts contributed to development of intervention - Nurses integrated SCPs into routine clinical care and reviewed them with patients | **Cost**: N/A.  **Enablers**: N/A.  **Barriers**: Being too busy or not needing information (i.e. few symptoms) | : N/A | Homogeneity of sample, who were mainly white & heterosexual. | The ESCP intervention was feasible & showed promising results in improving urinary symptoms, coping resources, & QOL, & reducing medical visits. |
| Steginga et al. 2008  **Country**: Australia  **Design**: Pre-post  **Aim**: Assess feasibility of phone-delivered supportive care intervention for men newly diagnosed with PCa | **Population**: Men with newly diagnosed & LPCa  **Sample**: N=20  **Age**: *M* age 63 yrs (range 50–76 yrs).  **Treatment**:   - RP (35%) - Brachytherapy (35%) - External beam radiation (10%) - External beam radiation + brachytherapy (5%) - Watchful waiting   **Stage**: N/A  **Other**:   - Married or in a relationship (85%) - Postsecondary education (65%) - Retired (50%) - income <$ $40,000 (50%) - M=14 days post- diagnosis (range 3–31 days) | **Intervention name:** ProsCan  **Supportive care type**:  Decision-making support intervention, with support through PCa-related psychological & physical challenges via 4 pre-planned, guided phone sessions that followed structured counselling protocols.  to cancer, problem solving & coping with  treatment side effects).    **Delivery mode**: phone  **Duration**: 4 phone sessions/12 weeks | **Preference measure(s)**:   - Session use, frequency, duration & content - Intervention satisfaction   **Preference Tools:**   - 1-10 satisfaction scale (“not at all satisfied” to “extremely satisfied”). - Verbatim responses on suggested improvements. - DCS (revised)   **QoL Tools:**   - Decision-Related Distress scale (DRD) - Brief Distress Thermometer (BDT) | - High satisfaction with intervention, for both phone counselling sessions (*M* 8.5/10) & support materials (8.3/10). - 25% indicated counselling calls most helpful, others rated treatment decision making, information on treatment side effects, relaxation training, & coaching with pelvic floor exercises as most helpful. - The telebased approach enhanced access to support when physicians were unavailable or information was unclear. - Intervention contributed to reduced cancer-related distress, lower decisional conflict, improved clarity of values, and fewer intrusive thoughts.   Usage:   - Average number of pre-treatment calls: 3.4 (range: 2–5). - 55% required ≥4 due to decisional uncertainty or distress. - Undecided men received more calls (3.8) than those who decided (2.7). - Call durations: Pre-treatment: 40.2 min (range: 25–62); Post-treatment: 30.9 min (range: 12–52) - Common pre-treatment topics: psychoeducation, decision support, side effects, sexuality, thought-challenging, relaxation. - Post-treatment topics: side effects, sexuality, goal setting, activity management, adjustment. | N=20  **Refusal**: 23% (6/26) refused participation.  **Refusal reasons**:   - Not wanting reminder of cancer - not wanting help from others - being too busy.   **Participation**: 100% | **QoL**: general decrease in cancer-specific  distress symptoms over time  **Positive effects**:   - Decreased decisional conflict, intrusion symptoms, improved clarity of personal values. - For men who denied undue distress, engagement was facilitated by normalising & reinforcing adaptive - coping & decision-making strategies.   **Negative effect**: N/A.  **Cost-effectiveness**: N/A. | **Setting:**   - private urology practices (n-3) - Successfully implemented in practices & remotely via phone   **Provider/Staff:**   - PCa CNS counsellors, trained in decision support, psycho-oncology, continence, and sexual functioning; Clinical psychologist with oncology expertise oversaw nurses   **Provider/staff:**  CNS adopted intervention enthusiastically and delivered it effectively according to structured protocol. | **Cost**:   - Counselling: $18.60/session; average 5.5 sessions = ~$102.30 - Materials: $9.30   Total cost per participant: USD~$111.60  **Enablers**:   - phone anonymity & accessibility. - self-help worksheets supported telephone-delivered counselling. - Additional pre-treatment calls where men experienced persistent high decisional uncertainty or distress   **Barriers**: One participant reported books currently available for men with PCa are seen as out of date.  **Adaptations**:  . | N/A | Lack of control group | - phone-delivered supportive care & decision support intervention was feasible & highly acceptable - intervention effectively reduced decisional conflict & cancer-specific distress, with high levels of satisfaction reported by participants. |
| Sundberg et al. 2015  **Country**: Sweden  **Design**: Qualitative  **Aim**: Test feasibility & acceptability of an Information & Communication Technology platform for smartphone use for symptom reporting during  radiotherapy for PCa | **Population**: PCa patients  receiving RT  **Sample**: N=9  **Age**: *M*=69 yrs  **Treatment**:   - RT (100%) - Brachytherapy (55.6%) - Neoadjuvant HT (67%)   **Stage**: N/A | **Intervention name**: N/A  **Supportive care type**:  Symptom reporting & management to enhance communication between patient and provider.  **Delivery mode**:  Information & Communication technologies (ICT) platform  **Duration**: 2 weeks | **Preference measure(s)**:   - Satisfaction - Use - Feasibility - Acceptability   **Preference Tool:**   - Logged website data - Qualitative content analysis of interviews - Symptom Questionnaire   QoL Tools:   - Self-care Advice - Patient Reported Outcomes (PROs) | Satisfaction   - Patients found symptom questionnaire & self-care advice relevant & user-friendly. - Self-care advice accessed by majority (85%) of participants. - Most frequently reported symptoms were flushing (n=52), urinary urgency (n=50), & fatigue (n=44).   Use & Acceptability   - All participants indicated application was easy to use. - Majority found it an interesting & worthwhile experience - Daily symptom monitoring (via alerts) reassured participants & fostered sense of security. - Suggested message function to explain symptoms would prepare HCP of patient's needs. | n=9  **Refusal**: 90% (9/10 approached enrolled in study).  **Refusal reasons**:  Unwilling to participate.  **Participation**: 100% | **QoL**: N/A.  **Positive effects**: Facilitated symptom assessment & communication  **Negative effect**: N/A.  **Cost-effectiveness**: N/A | **Setting:**   - university hospital (n=2 sites) - Successfully implemented in clinical centres   **Provider/Staff:**   - 8 nurses (4 at each site) - Overall positive response from nurses. Nurses adopted system & actively engaged in it during study period | **Cost**: N/A.  **Enablers**: Alert system prompted nurse outreach during severe symptoms, enhancing patient safety and care  **Barriers**:   - Some found daily reporting burdensome - Pain reporting was confusing when symptoms were occasional - Difficulty in rating sleep problems, as the 24-hour reference period conflicted with nighttime sleep patterns | N/A | - Small sample size - Homogeneous sample regarding age & clinical characteristics - Short reporting period (mean of 10 days) | - patients found the symptom questionnaire & self-care advice relevant & application user friendly. - platform enabled increased patient involvement & facilitated symptom assessment & communication between patient & HCP |
| Tagai 2021  **Country**: USA  **Design**: RCT | **Population:**  Men with LPCa 1 yr post-treatment  **Sample Size**: N=431   - Intervention (n=217) - Control (n=214)   **Age**: *M*=63.5 yrs  **Stage**: LPCa,  **Other:**   - White (70.6%) - Married/partnered (79.5%) - ≤Highschool (24.5%) - Health literate (*M* score 13.0/15) - Income ≥ $75,001 (53.9%) | **Intervention name**:  Prostate Cancer Online Guide & Resource for Electronic Survivorship (PROGRESS)  **Supportive Care Type:** Educational, cognitive, behavioural, & emotional support via web-based resources, guided by Cognitive-Social Health Information Processing (C-SHIP) model.  **Mode of delivery:**  Website  **Duration:** N/A | **Preferences measure (s)**   - PROGRESS usage (assessed by Google Analytics)   **Preference Tools:**   - Google Analytics   **QoL measures (s)**   - Cancer Coping Questionnaire (14 items; 5 subscales) - EPIC - Self-efficacy for re-entry & symptom control - Practical Concerns Scale - Medical & Marital Interaction Scales | - Only 38.7% of those assigned to PROGRESS used the website. - Users were slightly older & had higher health literacy. - Reasons for non-use not assessed in detail. - Some users preferred print-based materials alongside digital tools. | **Sample Size**: N=431  **Refusal Rate**:   - 24% (218/927) of individuals assessed for eligibility declined to participate - 56% (278/496) were not eligible.   **Refusal reasons**: N/A  **Participation Rate**:   - Overall, 76% (326/431) randomised to condition completed 1-mo survey & 49% (210/431) completed 6-mo survey. - For intervention, 73% (159/217) completed 1-mo survey & 48% (103/217) completed 6-mo survey - For control, 78% (167/214) randomised to condition completed 1-mo survey & 50% (107/214) completed 6-mo survey   **Dropout reasons**: N/A | **Quality of Life Measures:**   - Improved in urinary incontinence, irritation, & sexual function (both groups**)**   **Positive Effects:**   - Intent-to-treat: Improved diversion coping, smaller decline in interpersonal coping - As treated: Fewer practical concerns (users vs. non-users)   **Negative Effects:**   - Worse marital communication (intent-to-treat) - Worse positive coping among users (as treated**)**   **Cost-effectiveness: N/A** | **Setting:**   - cancer centres (n=4) - Setting adoption N/R   **Provider/staff:**   - Research staff introduced study and supported consent and data collection. - Self-guided intervention; no staff training | **Cost: N/A**  **Enablers:**  Health literacy & older age  **Barriers:**   - Lack of interest in web tools - potential need for better content organization - lack of engagement features - limited tracking metrics | **Organisational/Staff Level**:  N/R  **Individual level:**  Usage not tracked over time beyond a single login; no long-term use assessed | - Conducted at academic cancer centres - Only participants with internet access included - Low intervention engagement - Limited website usage metrics - No follow-up with non-users - Usability testing was limited to lab settings | PROGRESS may support coping with practical concerns among PCa survivors, but poor engagement & usability limit its potential. Future interventions must prioritize skill-building content & user engagement strategies to better align with participant preferences. |
| Taylor 2010  **Country**: USA  **Design**: Two-arm RCT  **Aim**: Evaluate use, satisfaction, & impact of a CD-ROM-based DA on treatment decision-making among newly diagnosed early-stage PCa patients | **Population:**  Men newly diagnosed with early-stage PCa who had not yet made a treatment decision.  **Sample Size**: N=132   - Intervention (n=95) - Control (n=25)   **Age**: *M* = 64.6 yrs (SD = 9.4)  **Stage**: Early-stage (T1–T2N0M0)  **Treatment**: N/A (newly diagnosed)  **Other:**   - White (74%) - Married (80%) - Graduate degree (56%) - Employed (62%) - Insured (99%) - Computer access (98%) | **Intervention name**: N/A  **Supportive Care Type:**  Educational DA to support informed decision-making (info on treatments, side effects, pros/cons, etc.)  **Mode of delivery:** Interactive CD-ROM  **Duration:**  Median 72 min spent using informational content | **Preferences measure(s):**   - Usage - Acceptance   **Preference Tools:**   - Degner Control Preference Scale - Wallston Desire for Control Scale (DCON) - Satisfaction with Preparation for Decision Making (SPDM) - DCS (short form) - Knowledge test (23-item custom tool) - UCLA Prostate Symptom Index - SF-12 Health Survey   **QoL Tools:**   - Mental Component Summary (MCS) - Physical Component Summary (PCS) | *Usage:*   - 79% used the CD-ROM; median time = 72 min - Tools under utilized relative to static content; participants preferred narrative/info over interactive comparisons   *Perception:*   - 90% found CD balanced & fair - 91% found info clear - 63% said info was “about right” in quantity - 76% said the CD increased their sense of control - 77% used the CD as much/more than other sources - Users preferred information over decision tools; less time was spent with interactive comparison tools   **Satisfaction**:  Satisfaction with CD significantly greater among non-white participants, those with higher MCS & less knowledge about PCa or desire for control over treatment; non-significantly greater among retired/unemployed and those who received immediate treatment | **Sample Size**: N=132 enrolled  **Refusal Rate**: 14% (n=22/162) of eligible men declined participation.  **Reasons**:   - Lack of interest (n=14) - No need for info (n=5) - Uncomfortable with computers (n=3) - **Participation**: 91% (120/132) of enrolled men completed the survey - 79% (95/120) of men completing the survey used CD-ROM. - 64% of users returned tracking data; completed follow-up (91%; n=120)   **Dropout reasons:** N/A | **QoL:**  No significant differences found between groups on QOL or other outcomes  **Positive Effects:**  N/A  **Negative Effects:**  N/A  **Cost-Effectiveness:** N/A | **Setting:**   - Medical centre (n=1), Hospital (n=1), community sources (n=1) - CD mailed or used at research offices; laptops provided if needed   **Provider/Staff:**  Research staff facilitated consent & support; no clinical staff involved in delivering intervention | **Cost:** N/A  **Enablers:**   - Early timing post-diagnosis - lower desire for control - less prior knowledge   **Barriers:**   - Delays in access post-diagnosis - technical discomfort - prior saturation with info | N/A | - Only 64% of CD users returned usage tracking disks - Higher SES sample - No long-term follow-up or clinical decision outcome evaluation - Collapsing study arms may have reduced power to detect differences | CD-ROM DA was well accepted, especially by men with lower desire for control & less experience seeking information. These patients found DA helpful in preparing for treatment decision. |
| Thurtle 2021  **Country**:  UK  **Design**:  RCT  **Aim:**  Assess impact of the Predict Prostate risk communication tool on patient decision-making, disease perception, & treatment preferences | **Population:**  Men with newly diagnosed nonmetastatic PCa  **Sample Size**: N=145   - Intervention (n=75) - Control (n=70)   **Age**: Median 67 yrs (IQR 61–72)  **Stage**: Nonmetastatic  **Treatment**: Newly diagnosed, no prior treatment  **Other:** Living with partner (83%) | **Intervention name**:  Predict Prostate  **Supportive Care Type:** Personalized online prognostic model to communicate individualized risk estimates (PCa-specific & overall survival) & contextualized treatment benefits/harms.  **Mode of delivery:**  Structured presentation by trained researcher using Predict Prostate web tool  **Duration**: Not specified- between diagnosis appointment and follow-up. | **Preference measure (s):**   - Satisfaction - Usefulness - Perception   **Preference Tools:**   - Prognosis & treatment perception questionnaires   **QoL Tools:**   - State-Trait Anxiety Inventory (STAI) - DCS - Decision-Making Preference Questionnaire (DMPQ) | - 92% of intervention group found tool useful - 95% would recommend others - 36% reported being less likely to select radical treatment after viewing Predict - perceptions of mortality & treatment benefits became more realistic | **Sample Size:** N=145  **Refusal Rate** N/A  **Reasons:** N/A  **Participation Rate**: N/A | **QoL:** N/A  **Positive effects:**   - 26% lower decisional conflict intervention (p=0.027) - Improved scores on “support,” “uncertainty,” & “value clarity” DCS subscales - More accurate perceptions of PCa-specific & overall mortality   **Negative effects:**  N/A  **Cost-effectiveness:**  N/A | **Setting:**   - NHS centres (n=8) - Successfully integrated into routine diagnostic pathways   **Provider/Staff:**   - Trained researchers delivered intervention using script - Adoption limited to research team use | **Cost**: N/A  **Enablers:**   - Structured delivery - personalised prognostic output - online accessibility   **Barriers:**   - Possible pre-existing treatment decisions before exposure - potential contamination (SOC participants accessing tool online) | **N/A** | - Small sample size; underpowered to detect changes in treatment decisions - Not blinded, potential contamination in control group - No standardisation of SOC information between centres - No long-term follow-up or assessment of decisional regret - Excluded non-English-speaking & low-literacy populations | The Predict Prostate tool reduced decisional conflict & helped align patient expectations with realistic survival outcomes, without increasing anxiety. Most users found the tool helpful & recommended its use in clinical decision-making. |
| Trinh 2018  **Country**:  Canada  **Design**:  Single-arm feasibility  **Aim**:  Assess feasibility of a web-based physical activity intervention | **Population:**  PCa survivors currently on ADT, insufficiently active, recruited from two cancer centres in Toronto.  **Sample Size:** N=46  **Age (***M*) = 73.2 yrs (SD = 7.3**)**  **Stages:**   - Localised: 65.2%, - Metastatic: 32.6%   **Treatment:**   - ADT: Continuous (63.0%), Intermittent (32.6%) - Surgery: 54.3%, RT 82.6%, Chemotherapy: 6.5% - Completed treatment: 41.3%, Receiving treatment: 58.7%   Other:   - Married/common law: 84.8% - Race/Ethnicity: White 80.4% - *M* time since diagnosis: 93.6 months | **Intervention name**:  Reducing Sitting Every day for Treatment (RiseTx)  **Supportive Care Type:** Behaviour change support to reduce sedentary time & increase PA through self-monitoring, action planning, & goal setting. Participants were stepped through 5 progressive phases to reach a goal of more than 3000 steps/day/  **Mode of delivery:**   - Web-based application - Jawbone UP24 activity tracker   **Duration:** 12 weeks | **Preference measures:**   - Feasibility - acceptability - adherence   Preference Tools:   - Satisfaction survey - website analytics (e.g.,   logins)  **QoL measures:**   - FACT-G - FACT-F - FACT-P (QoL) | - >75% rated intervention satisfactory/ very satisfactory - Satisfied with simplicity, reminders, self-monitoring, tailored feedback, social support features - Overall adherence to logins (≥3/week): 72% - Step total decreased(35% in Phase 4; 26% in Phase 5) | **Sample Size:** 46  **Refusal Rate:**  29% (48/165) of all men screened declined participation. 51% (48/94) of eligible men chose not to participate.  **Reasons for Refusal:**   - Not interested (n=30) - other commitments (n=9) - too far (n=9).   **Participation:** 91% (42/46) completed the 12-wk intervention, & 97% (44/46) completed 12-wk survey.  **Dropout reasons:**   - heart attack (n=1) - feeling ill (n=1) - deceased (n=1) - carer responsibilities (n=1) | **QoL:**   - Non-clinical improvement on Emotional well-being (+1.2 points post-intervention (p = .018))   **Positive effects:**   - Decreased Sedentary time: −455.4 min/week (p = .005) - Increased MVPA: +44.1 min/week (p = .010) - Increased Step count: +1535 steps/day (p < .001)   **Negative effects:**   - 2 adverse events (MI & death) – not clearly related to intervention   **Cost Effectiveness**: N/A | **Setting:**  Genitourinary (GU) clinics in cancer centres (n=2)  Adoption limited to feasibility trial  **Provider/Staff**:   - Research coordinators administered intervention - Staff adoption N/A beyond research staff | **Cost: N/A**  **Enablers:**  Tailored support, wearable feedback  incentives  **Barriers:**   - Decreased in steps in later phases - long recruitment period - requirement for internet & tech use | N/A | - Small sample & no control group (feasibility design) - Selection bias (required internet access) - Unclear which intervention components were most effective - Limited generalizability | RiseTx was highly acceptable & aligned with participant preferences, supporting the feasibility of a tailored, web-based sedentary behaviour intervention for PCa survivors. |
| Viers 2015  **Country**:  USA  **Design:**  RCT  **Aim:**  Evaluate efficiency, satisfaction, & patient-incurred costs of remote video visits compared to office visits in post-RP patients. | **Population:**  Men who underwent RP for PCa, >90 days post-surgery, under surveillance  **Sample Size**:   - N= 55/295 - Video Visits (VV) = 28 - Office Visits (OV) = 27   **Age**: *M* = 62 yrs (VV = 62.5, OV = 61.5; SD = 8.2)  **Stage**: N/A  **Treatment**: RP (100%) | **Intervention name**:   - Video visit (VV) vs - Office visit (OV).   **Supportive Care Type**:  Post-surgical follow-up & surveillance  **Mode of delivery**:   - VV Group: Remote video consultation via secure telemedicine software - OV Group: Traditional in-person consultation   **Duration:** Single appointment for ~14.5 min | **Preference measures**   - Satisfaction   **Preference Tools:**   - 21-point patient perception questionnaire   **QoL Tools:**  N/A | - 88% of VV were satisfied and 100% of VV patients would repeat the experience | **Sample size:** N=70  **Refusal rate:**   - 40% (117/295) of screened individuals declined participation - 24% (70/295) of approached individuals enrolled in the study.   **Refusal reasons:**   - Unreachable (24%) - Declined (45%) - lacked equipment (16%) - uncomfortable with technology (10%) - medical reasons (8%).   **Participation rate**:   - Overall, 79% (55/70) of randomized individuals completed the study - 75% (27/36) of OV, - 82% (28/34) of VV.   **Dropout reasons:**  Technology limitations, medical necessity for physical visit, personal preferences | **QoL:** N/A  **Positive effects:**   - VV & OV equivalent (face time, wait time, total time devoted to care) - Median distance travelled: 0 miles (VV) vs. 95 miles (OV) - Travel time: 0 min (VV) vs. 95 min (OV) - Missed work: 0 days   **Negative Effects:**  N/A  **Cost-Effectiveness**:  Costs: $0 (VV) vs. $48 (OV); p < 0.0001 | **Setting**:  Tertiary care centre  Positive feedback on adoption, though  **Provider/Staff**:   - Urologists, residents - staff found VV less easily integrated into clinical workflow (62% vs. 76%; p = 0.05) | **Cost:** Lower cost per VV patient  **Enablers**:  Personalized scheduling  existing patient-provider relationship  PSA test via mail  **Barriers:**  Technology use barriers excluded some participants (16% lacked equipment; 10% uncomfortable with technology) | N/A | - Small sample size & limited generalizability - Selection bias: only 24% completed the study - Excluded those outside Minnesota/Wisconsin & lacking technology - Cost-effectiveness for the system not evaluated | Video visits were well-received & preferred by many patients, with comparable satisfaction & efficiency to office visits. Patients found VVs convenient & cost-saving, supporting their inclusion in future healthcare delivery models. |
| Wang 2020  **Country:**  USA  **Design**: Qualitative  **Aim**:  Develop data-driven grounded theory about engagement with web-based diet & exercise interventions | **Population:**  PCa survivors who completed a 12-week web-based intervention & participated in post-study focus groups  **Sample Size**: N=20 (from N=202 randomised in the main study)  **Age**: Median = 70 yrs (IQR 66–74)  Cancer stage:   - T2 (50%)   **Treatment**:   - RP (50%) - RT (55%)   Other:   - Race/white (95%) | **Intervention name**:  Community of Wellness  **Supportive Care Type: Web-based** lifestyle behaviour support with 4 levels:   - Level 1: Website access - Level 2: Website + personalized recommendation - Level 3: Level 2 + Fitbit + SMS reminders - Level 4: Level 3 + phone coaching (trainer & dietitian)   **Mode of delivery:** Online  **Duration**: 12 weeks | **Preference Measures:**   - Motivation - Usability & engagement - Feasibility - Acceptability - Behaviour Change   **Preference Tools:**   - focus groups guided by grounded theory - Feedback on specific tools (website, texts, Fitbit, coaching**)**   **QoL Tools:**  N/A | - Highly rated features: consistent communication, evidence-based education resources, tailored & interactive tools, human contact (coach/peer support) - Engagement influenced by environment (e.g., family cooking, travel, obligations**)** - Motivators: accountability & individualized support - Preferences: Varied by tech literacy and prior experience; personalized, adaptive content favoured over static tools. Coaching & community support highly valued, especially in higher support levels | **Sample Size:**  N=20  **Refusal Rate:** Not explicitly stated; of those who showed interest in focus groups,  25% (48/190) were willing to participate  **Reasons for Refusal:**   - Scheduling conflicts - not detailed   **Participation:** 42% (20/48) of men who were willing to participate actually participated in focus groups.  **Dropout reasons**: N/A | **QoL:**  N/A  **Positive Effects:**   - Some participants reported sustained lifestyle changes months after the intervention. - Enhanced awareness of health behaviours   **Negative effects:**   - Technological barriers for older adult - Limited clarity or personalization in some tools (e.g., Fitbit, texts)   **Cost-Effectiveness:** N/A | **Setting:**   - Health and science universities (n=3) - Applied in institutions.   **Provider/Staff:**   - Registered dietitians and certified exercise trainers (Level 4) provided tailored 1-on-1 coaching; Researchers, health educators & clinicians involved in creating website content and study protocols. - Staff adoption N/R | **Cost: N/A**  **Enablers:**   - Personalized messaging - ease of access - flexible content - trusted resources   **Barriers:**   - Competing priorities - low tech literacy - unclear role of coaches - device difficulties   **Other:**  Grounded theory developed to inform future digital interventions for PCa survivors. | N/A | .   - Small, self-selected focus group sample (10% of trial participants) - Predominantly White, educated population. - No theoretical sampling to ensure data saturation. - Recall bias due to 7-month delay between intervention & focus groups. | Web-based interventions for PCa survivors should be intuitive, customizable, & support-rich to promote lasting engagement. Tailoring, trust, feedback, & social support emerged as central to user preferences & long-term adoption |
| Wang 2021  **Country**: China  **Design**:  Single-arm feasibility  **Aim**:  Develop & test accessibility of mHealth app for PCa management, integrating patient self-management & physician decision support. | **Population:**  Men with PCa receiving treatment & follow-up at Haikou Hospital  **Sample Size:** n=28 completed  **Age:** Median = 72.5 yrs (IQR 67.5–79.5)  **Stage:**   - Stage I: 7% (2) - Stage II: 39% (11) - Stage III: 25% (7) - Stage IV: 29% (8)   **Treatment:**   - RP: 39% (11)   **Other:**   - Married: 71% (20) - Bachelor’s degree or higher: 36% (10) | **Intervention name**: RyPros app  **Supportive Care Type:** Digital self-management & clinician decision-support tool featuring dynamic visualisations (of PSA, testosterone, health metrics), reminders (e.g., medication) and instant messaging (clinician-patient)  **Mode of delivery:** Smartphone app  **Duration:**4 weeks | **Preference measures**:   - Accessibility - Acceptability   **Preference Tools:**   - Weekly log-in & questionnaires - Satisfaction & acceptability survey - Feedback on core features   **QoL:**   - EPIC-26 - numerical rating scale NRS (pain) - MAX-PC (anxiety) - Burns Depression Checklist BDC (depression) | *Accessibility*  Average logins: 2.82 times/week; questionnaire completion: 0.89/week  *Acceptability:*   - 71% satisfied/ very satisfied. - 64% liked app. - 61% intended to continue use. - Preferred features: Instant messaging: 71%; Reminders/assessments: 64%; Visualization: 57% | **Sample Size:** n=28  **Refusal Rate & reasons:** N/A    **Participation Rate:** (87.5%; n=28/32).  **Dropout reasons:**  Personal reasons | **QoL: N/A**  **Positive Effects:**   - High usability & moderate engagement - Increased awareness & interest in health management   **Negative Effects:**  **N/A**  **Cost-Effectiveness:** N/A | **Setting**:  Hospital urology department  Successfully deployed and used in clinical workflow.  **Provider/Staff:**   - Physicians and nurses trained in app facilitation. - Clinicians engaged in communication, monitoring, and decision-making via the RyPros doctor interface**.** | **Cost:**  Not detailed; participants received ¥100 gift card.  **Enablers:**  Visual simplicity, multi-functional tools, education, messaging  **Barriers:**   - Moderate satisfaction with visualization (likely due to limited eHealth literacy) - Need for broader functionality testing & UI improvements. | **Organizational/Staff Level:**  N/A    **Individual level:**  61% intended continued use, long-term retention unknown. | - Small sample size, short follow-up (4 weeks) - No control group - Acceptability data mostly short-term & self-reported - Moderate UI satisfaction, some complexity for older users | RyPros app showed high initial acceptance among PCa patients. Participants valued communication features & reminders most, while visualization features were less favoured, suggesting a need for enhanced user-centred design for older or low-literacy users. |
| Wennerberg 2023  **Country**:  Sweden  **Design**:  RCT  **Aim**:  Evaluate effect of self-care web-based/mobile app after RP on urinary continence, sexual function, & self-care adherence | **Population:**  Men with intermediate- or high-risk LPCa undergoing RP at 3 Swedish county hospitals.  **Sample Size**: N=170 randomized (85 intervention, 85 control); 146 included in the final analysis  **Age**: *M* = 64 yrs (SD ≈6.2)  **Treatment**:   - Bilateral nerve-sparing surgery: (47.9%) - Unilateral (32.1%) - No nerve-sparing: (20%) | **Intervention name**:  electronic Patient Activation in Treatment at Home (ePATH)  **Supportive Care Type:** Digital self-care platform with tailored modules for pelvic floor muscle exercises, physical activity, symptom tracking, goal setting, & secure messaging; provided alongside standard care.  **Mode of delivery:** Web & mobile app **standard**  **Duration:**  12-month access post-surgery | **Preference measure:**   - Adherence - Effectiveness of intervention   **Preference Tools:**   - Log data (login frequency, self-care activity registration) - Self-assessed questionnaire   **QoL measures:**   - EPIC (urinary & sexual domains) - Saltin-Grimby Physical Activity Scale - Self-reported pelvic floor muscle exercises | **Engagement:**   - 64% in intervention used ePATH median use score= 70 (IQR 6-2330) - Of 53 users, 68% used ePATH during first month postoperatively - Usage declined over time: 32% at 3 month, 21 at 6 months, 11% at 12 months - Gamification & more interactive feedback recommended | **Sample Size:** N= 170 randomised  **Refusal Rate:** 51% (176/346) of eligible individuals declined to participate.  **Reasons:** N/A  **Participation Rate:**  Overall, 86% (146/170) of randomized individuals completed follow-up. Intervention: 85% (72/85) completed follow-up.  Control: 87% (74/85) completed follow-up**.**  **Dropout reasons: N/A** | **QoL:**   - Urinary continence & sexual functioning improved over time in both groups (no significant between-group difference, P>.09)**Positive Effects**: - Time positively associated with recovery in both outcomes - Bilateral nerve-sparing & younger age correlated with better outcomes   **Negative Effects**:  Pelvic floor muscle exercise adherence decreased over time  **Cost-Effectiveness**: evaluated/A | **Setting:**   - hospitals with standardised PCa care trajectories (n=3) - Integrated into real-world workflow without altering existing services   **Provider/Staff:**   - CNS individualised modules & monitored ePATH accounts - Nurses involved in onboarding; however, but no staff adoption data | **Cost:** N/A  **Enablers:**  Tailored content, reminders, symptom tracking, secure messaging  **Barriers:**  Low long-term adherence, motivation, & digital fatigue; standard follow-up routines may have reduced perceived need | **Organizational/Staff Level:**  Not sustained beyond study  **Individual level:**  Declining engagement indicates low long-term maintenance without ongoing facilitation | - Underpowered due to recruitment shortfall (planned N=228) - High attrition & declining engagement - Did not assess digital literacy or attitudes, or include all EPIC domains despite powering for them - No blinding possible; self-report bias possible - Did not control for penile rehabilitation medication use | - The ePATH app did not significantly improve urinary or sexual outcomes but showed moderate acceptability & initial engagement. - Tailored, timed, & motivationally supportive features may be necessary to sustain long-term digital self-care adherence in PCa recovery. |
| Wootten 2014  **Country**: Australia  **Design**: Pre-post feasibility  **Aim**: Develop & assess feasibility, usability, & acceptability of online self-directed psychological intervention for men treated for LPCa | **Population**:  Men treated for LPCa within the last 5 yrs  **Sample Size:** N= 64  **Age: *M* =** 62 yrs (range 46–78)  **Cancer Stage:** Localized PC  **Treatmen**t:   - RP: 98% - Brachytherapy: 2%   **Other:**  Married/partnered: 89%  University education: 41%  Prior access to support:   - Psychologist: 9%, - Physiotherapist: 31% - Nurse: 42%, - Support group: 14% | **Intervention name**: My Road Ahead  **Supportive Care Type:** Self-directed online psychological support (CBT-based) with 6 modules, an interactive mood monitor and moderated forum to support emotional & sexual wellbeing  **Mode of delivery:** Online  **Duration:** 6–12 weeks | **Preference measures:**   - Satisfaction - Useability - forum use   **Preference Tools:**   - Participant feedback - Program satisfaction survey (custom**)**   **QoL measures:**   - Depression Anxiety Stress Scale (DASS-21) - IIEF | *Satisfaction:*   - 47.8% rated satisfaction ≥6/10 - 78.3% would recommend to others - High appreciation for thought-behaviour links & real-life stories   *Forum Use:*   - 41.4% satisfaction (≥6/10) - 65.5% found it easy to use - Value in peer normalisation, but low post volume cited as barrier | **Sample Size:** 64  **Refusal Rate & Reasons:** N/A  **Participation Rate:**  85% (64/75) of individuals registered to participate completed the study; 59% (44/75) provided intervention feedback.  **Dropout reasons: N/A** | **Quality of Life:**   - 67% had severe erectile dysfunction - 15% reported moderate-severe depression, 11% anxiety   **Positive Effects**:   - High acceptability - some reported enhanced insight & emotional processing   **Negative Effects**:   - Gated module progression decreased motivation - Partner engagement low despite intended design   **Cost-Effectiveness**: N/A | **Setting:**  Online & home-based; participants accessed independently  **Provider/Staff:**   - Clinicians, psychologists, urologists developed intervention; forum moderated by the researcher - Staff adoption N/R | **Cost: N/A**  **Enablers:**   - Anonymity - flexibility& convenience - self-paced learning   **Barriers:**   - Module lock/unlock sequencing - ow forum activity - technical issues - need for reminders   **Other**   - Post pilot-changed included weekly emails, open module navigation, mood check-ins and forum moderator posts - Younger sample than typical PCa population | **N/A** | - Small sample & no control group - No analytics on user engagement beyond self-report - Low forum participation - No long-term follow-up or outcome assessment | Men with PCa found *My Road Ahead* acceptable & moderately satisfying. Participants valued flexible, anonymous access & emotional reflection. Preferences highlight need for tailored support, reminder systems, & optional partner engagement in digital psychosocial interventions. |
| Wootten 2017  **Country**: Australia  **Design**: Three-arm RCT  **Aim**:  Evaluate efficacy of online psychological intervention in improving sexual satisfaction among men treated for LPCa | **Population:**  Men with LPCa, treated with curative intent within the past 5 yrs  **Sample Size**: N=142   - Intervention (MRA): N=47 - MRA + Forum: N=48 - Forum: N=47   **Age**: *M*= 61yrsyrs (SD = 7yrs)  **Treatment:**  RP: 88% | **Intervention name**:  My Road Ahead (MRA)  MRA alone (MRA Only), MRA plus access to an online forum  (MRA + Forum), & access to the forum alone (Forum).  **Supportive Care Type:**  CBT-based self-guided psychological intervention addressing emotional, physical, sexual, & identity-related concerns offered over 6 online modules.  **Mode of delivery**: Online  **Duration**:  10 weeks | **Preference measures:**   - Use   **Preference Tools:**  Module/forum use  **QoL measures:**   - DASS-21 - PCa–related QoL scale (including masculine self-esteem, sexual confidence, etc.) | *Use*   - Module completion*:* Similar in MRA (60%) & MRA+Forum (57%) - Forums: Higher engagement in Forum (2-3 posts/user) than MRA+Forum (1-2 posts). - 62% of all forum users spent <30 min/week (mostly "lurking") | **Sample Size:** N=142  **Refusal Rate & Reasons**: N/A  **Participation Rate:** N/A | **QoL:** N/A  **Positive effects:**   - Significant and sustained sexual satisfaction improvement in MRA+Forum at 3 & 6-monthsMRA (P = .004;).   No significant improvement in other groups.   - Significant decrease in sexual aid use for MRA + Forum group (P = .021); non-significant for MRA & Forum - Significant improvements in MRA+Forum in erectile function, masculine self-esteem sexual confidence (*P*< .044)in other groups   **Positive effects:**  N/A  **Negative effects:**  N/A  **Cost-effectiveness**  N/A | **Setting:**  Web-based, fully remote  Not implemented in clinical settings  **Provider/Staff:**   - Minimal staff involvement aside from moderation | **Cost: N/A**  **Enablers:**   - Topic relevance - Anonymity - structured modules - CBT content   **Barriers:**  Low engagement with forum; small sample size | N/A | - Small sample size limited power for subgroup analysis - Overrepresentation of surgical patients; underrepresentation of other treatment types - No tailoring based on time since treatment - Did not reach normative sexual satisfaction levels despite improvement | Participants in the combined MRA + Forum condition experienced the most significant & sustained improvements in sexual satisfaction, confidence, & masculine self-esteem. The combination of structured CBT modules & peer discussion appears to be essential in helping men adjust to sexual changes post-treatment. |
| Yanez 2015  **Country**: USA  **Design**: Two-arm RCT  **Aim**: Assess feasibility, acceptability, & preliminary efficacy of technology-assisted stress management intervention vs attention-control health promotion in racially diverse men with advanced PCa | **Population:**  Men aged ≥50 with stage III or IV PCa  **Sample Size**: N=74   - CBSM: 37 - HP: 37   **Age**: M= 68.8yrs (range 52–94yrs)  Cancer Stage:   - Stage III (49%) - Stage IV (51%)   **Treatment:**   - ADT ± RT in past 6 months - No surgery or chemotherapy in past 6 months   **Other**:   - White (57%) - Married/partnered (65%) - Retired (58% - College grad (49%) | **Intervention name**:  Cognitive-Behavioural Stress Management (CBSM)  **Supportive Care Type:** Psychosocial support for stress management, depression, & HRQOL   - CBSM: Relaxation & stress reduction techniques + coping skills - HP: Health education content (e.g., sleep, diet, activity), no psychosocial elements   **Mode of delivery:** Web-based video conferencing on provided tablets  **Duration:**  10 weekly 90-minute sessions | **Preference measures:**   - Feasibility - Acceptability   **Preference tools:**   - Weekly evaluation of confidence & group experience   Exit survey on content usefulness & satisfaction  **QoL measures:**   - PROMIS - FACT-G - MOCS | *Acceptability:*   - Weekly CBSM ratings (out of 5) high for confidence using new information (3.92–4.19) and group support (4.05–4.19). - Exit survey scores (out of 5): 3.65–3.81 for CBSM; 3.40–3.68 for HP. - Black participants reported significantly higher satisfaction than White participants (P = .04) | **Sample Size:** N=74  **Refusal Rate:**  14% (166/1207) of screened individuals refused participation.  **Refusal Reasons:**   - time burden - low interest.   **Participation Rate:**  Overall: 82% (61/74) of randomized participants completed 6-mo follow-up. CBSM intervention: 81% (30/37).  Control: 84% (31/37)]  **Dropout reasons:** N/A | **QoL:**   - CBSM group had clinically meaningful and significantly fewer depression symptoms at 6 months (*p*=.03) - Non-significant Functional wellbeing improvement (P = .06) but clinically meaningful difference (≥2 points) - Significant relaxation self-efficacy improvement for CBSM group (*P* < .01), large effect size (d = 1.87**)** - No significant differences in total HRQOL or distress | **Setting:**   - Hospital (n=1) & Medical Centre (n=1) - Provided tablets & internet access to support participation   **Provider/Staff:**   - Master’s-level facilitators; trained & supervised by licensed psychologists. - Fidelity monitored via recorded sessions & supervision. | **Success:** Moderate to high session attendance **(**CBSM: *M* = 6.6/10; HP: *M* = 8.2/10 (*P* = .04))  **Enablers:**   - training for tech use - flexible scheduling - race-equity approaches.   **Barriers:**  Cancer-related avoidance linked to lower CBSM attendance. | **Organizational Level:** Intervention under evaluation; N/A post-study  **Individual Level:** Improvements in depressive symptoms & functional well-being sustained at 6-month follow-up. | - Small sample size, underpowered for definitive efficacy - Provided devices limits generalizability to real-world users - Predominantly non-surgical treatment cohort - Recruitment challenges among minority & advanced-stage patients | CBSM intervention was feasible & well accepted, particularly among Black participants. Participants preferred the psychosocially supportive format, with CBSM yielding higher ratings for confidence, engagement, & relaxation benefits than the HP condition. Preferences were shaped by peer support, accessible delivery, & perceived usefulness of stress management techniques. |
| Young 2023  **Country**:  Canada  **Design**: Qualitative  **Aim**:  Inform design & implementation of nurse-led digital therapeutic for PCa follow-up care | **Population:**  PCa survivors  **Sample Size**: N=20 Survivors (n=10)  HCPs n=10)  **Age**: *M* = 66 yrs  **Stage**: N/A  **Treatment**: N/A  **Other**: White (70%) | **Intervention name**:  Ned Nurse  **Supportive Care Type:** Digital therapeutic platform for post-treatment survivorship care focused on education, self-management, support & facilitating care coordination and assessment.  **Mode of delivery:** Online platform. | **Preference Measures:**   - Acceptability - Feasibility - Appropriateness - Adoption   **Preference Tools:**   - Semi-structured interviews - CFIR & Proctor's implementation outcomes - Readiness checklist   **QoL Tools:**   - PROs on QoL - Interviews + optional follow-up care and virtual care models | *Acceptability*   - Valued regular follow-up for reassurance & continuity. - Preferred a nurse as main contact if well trained and available. - Emphasized need for mental health & peer support. - Appreciated role clarity in care delivery.   *Feasibility*   - Devices used: 60% used laptops/desktops. - Main uses: Information seeking (100%), storing info (100%), communication (80%), scheduling (80%), accessing health services (50%).   *Appropriateness*   - Desired clearer timelines, education materials, and symptom self-management support - Importance on support aligned with individual needs, especially around mental health and peer connection.   *Adoption*   - High engagement with digital tools suggests openness to digital intervention when relevant & accessible. | **Sample size:** N=20  **Refusal:** N/A  **Participation:** 100%  **Dropout reasons:** N/A | **QoL: N/A**  **Positive effects:**  Virtual model seen as beneficial for reassurance, symptom tracking, & timely access to support  **Negative effects:** N/A  **Cost effectiveness:** N/A | **Setting:**   - Three health network organisations - Virtual nurse-led care seen as feasible, appropriate, and acceptable by both healthcare providers and patients. Aligned with current healthcare demands and resource limitations.   **Provider/staff:**   - 8 Registered Nurses and 2 Urology Specialists**.** - Nurses support the model if given adequate training and integration into existing workflows. | **Cost: N/A**  **Enablers:**  Training, technical support, role clarity, integration into workflows  **Barriers:**  Digital literacy, usability, workload, & unclear patient expectations | **Organisational Level:** Design still in early phases; implementation success to be validated.  **Individual Level:** N/A | - Small, urban, & highly literate sample - Conducted during COVID-19 digital transition phase. - Not generalizable to lower-income or non-English-speaking population - No actual intervention deployed (pre-implementation phase) | Patients & providers supported a nurse-led virtual follow-up care model. Preferences included continuity, role clarity, self-management support, & personalized care delivery. Readiness checklists were developed for implementation based on CFIR & Proctor frameworks. |
| Zhang 2015  **Country**:  USA  **Design**:  Three-arm RCT  **Aim**:  Assess efficacy of pelvic floor muscle exercise combined with biofeedback (BF) symptom self-management, delivered via peer support or phone, on improving urinary continence & QOL | **Population:**  Men with early-stage PCa & persistent urinary incontinence (≥6 months post-treatment)  **Sample Size**: N=279   - BF + Support (n=81) - BF + Telephone (n=81) - Usual Care (n=82)   **Stage**:   - Stage I–II (95%)   **Treatment**:   - Surgery: 53%   **Age**: *M* = 64–67 yrs  **Other:**   - Black (33–39%) - Married (63-67%) - College or greater (33–43%) | **Intervention name**:  Stay Dry  **Supportive Care Type:** PFME + problem-solving therapy (PST)-based symptom self-management  **Mode of delivery:**  phone  **Duration:** 9 months | **Preference measures:**   - Adherence   **Preference Tools:**   - Patient Diaries   **QoL measures:**   - UCLA Prostate Cancer Index UCLA-PCI (urinary function & bother) - VAS for perceived severity - International Prostate Symptom Score I-PSS (symptom severity) | Completion rate 87%)   - Attrition (13%) | **Sample size**: N=279  **Refusal rate**: 19% (253/1331) of patients contacted for screening refused enrolment; 22% (289/1331) enrolled in the study.  **Refusal reasons**: N/A  **Participation**: Individuals completing study from those randomized:  Overall: 87% (244/297)  BF + Support: 89% (81/91)  BF + Telephone: 86% (81/94)  UC: 87% (82/94)  **Dropout reasons:**   - Worsening health condition - family - economic condition | **QoL:**   - Leakage Frequency Significantly reduced at 3 months in both intervention groups vs UC. - Leakage Amount significant reduced at 6 months only in BF + Support | **Setting:**   - Multiple hospitals systems (number N/R) - Successfully implemented at multiple clinical and academic centres with coordinated cross-site logistics. Conducted in controlled research environments; not tested for real-world health system integration.   **Staff:**   - Trained biofeedback technician; nurse specialist & psychologists for PST - Highly trained staff delivered interventions with demonstrated fidelity (manualized and monitored). | **Cost: N/A**  **Enablers:**   - Accessible (especially for elderly) - group support benefit. - low burden intervention - Strong protocol fidelity (audio recording, logs, training manual) - Equal delivery quality across support & phone arms   **Barriers:** N/A | **N/A** | - Short 3-month intervention period - No long-term sustainability analysis - Limited generalizability beyond urban, English-speaking, early-stage PCa survivors - Missing data at 6 months could affect outcome interpretation. - I-PSS may not fully capture post-PCa incontinence nuances. - Natural recovery in UC group high baseline functioning limited observable change potential bias due to self-reported measures. | The combination of PFME & symptom management delivered in a peer group setting significantly improved urinary continence & quality of life. While the telephone format showed promise, peer support led to more robust long-term outcomes. Preferences for group-based, low-burden interventions suggest a feasible path forward for behavioural incontinence care in PCa survivorship. |
